# Cost-Effectiveness of Salt Substitute and Salt Supply Restriction in Eldercare Facilities: The DECIDE-Salt Cluster Randomized Clinical Trial

**DOI:** 10.1001/jamanetworkopen.2023.55564

**Published:** 2024-02-12

**Authors:** Xiaozhen Lai, Yifang Yuan, Hongxia Wang, Ruijuan Zhang, Qianku Qiao, Xiangxian Feng, Aoming Jin, Huijuan Li, Jiayu Li, Lei Si, Pei Gao, Stephen Jan, Hai Fang, Yangfeng Wu

**Affiliations:** 1 Department of Health Policy and Management, School of Public Health, Peking University, Beijing, China; 2 Health Economics Research Centre, Nuffield Department of Population Health, University of Oxford, Oxford, United Kingdom; 3 Peking University Clinical Research Center, Peking University First Hospital, Beijing, China; 4 Department of Epidemiology and Biostatistics, Peking University School of Public Health, Beijing, China; 5 Department of Nutrition and Food Safety, Hohhot Center for Disease Control and Prevention, Inner Mongolia, China; 6 Department of Public Health, Xi’an Jiaotong University, Shaanxi, China; 7 Yangcheng Ophthalmic Hospital, Shanxi, China; 8 Changzhi Medical College, Shanxi, China; 9 Now with China National Clinical Research Center for Neurological Diseases, Beijing Tiantan Hospital, Capital Medical University, Beijing, China; 10 School of Health Sciences, Western Sydney University, Campbelltown, New South Wales, Australia; 11 Translational Health Research Institute, Western Sydney University, Penrith, New South Wales, Australia; 12 George Institute for Global Health, University of New South Wales, Sydney, New South Wales, Australia; 13 China Center for Health Development Studies, Peking University, Beijing, China; 14 Peking University Health Science Center-Chinese Center for Disease Control and Prevention Joint Research Center for Vaccine Economics, Beijing, China; 15 Institute for Global Health and Development, Peking University, Beijing, China

## Abstract

**Importance:**

Salt substitution has been reported to be a cost-saving sodium reduction strategy that has not yet been replicated in different contexts.

**Objective:**

To estimate the cost-effectiveness of sodium reduction strategies within the DECIDE-Salt trial.

**Design, Setting, and Participants:**

The DECIDE-Salt trial cluster randomized in a 1:1:1:1 ratio 48 eldercare facilities in China into 4 groups for evaluation of 2 sodium reduction strategies for 2 years: 1 with both strategies, 2 with either strategy, and 1 with neither strategy. The trial was conducted from September 25, 2017, through October 24, 2020.

**Interventions:**

The 2 intervention strategies were replacing regular salt with salt substitute and progressively restricting salt supply to kitchens.

**Main Outcomes and Measures:**

The main outcomes included per-participant costs of intervention implementation and medical treatments for hypertension and major adverse cardiovascular events (MACEs) against mean reductions in systolic blood pressure, hypertension prevalence, MACE incidence, and mortality. The incremental cost-utility ratio was then assessed as the additional mean cost per quality-adjusted life-year gained. Analyses were conducted separately for each strategy, comparing groups assigned and not assigned the test strategy. Disease outcomes followed the intention-to-treat principle and adopted different models as appropriate. One-way and probabilistic sensitivity analyses were conducted to explore uncertainty, and data analyses were performed between August 13, 2022, and April 5, 2023.

**Results:**

A total of 1612 participants (1230 males [76.3%]) with a mean (SD) age of 71.0 (9.5) years were enrolled. Replacing regular salt with salt substitute reduced mean systolic blood pressure by 7.14 (95% CI, 3.79-10.48) mm Hg, hypertension prevalence by 5.09 (95% CI, 0.37-9.80) percentage points, and cumulative MACEs by 2.27 (95% CI, 0.09-4.45) percentage points. At the end of the 2-year intervention, the mean cost was $25.95 less for the salt substitute group than the regular salt group due to substantial savings in health care costs for MACEs (mean [SD], $72.88 [$9.11] vs $111.18 [$13.90], respectively). Sensitivity analysis showed robust cost savings. By contrast, the salt restriction strategy did not show significant results. If the salt substitution strategy were rolled out to all eldercare facilities in China, 48 101 MACEs and 107 857 hypertension cases were estimated to be averted and $54 982 278 saved in the first 2 years.

**Conclusions and Relevance:**

The findings of this cluster randomized clinical trial indicate that salt substitution may be a cost-saving strategy for hypertension control and cardiovascular disease prevention for residents of eldercare facilities in China. The substantial health benefit savings in preventing MACEs and moderate operating costs offer strong evidence to support the Chinese government and other countries in planning or implementing sodium intake reduction and salt substitute campaigns.

**Trial Registration:**

ClinicalTrials.gov Identifier: NCT03290716

## Key Points

**Question:**

Is it cost-effective to replace regular salt with salt substitute and to restrict salt supply to kitchens in eldercare residential facilities?

**Findings:**

In this cluster randomized clinical trial of 1612 older adults residing in eldercare facilities, the salt substitution intervention showed considerable potential to be cost-saving, whereas the salt restriction strategy did not show significant results. The salt substitution cost savings were estimated to be large if the intervention were rolled out to all eldercare facilities in China.

**Meaning:**

These findings may support government planning and implementation of public health programs to replace regular salt with salt substitute in eldercare facilities in China and other countries.

## Teaser

This economic evaluation of the DECIDE-Salt cluster randomized clinical trial estimates the cost-effectiveness of salt reduction strategies in eldercare facilities in China.

## Introduction

Cardiovascular disease is a primary contributor to mortality in China and is increasing.^1^ Hypertension, the major cause of cardiovascular disease, results in approximately two-thirds of stroke incidents and nearly half of ischemic heart disease cases.^2^ Evidence suggests that reducing sodium intake and increasing potassium intake to reduce blood pressure^3,4,5^ may prevent cardiovascular disease and reduce mortality.^6,7,8^

Population aging is a primary contributor to the increasing burden of cardiovascular disease worldwide. With effective sodium intake reduction strategies, eldercare facilities could help prevent and control hypertension and cardiovascular disease and promote healthy aging in their residents. In 2022, 40 587 eldercare facilities in China provided supportive services to approximately 2.1 million older adults with limited capabilities to live independently.^9^ The DECIDE-Salt (Diet, Exercise and Cardiovascular Health–Salt Reduction Strategies for the Elderly in Nursing Homes in China) trial,^10^ conducted among older adults collectively living in residential eldercare facilities, initially reported that replacing regular salt with salt substitute reduced mean systolic blood pressure (SBP) by 7.1 mm Hg and risk of cardiovascular events by 40%, while efforts to restrict salt supply did not reduce any study outcomes. Further investigations into the cost-effectiveness of the 2 interventions alongside this trial may inform decision-making for future large-scale implementation.

The Salt Substitute and Stroke Study (SSaSS) demonstrated that salt substitute is effective and cost-saving in reducing the risk of major cardiovascular events and total mortality among individuals at high cardiovascular risk.^11,12^ However, there is no evidence on the health benefits and cost-effectiveness of salt substitution for older adults living in collective settings.

Most previous health economic evaluations of salt reduction strategies other than SSaSS were model based, relying on extrapolated health outcomes that may be subject to a series of assumptions.^12,13,14,15,16^ Compared with older adults living in the community, those living in care facilities are provided with standardized meals prepared by facility kitchens, making it easier to conduct salt reduction interventions by controlling salt procurement and supply channels, accompanied by training for facility staff and residents. To address this research gap, we evaluated the cost-effectiveness of 2 feasible and scalable sodium reduction intervention strategies within the DECIDE-Salt trial.

## Methods

### Trial Design and Participants

The design of the DECIDE-Salt trial has been registered with ClinicalTrials.gov (NCT03290716) and reported previously.^10,17^ This economic evaluation was approved by the Peking University institutional review board along with the trial, and all participants provided written informed consent. The trial was conducted between September 25, 2017, and October 24, 2020; it followed key components of the Consolidated Health Economic Evaluation Reporting Standards (CHEERS) reporting guideline.^18^ The trial protocol appears in Supplement 1.

Briefly, 48 eldercare facilities were cluster randomized in a 1:1:1:1 ratio to 4 groups using a central computerized process, with stratification by region. One group received both salt substitution and salt supply restriction; 1 group, salt substitution only; 1 group, salt supply restriction only; and 1 group, neither strategy. The salt substitute (China Salt General Company) consisted of 62.5% (mg/mg) sodium chloride, 25% (mg/mg) potassium chloride, and 12.5% (mg/mg) dried food ingredients. Groups not receiving salt substitute received regular salt. Kitchens in the salt supply restriction groups received gradually reduced amounts of study salt (regular salt or salt substitute), with a 5% to 10% reduction every 3 months, to achieve a total reduction of 40% by the end of the intervention. In contrast, the usual supply groups did not restrict the study salt supply. All interventions continued for 2 years.

Eligible study participants included adults 55 years or older with blood pressure measured at the baseline survey. Randomization was done after baseline survey data had been collected and by an independent statistician (eFigure 1 in Supplement 2; see also the trial protocol in Supplement 1). Follow-up visits were completed at 6, 12, 18, and 24 months after randomization.

### Effectiveness Outcomes

Effectiveness outcomes included SBP change, hypertension prevalence, major adverse cardiovascular events (MACEs) adjudicated as definite (nonfatal stroke, nonfatal myocardial infarction, hospitalized nonfatal heart failure, or cardiovascular death), and quality-adjusted life-years (QALYs). Blood pressure was measured at baseline and each follow-up visit, and hypertension was defined as SBP of 140 mm Hg or higher and/or diastolic blood pressure (DBP) of 90 mm Hg or higher or taking antihypertensive medication in the preceding 2 weeks.^19^ MACEs were adjudicated by a central committee masked to the randomized assignment according to information collected by local staff, including hospitalized or reported possible cardiovascular events, with additional information collected for end point adjudication. The QALYs were derived using the EuroQoL 5-Dimension 3-Level instrument at the baseline and 1- and 2-year visits,^20,21^ and the discounted lifetime QALY loss of cardiovascular deaths was included with a discount rate of 5%.^22^

### Costs

Operating cost data were collected once every 3 months during the trial and aligned with the intervention delivery schedule to measure the cost of planned interventions with reasonable accuracy.^23^ The per-participant operating cost comprised 2 parts: the province-level cost before and during intervention implementation reported by the intervention managers in each region (eTables 1.1 and 1.2 in Supplement 2) and the facility-level cost incurred throughout the implementation process as reported by facility managers (eTable 1.3 in Supplement 2). Salt consumption cost per participant was based on the market price of the study salt and mean quantity of salt consumed. Labor cost was measured by multiplying hours spent and hourly wage separately for province-level managers, facility managers, procurement personnel, and chefs based on data obtained from their employers.

Health care costs included medical treatment for MACEs and antihypertension medications. Medical costs of hospitalized MACEs were extracted from the health statistical yearbook,^24^ which reports the mean inpatient cost of treating stroke, acute myocardial infarction, and congestive heart failure in China. Information on antihypertension medication use was collected at baseline and 6, 12, 18, and 24 months, and medication cost was based on the procurement price of antihypertension drugs from the MENET website.^25^ Overall, the per-participant mean cost comprised operating (province-level operating cost + facility-level operating cost + labor cost of managers and chefs), salt consumption (unit cost × mean quantity consumed), and health care (mean treatment cost for MACEs + mean cost for antihypertension medication) costs. All costs are presented in 2020 US dollars ($1 = ¥6.90) and adjusted for inflation when necessary.^22^ Future costs were not involved in this retrospective calculation, as the deaths of eldercare facility residents did not result in work productivity losses.

### Statistical Analysis

The data analysis was completed between August 13, 2022, and April 5, 2023. Analyses were conducted separately for each intervention strategy in which 2 groups assigned and 2 groups not assigned to the test strategy were compared.

Analyses of effectiveness outcomes followed the intention-to-treat principle and accounted for a clustering effect at the facility level. Specifically, we adopted a linear mixed model for SBP and QALYs with adjustment for baseline value and a generalized linear mixed model for hypertension prevalence, MACEs, and cardiovascular deaths, as described in previous studies.^17,26^

An economic evaluation prespecified in the trial protocol (Supplement 1) was conducted from the societal perspective along with the 2-year follow-up of the DECIDE-Salt study. Both cost-effectiveness and cost-utility analyses were done to examine the costs and health benefits at 1 and 2 years. The incremental cost-effectiveness ratio (ICER) was calculated for the effect of reducing SBP, prevalence of hypertension, and risk of MACEs and cardiovascular deaths. The incremental cost-utility ratio (ICUR) was then assessed as the additional mean cost per QALY gained during the trial. The widely acknowledged cost-effectiveness threshold equivalent to the gross domestic product per capita ($10 435 in 2020) was adopted.^27^

We then conducted 1-way and probabilistic sensitivity analyses to measure the robustness of results. One-way variations included (1) using alternative discount rates of 0% and 8% as recommended,^22^ (2) including only facility-level cost or merely salt cost for operating cost, (3) using the lowest and highest market price of salt substitute in China,^28^ (4) varying MACE treatment costs from 0% to 20% above or below the base case value, (5) varying antihypertension medication costs from 0% to 20% above or below the base case value, and (6) adjusting QALY change to 20% above or below the base case value. In the probabilistic sensitivity analysis using Monte Carlo simulation (n = 10 000 iterations), the plausibility range was assumed to be 25% above or below the base value in the absence of reliable uncertainty ranges for parameters.

Using trial-based costs and health benefits, estimations were made for rolling out salt substitute to all registered eldercare facilities in China.^9^ Analyses were performed using Stata SE, version 17 (StataCorp LLC) and Microsoft Excel, version 16.56 (Microsoft Corporation) statistical software. An incremental effect for which the 95% CI excluded 0 was considered significant.

## Results

In all, 1612 eligible participants (1230 males [76.3%] and 382 females [23.7%]) were enrolled from 48 facilities (mean [SD] age, 71.0 [9.5] years; SBP, 137.5 [21.3] mm Hg; and DBP, 80.5 [11.6] mm Hg). Baseline characteristics are given in eTables 2 and 3 in Supplement 2, and follow-up status has been described previously.^17^

### Costs of Interventions

Table 1 presents the mean cost per participant at different stages of intervention. Analysis at 1 year indicated that the overall cost was $42.44 with salt substitute, $52.21 with regular salt, $43.04 with a restricted supply, and $52.04 with a usual supply; the MACE treatment cost accounted for a substantial proportion of the total cost (mean [SD], $37.44 [$4.68], $54.59 [$6.82], $41.19 [$5.15], and $51.28 [$6.41] per participant, respectively). At 2 years, the cost increased to $76.27 for salt substitute, $102.22 for regular salt, $89.66 for a restricted supply, and $88.94 for a usual supply.

**Table 1.** Mean Cost for Each Participant at Different Stages of the Intervention

Cost item	Cost, mean (SD), $^a^			
	Salt substitute vs regular salt		Restricted supply vs usual supply	
	Salt substitute	Regular salt	Restricted supply	Usual supply
**For 1 y**				
Overall cost	42.44	52.21	43.04	52.04
Operating costs	12.19 (5.38)	4.45 (2.18)	8.84 (5.83)	7.81 (5.52)
Provincial-level subtotal	1.01 (1.18)	0.34 (0.62)	0.97 (1.11)	0.38 (0.78)
Labor	0.24 (0.18)	0.12 (0.17)	0.29 (0.19)	0.07 (0.08)
Transportation	0.78 (1.16)	0.22 (0.54)	0.68 (1.09)	0.32 (0.74)
Facility-level subtotal	11.18 (5.46)	4.11 (2.10)	7.87 (5.84)	7.42 (5.11)
Labor	0.58 (0.58)	0.56 (1.00)	1.07 (0.90)	0.07 (0.08)
Facility managers	0.23 (0.22)	0.22 (0.39)	0.43 (0.35)	0.03 (0.03)
Procurement personnel	0.22 (0.29)	0.22 (0.42)	0.41 (0.43)	0.02 (0.03)
Chefs	0.13 (0.13)	0.12 (0.19)	0.23 (0.16)	0.02 (0.02)
Supporting health promotion materials	0.28 (0.50)	0.08 (0.21)	0.21 (0.36)	0.16 (0.42)
Salt consumption	10.32 (5.39)	3.47 (1.86)	6.59 (5.56)	7.20 (5.08)
MACE treatment cost	37.44 (4.68)	54.59 (6.82)	41.19 (5.15)	51.28 (6.41)
Changes in antihypertension medication cost	−7.19 (0.90)	−6.83 (0.86)	−6.99 (0.87)	−7.04 (0.88)
**For 2 y**				
Overall cost	76.27	102.22	89.66	88.94
Operating cost	20.86 (8.19)	7.38 (3.34)	14.77 (9.36)	13.62 (9.21)
Provincial-level subtotal	1.19 (1.19)	0.43 (0.68)	1.17 (1.13)	0.44 (0.80)
Labor	0.41 (0.36)	0.20 (0.29)	0.49 (0.38)	0.12 (0.14)
Transportation	0.78 (1.16)	0.22 (0.54)	0.68 (1.09)	0.32 (0.74)
Facility-level subtotal	19.68 (8.16)	6.95 (3.21)	13.60 (9.25)	13.18 (8.71)
Labor	0.69 (0.79)	0.74 (1.36)	1.51 (1.26)	0.07 (0.08)
Facility managers	0.26 (0.28)	0.28 (0.51)	0.51 (0.47)	0.03 (0.03)
Procurement personnel	0.28 (0.43)	0.31 (0.61)	0.71 (0.64)	0.02 (0.03)
Chefs	0.15 (0.15)	0.15 (0.24)	0.28 (0.21)	0.02 (0.02)
Supporting health promotion materials	0.33 (0.63)	0.08 (0.21)	0.21 (0.36)	0.20 (0.59)
Salt consumption	18.66 (8.16)	6.14 (2.94)	11.89 (9.00)	12.91 (8.68)
MACE treatment cost	72.88 (9.11)	111.18 (13.90)	91.78 (11.47)	92.26 (11.53)
Changes in antihypertension medication cost	−17.48 (2.18)	−16.34 (2.04)	−16.89 (2.11)	−16.93 (2.12)

Abbreviation: MACE, major adverse cardiovascular event.

^a^ Costs are reported in 2020 US dollars ($1 = ¥6.90).

Operating cost varied across intervention strategies. The salt substitute strategy had higher incremental operating costs than the restricted supply strategy (1 year, $7.74 vs $1.03; 2 years, $13.48 vs $1.16). For the salt substitute strategy, incremental operating costs were primarily incurred with the cost of salt substitute, which was higher than that of regular salt by $1.38/kg. For the progressive restriction of supply, incremental operating costs were mainly from the increased facility-level labor cost (1 year, $1.00; 2 years, $1.44). The second year showed lower overall incremental operating costs than the first year (salt substitute, $5.74 vs $7.74; restricted supply, $0.12 vs $1.03). MACE treatment costs accounted for most of the total costs at 2 years, which were lower with salt substitute compared with regular salt (mean [SD], $72.88 [$9.11] vs $111.18 [$13.90]), but did not differ by restricted vs usual supply at 2 years (mean [SD], $91.78 [$11.47] vs $92.26 [$11.53]) (Table 1; eTable 4 in Supplement 2). Intergroup differences in antihypertension medication cost were not significant (eTable 5 in Supplement 2).

### Cost-Effectiveness Analysis

Health outcomes and cost-effectiveness are presented separately for each intervention strategy (Table 2; eTable 6 in Supplement 2). Comparing participants receiving salt substitute vs regular salt at 1 and 2 years, the salt substitute group showed incremental effectiveness in reductions of mean SBP by 7.41 mm Hg (95% CI, 3.29-11.53 mm Hg) and 7.14 mm Hg (95% CI, 3.79-10.48 mm Hg), hypertension prevalence by 5.52 (95% CI, 0.71-10.33) percentage points and 5.09 (95% CI, 0.37-9.80) percentage points, cumulative MACE incidence by 0.84 (95% CI, −0.71 to 2.39) percentage points and 2.27 (95% CI, 0.09-4.45) percentage points (corresponding to a hazard ratio of 0.60 [95% CI, 0.38-0.96]^17^), and cumulative cardiovascular mortality by 0.74 (95% CI, −0.51 to 1.99) percentage points and 1.14 (95% CI, −0.57 to 2.85) percentage points (corresponding to a hazard ratio of 0.64 [95% CI, 0.44-0.92]^17^), although some differences were not significant. Comparison of the restricted supply group with the usual supply group did not show any significant incremental effectiveness. Accordingly, in the cost-effectiveness analysis at 2 years, salt substitution showed an incremental cost of −$25.95, indicating a cost-saving ICER. However, the restricted supply resulted in a dominated ICER at 2 years, as indicated by negative results for hypertension prevalence and MACE incidence despite a lack of statistical significance (eTable 7.1 in Supplement 2).

**Table 2.** Cost-Effectiveness Analysis at Different Stages of the Interventions

Indicator	Salt substitute vs regular salt		Restricted supply vs usual supply	
	For 1 y	For 2 y	For 1 y	For 2 y
**Systolic blood pressure**				
Incremental cost, US$	−9.77	−25.95	−9.00	0.71
Incremental effect (95% CI), mm Hg	7.41 (3.29 to 11.53)	7.14 (3.79 to 10.48)	0.21 (−4.46 to 4.88)	0.58 (−3.36 to 4.51)
Incremental cost-effectiveness ratio^a^	Cost-saving	Cost-saving	Cost-saving	1.23
**Hypertension prevalence**				
Incremental cost, US$	−9.77	−25.95	−9.00	0.71
Incremental effect (95% CI), percentage points	5.52 (0.71 to 10.33)	5.09 (0.37 to 9.80)	1.71 (−3.11 to 6.53)	−0.22 (−4.48 to 4.04)
Incremental cost-effectiveness ratio^a^	Cost-saving	Cost-saving	Cost-saving	Dominated
**MACE incidence (cumulative)**				
Incremental cost, US$	−9.77	−25.95	−9.00	0.71
Incremental effect (95% CI), percentage points	0.84 (−0.71 to 2.39)	2.27 (0.09 to 4.45)^b^	0.24 (−1.25 to 1.73)	−0.04 (−2.15 to 2.07)
Incremental cost-effectiveness ratio^a^	Cost-saving	Cost-saving	Cost-saving	Dominated
**Cardiovascular mortality (cumulative)**				
Incremental cost, US$	−9.77	−25.95	−9.00	0.71
Incremental effect (95% CI), percentage points	0.74 (−0.51 to 1.99)	1.14 (−0.57 to 2.85)^c^	0.18 (−1.04 to 1.41)	0.03 (−1.67 to 1.73)
Incremental cost-effectiveness ratio^a^	Cost-saving	Cost-saving	Cost-saving	2371.98

Abbreviation: MACE, major adverse cardiovascular event.

^a^ The term cost-saving refers to an intervention with negative incremental costs and positive incremental costs, indicating a highly cost-effective measure. The term dominated refers to an intervention with negative incremental effects, signifying an intervention that is not cost-effective.

^b^ An absolute difference in MACE incidence of 2.27 percentage points (95% CI, 0.09-4.45 percentage points) at 2 years corresponds to a 40% relative reduction in MACE risk in the salt substitute group compared with the regular salt group (corresponding to a hazard ratio of 0.60 [95% CI, 0.38-0.96]^17^).

^c^ An absolute difference in cardiovascular mortality of 1.14 percentage points (95% CI, −0.57 to 2.85 percentage points) at 2 years corresponds to a 36% relative reduction in cardiovascular mortality in the salt substitute group compared with the regular salt group (corresponding to a hazard ratio of 0.64 [95% CI, 0.44-0.92]^17^).

### Cost-Utility Analysis

The cost utility of the 2 intervention strategies was further investigated (Table 3; eTable 7.2 in Supplement 2). Compared with regular salt, salt substitute yielded an incremental QALY of 0.039 (95% CI, −0.100 to 0.178) and 0.093 (95% CI, −0.106 to 0.293) per individual at 1 and 2 years, respectively, which translates to approximately 14.24 and 34.09 additional days with full health. In contrast, the restricted supply showed negative incremental QALYs (mean, −0.0146 [95% CI, −0.1538 to 0.1246] at 1 year and −0.0006 [95% CI, −0.2005 to 0.1994] at 2 years), although the findings were not statistically significant. Therefore, salt substitution remained cost-saving in the cost-utility analysis, while restricted supply showed a dominated ICUR.

**Table 3.** Cost-Utility Analysis at Different Stages of Intervention

Indicator	Intervention group	Control group	Difference
**Salt substitute vs regular salt**			
For 1 y^a^			
Cost, US$	42.44	52.21	−9.77
Utility, QALYs (95% CI)^b^	−0.107 (−0.188 to −0.027)	−0.139 (−0.232 to −0.046)	0.039 (−0.100 to 0.178)
For 2 y^a^			
Cost, US$	76.27	102.22	−25.95
Utility, QALYs (95% CI)^b^	−0.268 (−0.375 to −0.161)	−0.345 (−0.468 to −0.223)	0.093 (−0.106 to 0.293)
**Restricted supply vs usual supply**			
For 1 y^c^			
Cost, US$	43.04	52.04	−9.00
Utility, QALYs (95% CI)^b^	−0.133 (−0.222 to −0.044)	−0.113 (−0.199 to −0.027)	−0.015 (−0.154 to 0.125)
For 2 y^c^			
Cost, US$	89.66	88.94	0.71
Utility, QALYs (95% CI)^b^	−0.314 (−0.430 to −0.199)	−0.298 (−0.413 to −0.183)	−0.001 (−0.201 to 0.199)

Abbreviation: QALY, quality-adjusted life year.

^a^ Indicates that this strategy resulted in a cost-saving incremental cost-utility ratio. The term cost-saving refers to an intervention with negative incremental costs and positive incremental costs, indicating a highly cost-effective measure.

^b^ The difference in utility was derived from a linear mixed model, with adjustment for baseline value and clustering effect at the facility level. The SD of QALY values was large because we considered the discounted lifetime QALY loss of cardiovascular deaths in the calculation of QALY change. In probabilistic sensitivity analyses, the plausibility range was assumed to be 25% above or below the base value.

^c^ Indicates that this strategy resulted in a dominated incremental cost-utility ratio. The term dominated refers to an intervention with negative incremental effects, signifying an intervention that is not cost-effective.

Sensitivity analyses showed similar results (Table 4; Figure; eTable 7.3 and eFigure 2 in Supplement 2). The base case analysis produced a cost-saving outcome that was robust when some key parameters were varied, except for increasing the price of salt substitute to the highest market price (ICUR, $354.22 per QALY), or excluding MACE treatment cost (ICUR, $132.20 per QALY). Within the DECIDE-Salt context, salt substitution is estimated to remain cost-effective until the price of salt substitute reaches $164.20/kg (Table 4). In the probabilistic sensitivity analysis, salt substitution had a 99% probability of being cost-effective vs the gross domestic product per capita (Figure).

**Table 4.** One-Way Sensitivity Analysis of the Salt Substitute Intervention at 2 Years

Scenario	Incremental cost, US$	Incremental effectiveness, QALYs	Incremental cost-utility ratio^a^
Base case	−25.95	0.0934	Cost-saving
Discount rate			
0%	−25.95	0.1178	Cost-saving
8%	−25.95	0.0814	Cost-saving
Operating cost composition			
Only facility-level costs considered	−26.71	0.0934	Cost-saving
Only salt cost considered	−26.91	0.0934	Cost-saving
Cost of substitute salt (base case price: $2.03/kg)			
At the lowest market price ($0.94/kg)	−32.65	0.0934	Cost-saving
At the highest market price ($11.59/kg)	33.08	0.0934	354.22
MACE treatment cost			
Cost not considered	12.35	0.0934	132.20
20% Reduction	−18.29	0.0934	Cost-saving
20% Increase	−33.60	0.0934	Cost-saving
Antihypertension medication cost			
Cost not considered	−24.81	0.0934	Cost-saving
20% Reduction	−25.72	0.0934	Cost-saving
20% Increase	−26.17	0.0934	Cost-saving
QALY change			
20% Reduction	−25.95	0.0747	Cost-saving
20% Increase	−25.95	0.1121	Cost-saving

Abbreviations: MACE, major adverse cardiovascular event; QALY, quality-adjusted life year.

^a^ The term cost-saving refers to an intervention with negative incremental costs and positive incremental costs, indicating a highly cost-effective measure.

**Figure.**
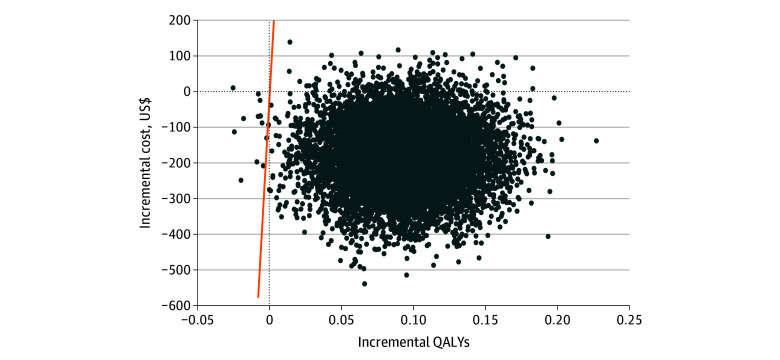
Probabilistic Sensitivity Analysis of the Salt Substitute Intervention at 2 Years Using Monte Carlo Simulation The orange line indicates the willingness-to-pay threshold, which is US $10 435 per quality-adjusted life-year (QALY) gained.

### Estimated Benefits and Costs

Universal use of salt substitute in eldercare facilities was estimated to avert 48 101 MACEs and 107 857 hypertension cases and gain 197 915 QALYs in the first 2 years nationwide compared with no salt substitution (eTable 8 in Supplement 2). The estimated 2-year cost savings was $54 982 278, including a saved antihypertension medication cost of $2 407 675, a saved MACE treatment cost of $81 145 171, and an operating cost of $28 570 569.

## Discussion

This within-trial economic evaluation of the DECIDE-Salt study shows for the first time, to our knowledge, considerable potential for cost-saving results of salt substitution among residents of eldercare facilities in China. The comparison of 2 feasible and scalable sodium reduction strategies revealed that, although the salt substitute strategy had higher incremental operating costs than the restricted supply strategy (1 year, $7.74 vs $1.03; 2 years, $13.48 vs $1.16), the salt substitute operating costs were offset by substantial savings in health care costs, resulting in robust cost savings. If salt substitution were rolled out to all eldercare facilities in China, a total of 48 101 MACEs, 107 857 hypertension cases, 197 915 QALYs lost, and a cost of $54 982 278 are estimated to be averted in 2 years.

The DECIDE-Salt trial examined 2 strategies to reduce sodium intake in a 1:1:1:1 factorial design. Facilities assigned to salt substitution spent more on salt procurement owing to the higher price of salt substitute. Those assigned to salt supply restriction spent less on salt procurement but required extra labor costs to achieve a progressive salt reduction. Although the restricted supply was less costly, priority should be given to the salt substitute strategy due to its substantial incremental health benefits. In contrast, efforts to restrict the supply of salt were unsuccessful. The underlying reasons for the unsuccessful efforts to progressively reduce salt supply could be very complex and have been illustrated in a previous study regarding individual behaviors and the progressive nature of the intervention.^17^

From a societal perspective, operating costs of the salt substitution strategy were offset by substantial savings in preventing MACEs, resulting in cost savings that offer a compelling case for the Chinese government and other countries to support a salt substitute program for eldercare facility residents. Saved costs derived mainly from averted treatment costs for MACE hospitalization, similar to results of the SSaSS that showed salt substitute was protective against stroke events, with a lower total cost compared with regular salt.^12^ The sensitivity analyses also showed robustness of the base case results. Subsequent analysis indicated that within the DECIDE-Salt context, salt substitution would remain cost-effective until the price of salt substitute reached $164.20/kg (an approximate 14-fold increase over the present highest market price [$11.59/kg] and 81-fold increase over the prevailing price of salt substitute in China [$2.03/kg]).

Previous economic evaluation studies, modeled or within trial, primarily focused on the population residing independently in the community,^12,13,14,15,16^ while the cost-effectiveness of facility-level salt substitution interventions alongside randomized trials remains unknown. Compared with older adults living independently in the community, those residing in eldercare facilities are often unable to live independently and require continuous medical care.^29^ As sense of taste tends to deteriorate with age, older adults may have a limited ability to self-regulate sodium intake. In residential eldercare facilities, facility kitchens prepare standardized meals, making it easier to conduct sodium reduction interventions by controlling salt procurement and supply channels, as well as providing training for facility staff and residents. Even if intervention costs and outcomes may vary substantially across settings,^12,30^ the intervention’s cost-effectiveness has been validated in our collective older adult population.

For this study, the collective living setting enabled an adequate implementation of sodium intake reduction interventions and completeness in follow-up visits, with better cost-effectiveness compared with prior community-based evidence. For instance, our findings from DECIDE-Salt extend those of SSaSS to a broader and older population with and without health conditions in residential care facilities and show an approximately double benefit for blood pressure and greater protection against MACEs.^11^ Although the SSaSS reported similar cost-saving results, the price of salt substitute at which the intervention became cost-effective was much lower than that reported in this study ($17.39 vs $164.20/kg).^12^ The DECIDE-Salt results showed clear health benefits and cost savings of salt substitute compared with regular salt, in line with the favorable findings of SSaSS and other modeled cost-effectiveness studies.^12,14,15,31^ Moreover, implementing salt substitution in a collective living setting where residents have limited control over meals and seasonings may maximize the cost-effectiveness of the intervention. Therefore, the DECIDE-Salt findings provide additional evidence to support the cost-effectiveness of salt substitute in eldercare facilities, with substantial reductions in disease burden and associated costs.

As estimated, if salt substitution were rolled out to all eldercare facilities in China, conservatively, there would be 48 101 MACEs averted, 197 915 QALYs gained, and $54 982 278 saved over the first 2 years of the intervention among 2.1 million older adults. As a reference, a previous modeling study estimated that nationwide implementation of salt substitution could avert 743 000 nonfatal cardiovascular events and 461 000 cardiovascular deaths annually among Chinese adults.^7^ Therefore, the within-facility intervention may generate more substantial reductions in cardiovascular disease burden and health care costs for a given population size compared with community-based interventions. Moreover, a lower incremental operating cost was observed in the second year compared with the first year ($5.74 vs $7.74), indicating diminishing marginal cost in favor of the long-term sustainability of this intervention. In light of China’s aging population and burden of noncommunicable diseases, nationwide implementation of the low-cost salt substitute intervention could be a crucial public health strategy. It is recommended for the government to provide salt substitute for free, or at least with policy incentives, to eldercare facilities. In addition, as a whole-population strategy, future research could examine the cost-effectiveness of salt substitution for the general population, as well as the feasibility and acceptability of implementing this intervention on a large scale.

### Limitations

Several limitations should be mentioned. First, hospitalization costs of MACEs were not collected as part of the trial follow-up but extracted from external sources and weighted based on the consequences observed in the trial. We performed robust sensitivity analyses to account for the lack of confidence in this estimate and found that variations by 20% would not affect the cost-saving conclusion; the intervention remained cost-effective even if we did not consider MACE treatment cost. Second, due to a lack of reliable data from the literature, this study did not involve direct nonmedical and indirect or sequelae costs for MACE hospitalization cases.^32^ Salt substitution may also save costs in cardiovascular outpatient visits, but we did not consider such costs owing to the difficulties in reliable measurement and their relatively minor contribution to the total treatment cost.^12,33^ Third, the benefits of salt substitution might be compromised by an increase in the frequency of hyperkalemia and hyponatremia.^34^ We did not investigate these conditions as none of the study participants were hospitalized for or clinically diagnosed with them. Although biochemical hyperkalemia risk was increased by salt substitution, no association with adverse clinical outcomes was reported in the DECIDE-Salt main results.^17^ Fourth, the incremental utility costs with wide CIs spanning 0 cannot be considered significant since we considered the lifetime QALY loss of cardiovascular deaths in the calculation. Although sensitivity analyses with varied QALYs showed robustness, the results should be interpreted cautiously. Fifth, the simplification of assumptions made in the nationwide estimation may have overlooked the heterogeneity that exists within the target population, leading to a lack of precision in the estimation of health benefits and costs.

## Conclusions

This study provides evidence on the cost-effectiveness of sodium reduction interventions from a cluster randomized clinical trial in eldercare facilities, supporting the promotion of salt substitute as an effective and cost-saving strategy against clinical events. The substantial health benefits and savings in preventing MACEs and the moderate operating costs offer strong evidence to support the Chinese government and other countries in planning or implementing sodium intake reduction and salt substitute campaigns for older adults in collective living settings.

## Notes

Supplement 1.
Trial Protocol

Supplement 2. **eFigure 1.** Flow Chart **eTable 1.** Questionnaires to Obtain Facility-Level and Provincial-Level Cost Indicators at Different Stages of Intervention **eTable 2.** Baseline Characteristics of Study Participants by Randomized Comparisons (n = 1612) **eTable 3.** Comparisons in Baseline Characteristics Between Participants Analyzed and Not Analyzed for the Assessment of Effect on Blood Pressure Among 1612 Eligible Participants **eTable 4.** Estimated MACE Treatment Cost for Each Participant at Different Stages of Intervention **eTable 5.** Estimated Changes in Antihypertension Medication Cost for Each Participant at Different Stages of Intervention **eTable 6.** Health Indicators for Study Participants at Different Stages of Intervention **eTable 7.** Economic Evaluation Results With ICER and ICUR Values **eFigure 2.** Probabilistic Sensitivity Analysis of the Restricted Supply Intervention at 2 Years Using Monte Carlo Simulation **eTable 8.** Projected Health Benefits and Costs When Salt Substitute Is Rolled Up Through All Eldercare Facilities in China

Supplement 3.
Nonauthor Collaborators

Supplement 4.
Data Sharing Statement
