# Supplementary material for: Cost-Effectiveness of Salt Substitute and Salt Supply Restriction in Eldercare Facilities: The DECIDE-Salt Cluster Randomized Clinical Trial
Source: JAMA Netw Open. 2024 Feb 12;7(2):e2355564. doi: 10.1001/jamanetworkopen.2023.55564 (PMC10862151; doi:10.1001/jamanetworkopen.2023.55564)
Supplement: Supplement 1. — Trial Protocol [file jamanetwopen-e2355564-s001.pdf]

Supplement 1

This appendix contains the following items:

1. Study protocols and amendments

    1.1 Original protocol (Ver 1.1, the first version submitted for IRB initial review)

    1.2 Final protocol (Ver 1.4)

    1.3 Summary of changes

2. Statistical analysis plan with summary of changes made from the published protocol

Evaluating the Efficacy, Safety, and Cost-Effectiveness of Using a Salt Substitute (SS) and Stepwise Salt Supply Control (SSSC) on Lowering the Blood Pressure of the Elderly in Nursing Homes

Research Organization	Peking University Clinical Research Institute
Principal Investigator	Yangfeng WU
Date of Writing	19-Sep-2017
Version Number	V1.1

CONTENT

Protocol Approval Page 1

Protocol Synopsis 2

1.Background 4

2.Trial Purpose 6

2.1 Primary Purpose 6

2.2 Secondary Purpose 7

3.Trial Design 7

4.Participants 8

4.1 Inclusion and Exclusion Criteria of Nursing Homes 8

4.2 Inclusion and Exclusion Criteria of Individual Elderly 9

5.Trial process and data collection 9

5.1 Preparation Period 9

5.2 Baseline Assessments 9

5.3 Randomization 11

5.4 Intervention Scheme 12

5.5 Follow-up 18

6.Outcome Measure 21

6.1 Primary Outcome 21

6.2 Secondary Outcome 21

6.3 Compliance evaluation 22

7. Safety Considerations 22

7.1 Risk-Based Safety Monitoring and Management Plan 22

7.2 Adverse Events 23

7.3 Other Possible Trial-Related Adverse Reactions 24

7.4 Serious Adverse Events 24

7.5 Data Safty Monitoring Board 25

8. Data Management 25

8.1 Data Management Center 25

8.2 Case Report Form Design and Electronic Database Construction 25

8.3 Data Management Plan and Data Validation Plan 26

8.4 Data Entry and Data Audit 27

8.5 Database Locking and Data Verification 27

9. Statistical Analysis 27

10.Sample Size Calculation 29

11. Quality Control 30

11.1 Modifications to the Protocol 30

11.2 Training 31

11.3 Monitoring 31

11.4 Quality Control for Data 32

12.Subject Protection 32

12.1 Ethics Review 32

12.2 Informed Consent 33

12.3 Confidentiality and Privacy 33

13.Trial Management 34

14. Storage of Research Documents and Records 36

15. References 37

Protocol Approval Page

I agree with this protocol (Version Number: V1.1, Version Date: 19/09/2017) and will conduct this trial in accordance with the Declaration of Helsinki as well as other relevant laws and regulations.

Peking University Clinical Research Institute

Signature of Principal Investigator:

Name of Principal Investigator (Printed): Yangfeng WU

Date:19-Sep-2017

Protocol Synopsis

Trial Title Evaluating the Efficacy, Safety, and Cost-Effectiveness of Using a Salt Substitute (SS) and Stepwise Salt Supply Control (SSSC) on Lowering the Blood Pressure of the elderly in Nursing Homes

Trial Purpose

The main purpose of the study is to determine the efficacy of using a salt substitute (SS) and stepwise salt supply control (SSSC) on lowering the blood pressure of the older adult living in senior residential facilities . At the same time, the research aims to observe the compliance and safety of the two intervention strategies, as well as evaluate their cost-effectiveness.

Trial Design

This is a multi-center, is a 2-year 2 × 2 factorial, cluster-randomized controlled trial. Senior residential facilities (geracomiums/old folk's homes) are randomized and used to test two salt reduction strategies, namely, 1) introduction of low-sodium salt substitute (SS) compared to continued use of regular salt.; and 2) Manage and control the salt procurement and supply channels of institutional kitchens a,which will be achieved together with health education and health promotion for chefs. This will be referred to as the "stepwise salt supply control"

36 eligible senior residential facilities in Changzhi, Xi'an and Hohhot will be selected and assigned into one of the following four intervention groups using stratified randomization: 1) SS + SSSC; 2) SS only; 3) SSSC only; and 4) No SS and no SSSC (control). The intervention will be last for 2 years. Follow-ups will be conducted at 6, 12, 18, and 24 months after the intervention.

Sample Size

A total of 36 senior residential facilities and at least 30 members from each senior residential facilities for a total of at least 1080 participants will be included in our study.

Participant Selection

Inclusion and Exclusion Criteria of Nursing Homes

Inclusion Criteria:

1) Have more than 30 elderly people staying there for a prolonged period.

2) Have a record of the elderlies' entry and exit from the nursing home, where they went, the reason they left, and the duration they left for.

3) Have a person responsible for the purchase of food and condiments, as well as an area designated for the storage and safekeeping of food.

4) Responsible for preparing the food consumed by the elderly and must not purchase processed food from outside the nursing home more than once a week.

5) Must be willing to accept the intervention to reduce salt usage and have a high degree of cooperation.

6) Signed the partnership agreement.

Exclusion Criteria:

1) Have participated in any salt reduction or other intervention trials in the past or currently.

Inclusion and Exclusion Criteria of Individual Elderly

Inclusion Criteria:

1) Living in the nursing home permanently or expectedly for the coming two years and do not spend more than a month outside of the nursing home.

2) Life expectancy over six months.

3) Age less than 55 years.

Exclusion Criteria:

1) with no data from all the three key measurements: blood pressure, blood sample and 24-hour urine will be excluded.

2) Clinically confirmed hyperkalemia.

Research Duration: 4 years

1.Background

Cardiovascular diseases are currently the leading cause of death in our country, accounting for 42.61% and 45.01%[1] of deaths in cities and rural areas respectively. With the changes in the lifestyles of the Chinese people, as well as the acceleration of the aging population and urbanization, the absolute number of deaths from cardiovascular diseases has been rapidly increasing, up 46% in 2013 as compared to 1990. Amongst those, there has been a 90.9% increase in deaths due to ischemic heart diseases and a 47.7% increase in deaths from cerebrovascular diseases. At the same time, the prevalence of cardiovascular diseases have also been rising steadily over the years. According to estimates, 290 million people have developed cardiovascular diseases, with 13 million developing strokes and 11 million developing coronary heart diseases. Faced with the massive population that have developed the diseases and the continually high death rate, the disease burden of cardiovascular diseases in our country has been increasing over time. It has become an important public health issue, and the prevention and control for it must not be delayed. Hypertension is an important risk factor for cardiovascular diseases. Globally, hypertension is related to 54% of strokes and 47% of ischemic heart attacks[2]. In our country, the prevalence of hypertension is also increasing each year. Based on the latest investigation results on a sample survey for hypertension involving 500000 people in the country, the prevalence rate of hypertension in our country is 23%, with 240 million people having hypertension. Controlling blood pressure has become an important precautionary measure as it will significantly reduce the risk of developing and dying from cardiovascular diseases.

Many researches have shown that an important factor of food consumption on blood pressure is the intake of salt (sodium). Reducing the intake of salt or replacing regular salt with a salt substitute can significantly reduce the blood pressure of patients with hypertension or high-risk individuals, preventing the development of cardiovascular diseases and lowering the number of deaths in the population[3]. The World Health Organization has even regarded reducing salt intake as one of the three "Best Practices" for the prevention of chronic diseases[4]. Despite that, our country still has a high-salt diet. The 2012 National Nutrition Survey revealed that the population still consumes a high amount of sodium, equaling 14.5 g/d when converted to table salt, which is

far higher than the World Health Organization's recommended daily intake of not more than 5 g per day for adults[5]. Thus, it is especially important that we develop and promote suitable and operable salt reduction intervention techniques amongst the high-risk population for cardiovascular diseases in our country.

The prevalence of hypertension is significantly increased amongst the elderly population, which greatly increases the risk of the development and death from cardiovascular diseases. Also, with the gradual deterioration of an individual's sense of taste with age, the elderly will also become less able to self-regulate their intake of sodium. Thus, the elderly population is both a high-risk group for cardiovascular diseases and a key population for the salt reduction strategies.

There have already been many large-scale randomized controlled trials showed that salt substitutes can lower the blood pressure of people with hypertension[6-9]. However, the long-term efficacy of salt substitutes on lowering the blood pressure of the average elderly population remains unclear. Furthermore, the main components of salt substitutes include sodium chloride and potassium chloride. Compared to regular salt, consuming the salt substitute that contains less sodium and more potassium can increase the intake of potassium, which might potentially affect the urine potassium, blood potassium and kidney function. Even though the Food and Drug Administration has deemed salt substitutes containing potassium chloride as safe in 1983 and reported that no adverse reactions were observed in the consumption of salt substitutes amongst the people with normal kidney function, the safety of these intervention strategies on the elderly population still lack ample evidence. Further research is required to obtain the relevant data, so as to build a solid foundation for the large-scale promotion of salt reduction strategies in the future[10].

Currently, salt substitutes on the market contain no more than 30% less sodium chloride than regular salt. Judging from the average salt consumption level of our population, merely using salt substitutes cannot push the Chinese diet toward the recommended salt intake in Chinese Dietary Guidelines. The centralized provision of meals for the elderly in nursing homes makes the kitchen of these nursing homes an optimal trial site for the salt reduction strategies. A research project targeted at reducing the salt intake of the collective elderly population in the US adopted a gradual

and acceptable approach to salt reduction. The intervention strategies used were targeted at the supply of meals for the elderly and the kitchen of the nursing homes. Specifically, the intervention strategies like replacing high-sodium foods with low-sodium foods, improving recipes, and changing the ways cooks prepared the foods were adopted. Ultimately, the sodium content of the meals supplied to the elderly decreased by 9.76% in the first year and 14.17% in the second year[11].

In our country, a large number of elderly live mainly in government-run geracomiums and publicly or privately-run nursing homes. Even though these two kinds of nursing homes are differentiated by whether or not a fee has to be paid for their use, both organizations provide meals for the elderly from their kitchens. Building on the intervention strategies used in past literature as well as the unique features of the facilities that house the elderly in our country, our research group has developed intervention strategies to gradually decrease salt usage and create a stepwise salt supply control. This will be done through controlling the elderly care facilities' purchase of salt and its supply channel and complemented by health education and health promotion for the cooks and the elderly staying in the facilities. The feasibility and efficacy of these salt reduction strategies on reducing the blood pressure of the elderly in Nursing Homes need to be evaluated. If proven to be effective and cost-effective, these salt reduction strategies together could become a new and important measure for the prevention and control of hypertension and cardiovascular disease in our country. It might be possible to lower the community's salt intake to less than 6 grams a day as recommended by the Chinese Nutrition Society, should the combination of these two strategies develop a larger effect. This is an important question that must be answered scientifically.

Thus, this study will evaluate the efficacy and safety of using salt substitutes and a stepwise salt supply control intervention on lowering the risk of cardiovascular diseases in the elderly in Nursing Homes , so as to build a scientific foundation for the large-scale promotion of salt reduction strategies.

2.Trial Purpose

2.1 Primary Purpose

To compare the effects of using only salt substitutes, only the stepwise salt supply control, and using both intervention strategies in tandem on reducing the blood pressure of the elderly in

Nursing Homes against a control group.

2.2 Secondary Purpose

- To investigate if salt substitutes would increase the prevalence rate of adverse reactions such as hyperkalemia, as well as whether the kidney function would affect that effect amongst the elderly in Nursing Homes by comparing the results against a control group.
- To investigate if the salt reduction strategies would decrease the development of cardiovascular diseases and the resulting deaths by comparing the results against a control group.
- To evaluate the degree of compliance and cost-effectiveness of adopting salt substitutes and the stepwise salt supply control in the elderly in Nursing Homes .
- To explore the efficacy of the intervention strategies on proteinuria in the elderly with damaged kidney function and whether the kidney function influences that effect.
- To evaluate the effect of the intervention strategies on fundus atherosclerosis.

3.Trial Design

This is a multi-center, is a 2-year 2 × 2 factorial, cluster-randomized controlled trial. Senior residential facilities (geracomiums/old folk's homes) are randomized and used to test two salt reduction strategies, namely, 1) introduction of low-sodium salt substitute (SS) compared to continued use of regular salt.; and 2) Manage and control the salt procurement and supply channels of institutional kitchens a,which will be achieved together with health education and health promotion for chefs. This will be referred to as the "stepwise salt supply control"

36 eligible senior residential facilities in Changzhi, Xi'an and Hohhot will be selected and assigned into one of the following four intervention groups using stratified randomization: 1) SS + SSSC; 2) SS only; 3) SSSC only; and 4) No SS and no SSSC (control). The intervention will be last for 2 years. Follow-ups will be conducted at 6, 12, 18, and 24 months after the intervention.

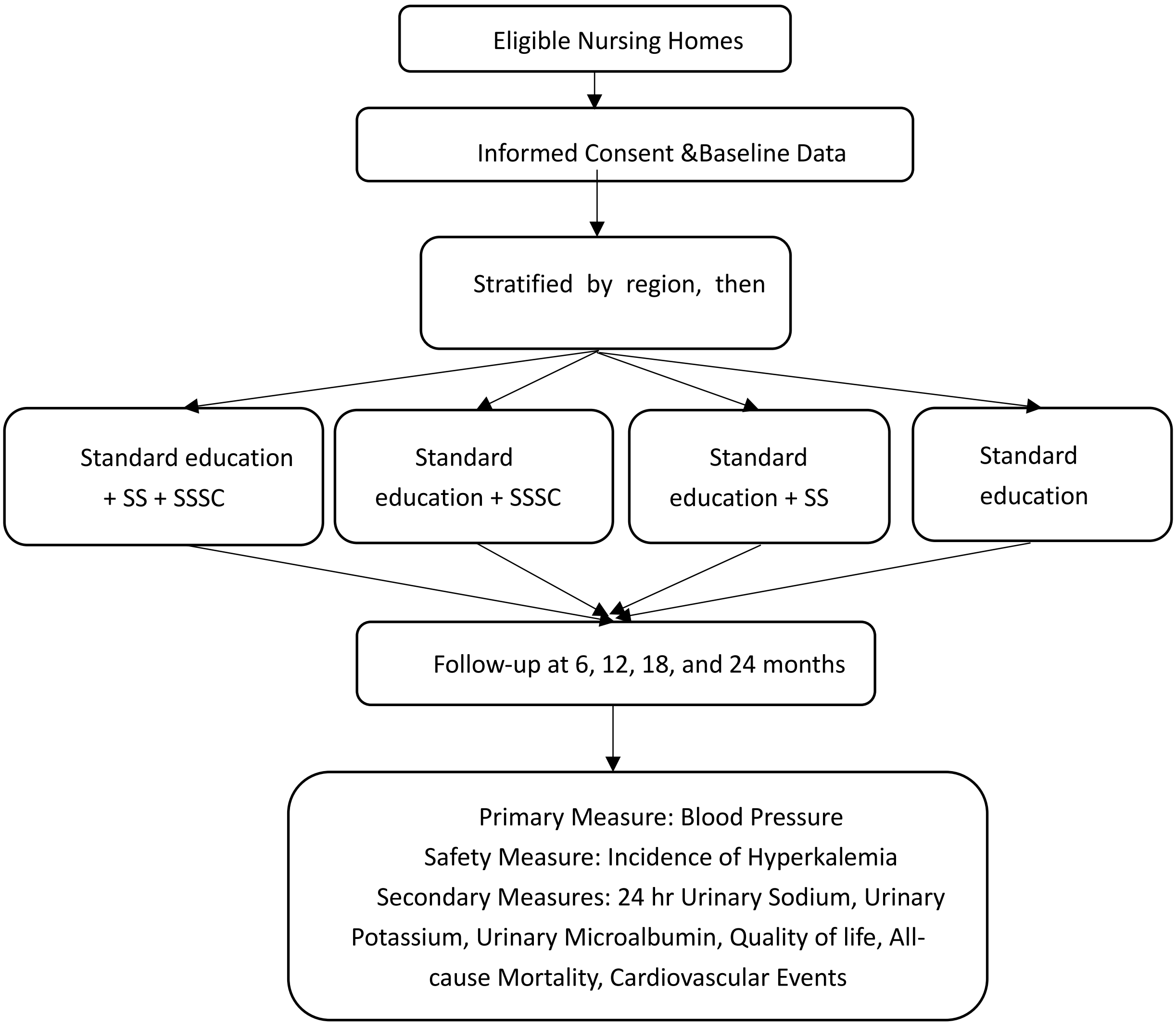

4.Participants

4.1 Inclusion and Exclusion Criteria of Nursing Homes

Inclusion Criteria:

1) Have more than 30 elderly people staying there for a prolonged period.

2) Have a record of the elderlies' entry and exit from the nursing home, where they went, the reason they left, and the duration they left for.

3) Have a person responsible for the purchase of food and condiments, as well as an area designated for the storage and safekeeping of food.

4) Responsible for preparing the food consumed by the elderly and must not purchase processed food from outside the nursing home more than once a week.

5) Must be willing to accept the intervention to reduce salt usage and have a high degree of cooperation.

6) Signed the partnership agreement.

Exclusion Criteria:

1) Have participated in any salt reduction or other intervention trials in the past or currently.

4.2 Inclusion and Exclusion Criteria of Individual Elderly

Inclusion Criteria:

1) Living in the nursing home permanently or expectedly for the coming two years and do not spend more than a month outside of the nursing home.

2) Life expectancy over six months.

3) Age less than 55 years.

Exclusion Criteria:

1） with no data from all the three key measurements: blood pressure, blood sample and 24-hour urine will be excluded.

2） Clinically confirmed hyperkalemia.

5.Trial process and data collection

5.1 Preparation Period

Relevant organizations in the various regions will be responsible for the selection of eligible nursing homes in their region based on the inclusion criteria. After the nursing homes confirm their participation and sign the partnership agreement, the relevant organizations will head down to the nursing homes and select eligible elderly based on the inclusion and exclusion criteria.

5.2 Baseline Assessments

Baseline assessments will be arranged and conducted after the signing of the partnership agreement and informed consent forms.

Investigations on the nursing homes include:

- Accommodation cost of the elderly
- Types, quantity, and frequency of condiments purchased as well as the frequency and quantity of processed food and foods with high salt content purchased
- Usage of the salt and condiments purchased: Cooking, homemade preserved vegetables or fermented vegetables, feeding livestock

Investigations on the elderly include:

-Contents:

Questionnaire: General Demographic Data: Gender, age, ethnic group, education level, marital status, and medical insurance of the participant.

Lifestyle Information: Smoking, alcohol consumption, level of physical exercise, frequency of fruit and vegetable consumption.

Level of Satisfaction in Meals: How much they liked the meals provided by the nursing home, self-assessment of the level of saltiness in meals.

Disease History: Whether or not they are suffering from hypertension, diabetes, stroke, coronary heart disease, kidney disease, cancer, or COPD. As well as other disease histories and whether they are receiving treatment.

Information on Antihypertensive Drugs: Type, specification, and frequency of consumption of antihypertensive drugs.

Occurrence of an Endpoint Event: Whether or not there is all-cause mortality or cardiovascular events (coronary heart disease, congestive heart failure, heart arrhythmia, and stroke).

Health Status: Will be evaluated using the EuroQol Five Dimensions Questionnaire (EQ-5D).

Physical Examination: Weight, height, blood pressure, heart rate.

Laboratory Examination: Serum electrolytes (blood potassium), blood sugar and four items of blood lipid, kidney function (serum creatinine), and blood routine. Levels of 24-hr urinary sodium, urinary potassium, urinary creatinine, and quantitative microalbuminuria.

Eye Fundus Examination: Vision test and funduscopy.

-Methods:

- Questionnaire and Health Status: Professional investigators who have undergone standardized training will read through and check the nursing homes' travel records and medication information. The participants will be asked for the rest of the information. The information will be further verified through the New Rural Co-operative Medical System.
- Physical Examination: Professional investigators who have undergone standardized training will conduct the physical examination on the participants. See Annex 4 for specific details on the examination methods.
- Laboratory Examination: Participants will be required to empty their bladder and record the time in the morning. They will then be given a 24-hr urine bucket and urine cup to collect their urine over the next 24 hours. The urine collection will be stopped at the same time on the morning of the second day. The urine volume of the participants over 24 hours, left out situations of urine, and the start and end time of the collection will be recorded. After mixing the 24-hr urine sample thoroughly, 5ml of urine will be collected and stored at -20°C. 6.5ml of venous blood will be collected from participants on an empty stomach on the morning of their physical examinations. See Annex 5 for the specific requirements on blood sample collection, packaging, transportation, storage, and determination.
- Eye Fundus Examination: A Chinese standard long-distance visual chart will first be used to investigate the normal vision of the participants. Participants who are wearing glasses during the examination will be tested for their vision with glasses; participants who are not wearing glasses during the examination will be tested for their naked vision. Photographs of the participants' eye fundus were then taken using fundus cameras (TRC-NW Series (Topcon), CR-2 AF Model (Canon), etc.) in non-mydriatic conditions.

5.3 Randomization

The randomization process of this research will be conducted by the Peking University Clinical Research Institute. An independent statistician carried out the randomization after the baseline characteristics had been collected for study sites and individual participants. Nursing homes are

stratified by region before being randomly assigned to the experimental groups.

5.4 Intervention Scheme

Intervention Strategies

There are two main intervention strategies, namely salt substitute and stepwise salt supply control. All nursing homes will also receive general health education on how salt reduction can prevent hypertension and cardiovascular diseases.

1. General Health Education on Salt Reduction

This research will provide a general health education activity to all participating nursing homes (including nursing homes in the control group). The specific details are as follows:

Managers of all the nursing homes will be invited to the trial initiation meeting held at the the study center of the various regions. The aim, design, and requirements of the trial will be explained to them. This includes the health benefits and the existing conflicts that the international research community has on salt reduction and cardiovascular diseases.

At each facility, health education program on salt reduction will be delivered on the baseline survey initiating meeting (with the managers of nursing home, cooks, and all elderly living in the nursing home participated). Posters will also be put up. The main contents of the education program are: Basic health information (The dangers of consuming too much salt, the recommended amount of salt intake in China, and how to reduce salt intake, etc.).

2. Replacing Regular salt with a Salt Substitute

2.1 Salt Substitute management (supply and distribution)

Nursing homes that are allocated to the salt substitute group will use a standardized salt substitute product as assigned by our study. They will do a 100% replacement of the regular salt that they were using. This will be managed and implemented by an assigned professional from the site. Any personnel who are not involved in the trial cannot come into contact with the salt substitute used in this trial. The salt substitute to be used in this trial is a standardized low-sodium salt that contains potassium which can be found on the market. It will be tested by an independent third party to ensure that the product is qualified.

The salt substitute will be supplied by an assigned supplier. To increase the generalizability of

the salt substitute in the future, nursing homes will purchase it based on their local market prices. The research group will waive the difference in price between the salt substitute and regular salt. Suppliers have to ensure that they have a sufficient supply of the salt substitute during the intervention period, and there should not be a shortage and out of stock.

The salt substitute used in the trial must be supplied to the specific nursing homes in strict accordance to the trail requirements. The purchaser and cook of the nursing homes must not hand over or sell the salt substitute. They must not use the salt substitute for something else and most importantly, they must not let non-participants of this research consume the salt substitute.

The trained local investigators will audit the salt supply records once every three months to ensure that the nursing homes are using the salt substitute, so as to evaluate the compliance of the intervention.

2.2 Training on the Correct Usage of the Salt Substitute

This research will provide extra training on the correct usage of the salt substitute for the nursing homes in the SS group. The purchasers and managers of salt in these nursing homes will receive a standardized training and learn about the composition of the salt substitute, its effects, and potential side effects. The core pieces of information are "the salt substitute is not less salty but can lower blood pressure" and "the salt substitute is still a salt, so it's best to still cut down on it".

3. Kitchen-based SSSC program

3.1 Controlling Salt Supply with the Aim of Gradually Reducing Salt Usage in the Kitchens

Assigned personnel from the local study center will be responsible for the target setting and implementation of the salt supply control. This person and the person responsible for the salt substitute intervention strategy cannot be the same person.

➢ Management Target and Plan for Gradual Reduction of Cooking Salt Usage

The main strategy behind this intervention is to control the person purchasing salt for the nursing homes, so as to gradually reduce salt usage in the kitchen in a stepwise manner. The intervention strategy consists of 4 stages. The first stage is the preparatory stage, which lasts for a

month. The nursing homes will continue the amount of salt they normally use in their cooking and begin recording how much salt they use to be used as a baseline reference. The second stage consists of two steps, with every step lasting 3 months for a total of 6 months. At this stage, the salt reduction target for every step will be 10% from the nursing home's baseline salt usage. The third stage consists of four steps, with every step lasting 3 months for a total of 12 months. At this stage, the salt reduction target for every step will be 5% from the nursing home's baseline salt usage. The fourth stage is the maintenance stage. The amount of salt used in the nursing homes should now decrease to 60% of their baseline salt usage. This will not be lowered further until the end of the research. (As per diagram below)

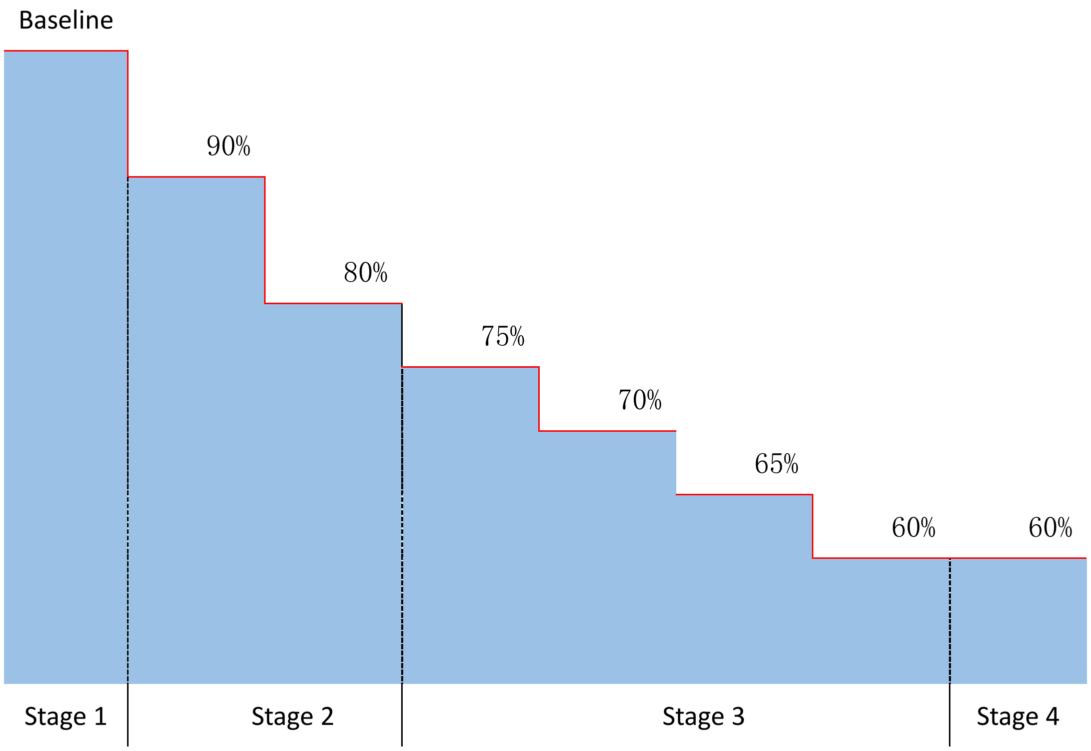

Kitchen-based stepwise salt supply control program.

➢ Management of Stepwise Salt Supply Control and evaluation

Nursing homes that have been assigned to the SSSC group will send their purchasers and cooks to receive standardized technical training. See Annex 2 (Stepwise Salt Supply Control Technical Training) for detailed contents of the training.

Through the training, purchasers will learn and understand the salt reduction targets throughout the various stages and the specific ways to control salt supply; and cooks will learn how

to weigh 5% and 10% of their ordinary salt usage. The two parties have to learn the reason for salt reduction and techniques to communicate with the elderly.

The person-in-charge from the sub-sites will audit the stock in and stock out records once every three months to evaluate if the goal for the stage has been met. This will be the basis for the decision on whether to activate the next stage of the intervention and intervention target. Nursing homes are considered to have met the target if the difference between their actual salt usage and the target salt usage is no more than ±30% and can move on to the next stage. A forum will also be held for the elderly to gather feedback and opinions. For nursing homes that fail to meet the target, a forum has to be held between the managers of the nursing homes, personnel controlling the supply of salt, cooks, and an elderly representative to identify challenges and resolve them. These nursing homes will also keep their original salt reduction target for the next 3 months. They will only go on to the next target after they meet their current target. Should a nursing home fail to meet half of the expected target for 2 consecutive stages, the intervention strategy will end for the nursing home at their current salt usage level. Should a nursing home exceed the salt reduction target of their current stage, they will be requested to slow down their rate of salt reduction and comply with the planned target.

- Incentives

Results-based incentives for the purchasers and cooks will be set up in accordance with the salt reduction effects and progress of the nursing homes.

3.2 Maintaining a Stock in and Stock out Record for the Purchase of Salt, Condiments, and Foods with High Salt Content, as well as A Record for the Number of Diners per Meal

Nursing homes that are assigned to the SSSC group have to establish a stock in and stock out record for condiments and foods with high salt content, as well as a record for the number of diners per meal. They will also have to record this information truthfully on the standardized stock in and stock out record form and diner record form provided by the research group (see Annex 3).

Items that have to be recorded in the stock in form include:

- Table salt
- Condiments (e.g. soy sauce, chicken bouillon, MSG, fermented chili bean paste, chili paste, sweet bean sauce, etc.)
- Foods with high salt content (e.g. salted vegetables, pickled vegetables, preserved eggs, fermented vegetables, etc.)
- Processed foods (e.g. instant noodles, vermicelli, bread, sausages, etc.)

Purchasers have to keep a detailed record of the item type purchased, date, quantity, and number of items left on the purchase record form.

Salt and condiments have to be placed in locked storage rooms and managed by the purchasers. Cooks are not allowed to obtain these items on their own. If the condiments in the kitchen have been used up and needs to be replenished, the cook has to inform the manager about the type and amount of condiment needed. The purchaser will procure the required condiment from the storage room and replenish the stock in the kitchen. At the same time, the purchaser has to record the type, date, and quantity of the item that was taken out of the storage room in detail in the stock out form. Aside from that, a daily record of the number of diners in the nursing homes has to be kept (includes the elderly and staff).

The stock in, stock out, and number of diners will be recorded immediately after the randomization has been done and will end when the intervention is over.

3.3 Stepwise Salt Supply Control Plus Health Education

- An extra and targeted health education will be supplied to nursing homes assigned to the SSSC group. Its aim is to increase the degree of compliance of the members of the nursing homes to the intervention measures and reduce the potential obstacles and resistance. This event will be executed by the personnel responsible for the SSSC intervention strategy from the sub-sites.
- Health Education Target Audience. Includes managers of nursing homes, purchasers, cooks, as well as the elderly living in the nursing homes for a prolonged period and their caretakers.
- health education activity.

1) An SSSC initiation meeting will be held in every nursing home and all personnel stated

above will be invited. A standardized lesson will be conducted on the benefits of consuming less salt, the overall aim of the activity, etc;

2) A forum for the elderly will be held during the inspection once every 3 months to obtain feedback and opinions about the salt reduction intervention, as well as to launch the targeted health education;

3) Release health education publicity materials during appropriate times: Such as promotional posters, pamphlets, etc;

4) Along with technical training, engage in targeted health education with the managers, purchasers, and cooks of the nursing home. Muster an subjective initiative to launch various salt reduction strategies on their own and increase the level of awareness and thought for the elderly's health.

5.5 Follow-up

- Follow-up timings: 6, 12, 18, and 24 months after the randomization
  Follow-up content: See the Table below.
- Follow-up method:

Questionnaire: Professional investigators who have undergone standardized training will read through and check the travel records and medication information of the nursing homes, as well as question the participants. The information will be further verified through the New Rural Co-operative Medical System.

Physical examination: A physical examination will be conducted on participants. Laboratory Examination: Participants will be required to empty their bladder and record the time in the morning. They will then be given a 24-hr urine bucket and urine cup to collect their urine over the next 24 hours. The urine collection will be stopped at the same time on the morning of the second day. The urine volume of the participants over 24 hours, left out situations of urine, and the start and end time of the collection will be recorded. After mixing the 24-hr urine sample thoroughly, 5ml of urine will be collected using a catheter and stored at -20℃. 4ml of venous blood will be collected from participants on an empty stomach on the morning of their physical examinations.

Research Follow-up Form

Time / Items	Preparation Period	Baseline	Follow-up Period						Early Withdrawal
			Month 6	Month 12	Month 18	Month 24	At any time	Transferring to Another Hospital	
Signing of Informed Consent Form	√								
Confirmation of Inclusion and Exclusion Criteria	√								
Basic Information Page	√								
Demographic Data		√							
Lifestyle		√							
Satisfaction in Meals		√	√	√	√	√			√
History of Disease and Treatment		√							
EuroQol Five Dimensions		√		√		√			√

Questionnaire (EQ-5D)									
Height, Weight		√				√			√
Blood Pressure, Heart Rate		√	√	√	√	√			√
Blood Routine		√				√			√
Blood Sugar, Four Items of Blood Lipid		√				√			√
24hr Urine		√				√			√
Eye Fundus Examination		√				√			√
Antihypertensive Drug Information		√	√	√	√	√			√
Collection of Hospitalization Records		√						√	
Reports of Adverse Reactions							√		
CRF Signature Page						√			√

- Early Withdrawal from Trial

- Criteria for early withdrawal:
    1. Researchers determine that it is not suitable for the research to continue because of adverse events;
    2. Participants request to withdraw from the research;
    3. Going against the protocol and others.
- The reason for early withdrawal should be recorded for participants who withdraw from the trial after meeting the early withdrawal criteria. And the following examination items should be performed and recorded during the last visit to the best of the ability:
  – Laboratory examination: Blood routine, kidney function, blood sugar, blood lipid, 24-hr urine
  – Physical examination: Height, weight, blood pressure, heart rate
  – Information about antihypertensive drug consumption, satisfaction in meals
  – EuroQol Five Dimensions Questionnaire (EQ-5D)
  – Endpoint events, safety incidents
  – Monitoring and record of adverse events

6.Outcome Measure

6.1 Primary Outcome

The primary outcome for the study is the difference of SBP between baseline and at 24 months of intervention.

6.2 Secondary Outcome

1. The incidence of hyperkalemia : as the main safety evaluation index. According to the 2012 Kidney Disease Improving Global Outcomes (Kidney Disease Improving Global Outcomes), serum potassium> 5.5 mmol/L is diagnosed as hyperkalemia; continuous measurement of serum potassium> 5.5 mmol/L is persistent hyperkalemia. Otherwise, it is a single attack.
2. The change in 24-hour urinary sodium、potassium and microalbumin
3. The change in diastolic blood pressure
4. European Five-Dimensional Health Scale (EQ-5D) score
5. Endpoint events (death, cardiovascular events (coronary heart disease, congestive heart failure, arrhythmia, stroke))
6. Fundus Atherosclerosis

6.3 Compliance evaluation

- Proportion of nursing homes that 100% use salt substitute in the SS group
- The proportion of nursing homes that reduce their salt usage by more than 20% in the SSSC group
- Satisfaction level of the elderly in intervention groups

7. Safety Considerations

7.1 Risk-Based Safety Monitoring and Management Plan

- **All participants will be screened for hyperkalemia and its related risk factors during the baseline assessment and Month 12 follow-up.**

All participants enrolled in the trial will be tested for blood potassium and blood creatinine levels during the baseline investigation. Meanwhile, the information about the antihypertensive drugs (ACEI, ARB, potassium-sparing diuretics, etc.) taken by the participants will also be collected.

All participants enrolled in this trial will be tested once again for blood potassium and blood creatinine levels during the Month 12 follow-up.

- **Based on the results of the screening, any elderly who fit the clinical diagnostic criteria for hyperkalemia will be transferred to a local secondary hospital or above for further blood potassium and ECG testing. They will then be treated for hyperkalemia confirmed by the doctor. The elderly who are not diagnosed with hyperkalemia but fit any of the following conditions will be designated as the high risk group for monitoring.**

1. Participants with blood potassium >5.5mmol/L at baseline;
2. Participants with 15<=eGFR<30mL/min/1.73m^2 (Patients with Stage 4 chronic renal insufficiency) at baseline.

The high risk group for monitoring will receive a blood potassium test every 3 months. Participants who are clinically diagnosed with hyperkalemia will be transferred to a local secondary hospital or above for hyperkalemia treatment. Participants who are not diagnosed with hyperkalemia will continue to be monitored for blood potassium 3 months later. The monitoring can be stopped temporarily should the blood potassium level of the participants <=5.5mmol/L in two consecutive tests. Otherwise, they will continue to be tested for blood potassium once every 3 months.

- **Blood potassium monitoring plan for the SS intervention group:**

All elderly in the SS intervention group, aside from those who are confirmed to have hyperkalemia or are placed in the high risk group for monitoring during the baseline examination,

will go through blood potassium test during the third、fifth and eigthth week after intervention. Based on the screening results (clinical diagnosis of hyperkalemia or high risk group for monitoring), participants will undergo the relevant risk monitoring and treatment as mentioned above.

➢ **Clinical Diagnosis of Hyperkalemia and Handling Scheme (According to the 2012 clinical guidelines of the Kidney Disease Improving Global Outcomes)**

- Participant with a serum potassium of >5.5mmol/L:

Immediately notify the nursing home where the participant resides in. The nursing home should take the participant to the hospital for another blood potassium test and ECG examination.

- Should the doctor diagnose the participant sent to the hospital with hyperkalemia based on the blood potassium test and other symptoms, the participant will be recommended to immediately undergo treatment such as taking hydrochlorothiazide, sodium polystyrene sulfonate, or if necessary, undergo emergency intravenous infusion to lower potassium levels. If the participant is currently taking antihypertensive drugs like ACEI/ARB, he/she will be required to stop taking those medicines and switch to antihypertensive drugs such as calcium channel blockers, α-receptor blockers, and β-receptor blockers or αβ-receptor blockers. The participant should also be advised to pay attention to their diet to avoid fresh jujube, peach, banana, tomato, soy, tofu, corn, seafood, bacon and other potassium-rich fruits, vegetables, fish, shrimps, crabs, meats, and nuts. The hyperkalemic patient can return to the nursing home after recovery. A review will be done two weeks later. If the participant's blood potassium level is once again >5.5mmol/L and the participant is in the SS intervention group, then he/she will go through treatment to reduce potassium levels and be terminated from the research. Otherwise, the participant will continue to be referred for potassium-lowering treatment and continue to be monitored.

➢ **Early termination of research**

The Participants will be stopped from SS intervention if they presented with three consecutive serum potassium measurements >5.5mmol/L. The participants can discuss with the nursing home to be transferred to a nursing home that is not participating in the research. Alternatively, the cooks of the nursing home may prepare food using regular salt just for the participants.

7.2 Adverse Events

Adverse events are any adverse medical incidents that happen to the participant after participating in the research. It does not have to have a causal relationship with the intervention strategies.

Adverse events can be any of the following adverse and unexpected physical signs (including abnormal laboratory data), symptoms, or diseases. It does not have to be related to the intervention strategies.

7.3 Other Possible Trial-Related Adverse Reactions

Gastrointestinal adverse reactions: Participants complain about symptoms such as nausea, vomiting, and diarrhoea. Should these reactions occur, the nursing home should be notified to send the participants to the hospital for treatment. Get a doctor to diagnose if the symptoms are a result from the intervention strategies. If these adverse reactions are due to the intervention strategies of this research, then the participants must be stopped for the reseach. The participants can discuss with the nursing home to be transferred to a nursing home that is not participating in the research. Alternatively, the cooks of the nursing home may prepare food using regular salt just for the participants.

Changes in blood pressure: The blood pressure of participants who regularly take antihypertensive drugs must be routinely monitored during the research period. If there is a significant decrease in their blood pressure, notify the nursing home and send them to the hospital. Get a doctor to gradually adjust the prescription of the drugs to prevent any fainting incidents that might result from an overly low blood pressure. After the research has ended, conduct a follow-up on the participants who regularly take antihypertensive drugs and monitor the changes in their blood pressure after the salt reduction strategies have ended. The prescription of their drugs should be adjusted to prevent any sudden increase in their blood pressure.

7.4 Serious Adverse Events

A serious adverse event (SAE) is defined as having any of the following outcomes resulting from the intervention:

- Threat to life
- Requiring hospitalization
- Permanent or significant disability and dysfunction

Should an SAE occur during the course of the research, researchers must adopt the suitable treatment measure on the participant immediately and end the trial.

Should an SAE occur, whether or not it has to do with the intervention strategiesresearchers have to record the details following content and report it to the Ethics Committee after the research is over: Time of occurrence, end time, measures adopted, relationship with the investigational drugs, etc.

Should any adverse events occur, researchers have to record the following on the CRF: Time

of occurence, level of severity, end time, measures adopted, and outcome.

The severity of adverse events can be classified into the following 3 levels:

- Mild: Light symptoms and physical signs. Usually a one-off event that does not require treatment and will not affect daily activities. Symptoms would automatically be relieved with rest;
- Moderate: Symptoms and physical signs persist for a longer time. Slightly affects daily activities. Will recover with simple treatment;
- Severe: Symptoms and physical signs persist for an even longer period. Severely affects normal work and activities. Will not easily recover with simple treatment.

Researchers must immediately tend to adverse events that occur during the process of the research appropriately and in accordance with medical guidelines. Researchers can increase the number of follow-ups and laboratory tests on the participants if necessary. Follow-ups should be conducted until the adverse event is resolved or until the participant is in stable condition or fully recovers.

7.5 Data Safty Monitoring Board

Data Safty Monitoring Board has been established. This committee will analyze the safety data of the research project 2 times a year. Should the risk of adverse events be clearly larger than the potential benefits of the research project, the Safety Evaluation Committee will recommend that the research be terminated early. Otherwise, the research will continue until the intervention plan is over.

8. Data Management

8.1 Data Management Center

The Data Management Division of Peking University Clinical Research Institute (PUCRI-DM) will be responsible for all data management work of this trial.

8.2 Case Report Form Design and Electronic Database Construction

Investigator will be responsible for drafting a case report form (CRF) for the trial by following the template of the PUCRI-CRF. The draft CRF will be submitted to the PUCRI-DM for their opinions on data management and revision before the final CRF is formed.

The final CRF used by the PUCRI-DM will serve as the foundation for the construction of an electronic database using Epidata software.

8.3 Data Management Plan and Data Validation Plan

The PUCRI-DM is responsible for drafting the "Data Management Plan" and "Data Validation Plan" for the research.

Researchers from all sub-sites are responsible for collecting the primary data for the baseline survey and all follow-ups. They should record the data timely, completely, correctly, and clearly on the paper CRF during the collection process. The PUCRI-DM is responsible for constructing an electronic database based on the paper CRF before the start of the research and supplying it to the various sub-sites. The electronic database will not include the names of the participants which will be replaced with serial numbers coding. The respective sub-sites are responsible for the entry of data from the paper CRF to the electronic database, and double entries will be performed by different person respectively. The electronic database will be submitted to the PUCRI-DM after the data entry process. The PUCRI-DM will compare and verify the data. The PUCRI-DM will consolidate all of the queries they found from the data validation, as well as the queries or accidents during the data entry process into a query form. Researchers from the various sub-sites should address these queries until there are no more queries in the data. The data will then be locked in the electronic database for future data analysis and usage.

The paper CRF will be kept and stored by the respective sub-sites. The paper CRF should be filed according to the serial number of the facilities for elderly and stored in its own locked cabinet. A search catalog should also be filled up for easy reference. The manager of the site must be notified whenever the researchers of the sits have to use the CRF for investigation, record, and data validation purposes. Researchers also have to register at the CRF management logbook. Aside from the personnel responsible for the custody of the paper CRF, no other personnel is allowed to retrieve, read, and modify the data. The PUCRI-DM is responsible for the storage of the electronic data files. This includes the database, verification procedures, analysis procedures, analysis results, codebook, explanatory documents, etc. These files should be classified and stored with multiple backups on different disks or recording media, and be stored appropriately to prevent any damage. Aside from the researchers on this project, no other personnel is allowed to read, use, and modify the electronic data. In addition, the PUCRI-DM shall ensure the safety of the research data.

This plan will be finalized and signed after the PUCRI-DM, researchers, and auditors finalize the research protocol, and before the start of the project. The PUCRI-DM will hold a standardized training on how to fill up and store the paper CRF, enter data into the electronic database, etc. for the researchers of the sub-sites before the start of the baseline survey. They will ensure that the researchers of each sub-site follow the data management requirements specified in this plan.

The "Data Validation Plan" is a document that describes the details of data validation for this

project. It will be drafted by the PUCRI-DM after the research protocol and the research CRF are finalized, and then reviewed by the researchers. The "Data Validation Plan" is a document that outlines the points for the data validation and data validating methods based on the requirements of the research protocol and the specific contents of the CRF.

8.4 Data Entry and Data Audit

All sub-sites will be responsible for data entry, while the PUCRI-DM will be responsible for the data validation. The sub-sites shall follow the rules of a double blind recording method, with two data record staff entering the data at the same time. The PUCRI-DM will then check through and compare the records. For data that is inconsistent between the two records, the PUCRI-DM will verify and modify the data with reference to the paper CRF until both files in the database are identical. Should there be queries or abnormal values in the paper CRF during the data entry process, the PUCRI-DM will send these queries to the field researchers for answering.

8.5 Database Locking and Data Verification

The research data will be frozen after the data of all participants have been entered and all queries have been addressed. Researchers will no longer be permitted to modify the data, so as to guarantee the stability of the research data.

The PUCRI-DM will draft the "Data Management Report" for the frozen database and submit it to the data review committee. Any possible problems with the data in the report will be resolved in a meeting where the researchers, statistician, and PUCRI-DM are present. A division of the statistical population will also be conducted. The database will be locked should the researchers confirm that there are no queries with the data in the frozen database and the division of the statistical population is determined.

9. Statistical Analysis

Baseline Data Analysis:

Participants will be described with respect to demographic information (age, gender, ethnic group, education, etc.), anthropometrics (weight, height, BMI, etc.), physical examination (SBP, DBP, etc.), life style and diet habits, labs (serum potassium, renal function, urinary sodium, urinary potassium, urinary microalbumin, etc.), comorbidities, medication history at baseline, both overall and separately for the intervention and control groups. Categorical data will be summarized by numbers and percentages. Continuous data will be summarized by mean, SD and range if data are normal and median, IQR and range if data are skewed. We will conduct one-way ANOVA on

continuous variables and x2 test on categorical variables to check if the baseline characteristics balanced across randomized groups and to identify potential confounding variables.

Effect Evaluation: Results from ITT analysis will be reported as the primary result and the one from PP analysis will be reported as the secondary result.

Primary outcome is the change in SBP from baseline to follow-up

Secondary outcome includes change in DBP from baseline to follow-up, changes in 24-hr urinary sodium and urinary microalbumin; the risk of developing cardiovascular events

We will use multilevel mixed-effects model to control the cluster effect and confounding variables. We will compare the difference of changes in blood pressure before and after the intervention between SS and non-SS, SSSC and non-SSSC, SS + SSSC and SS only or SSSC only respectively.

Analysis for adherence: Use ITT to analyze the results

We will use multilevel mixed-effects model to control the cluster effect and confounding variables. We will compare the changes in serum potassium levels before and after the intervention, changes in the incidence of hyperkalemia before and after the intervention, and incidences of other possible adverse effect between SS and non-SS. We will use multilevel mixed-effects model to control the cluster effect and confounding variables. We will compare the changes in serum potassium levels before and after the intervention, changes in the incidence of hyperkalemia before and after the intervention, and incidences of other possible adverse reactions between SS + SSSC and SS only or SSSC only.

Safety Evaluation: Use PP to analyze the results

Make use of a multilevel mixed-effects model to control the clustering effect and confounding variables. Compare the changes in blood potassium levels before and after the intervention, changes in the accumulated number of hyperkalemia incidences before and after the intervention, and incidences of other possible adverse reactions between SS and non-SS. Make use of a multilevel mixed-effects model to control the clustering effect and confounding variables. Compare the changes in blood potassium levels before and after the intervention, changes in the accumulated number of hyperkalemia incidences before and after the intervention, and incidences of other possible adverse reactions between SS + SSSC and SS only or SSSC only.

Subgroup Analysis:

Subgroup analysis will be conducted according to the baseline renal function (eGFR) stages to understand the effect modification of baseline renal function.

Cost-effective analysis:

A cost-effectiveness measure will be estimated based on the incremental cost of achieving each 1–mm Hg reduction in SBP. The cost is the fee (unit: Yuan)associated with intervention,

including cost for salt substitute, faculty training, health education, diagnose and treatment for hyperkalemia. Incremental cost-effective ratio (ICER) will be calculated as below:

IC = Cost initial – Cost complete

IE = SBP initial – SBP complete

ICER = IC/IE

10. Sample Size Calculation

The research hypothesis is that the "salt substitute intervention would significantly reduce the systolic blood pressure of patients with hypertension", with the test power of 0.80 and the significance level at 0.05. According to previous studies, consuming salt substitutes for a prolonged period can lower the systolic blood pressure of patients with hypertension by roughly 2~5mmHg. This research hypothesizes that the systolic blood pressure of the elderly participants would decrease by 3.0mmHg, with a standard deviation of 18mmHg. Since the number of groups is 36 and the intra-group correlation coefficient is 0.02, 24 participants are needed in every group calculated by NCSS-PASS software. In addition, considering that about 20% of the participants might be lost to follow-up or be removed from the research due to non-compliance, it is finally determined that 30 participants will be included in each nursing home, for a total of 1080 participants across 36 nursing homes.

Based on the sample size calculated above, the sample size for the four intervention groups is as follows:

		SS intervention		
		Yes	No	Total
SSSC intervention	Yes	270	270	540
	No	270	270	540
	Total	540	540	1080

For the other intervention strategy, it can be estimated from the previous research experience that the SSSC intervention strategy can reduce the systolic blood pressure of the elderly by 4.0mmHg. With the significance level, number of groups, and intra-group correlation coefficient constant, and based on the sample size calculated above, it can be concluded that the power of applying this intervention strategy to reducing systolic blood pressure by 4.0mmHg will be 98.2%.

Given the potential changes of actual systolic blood pressure difference during the trial and

the number of participants recruited from each facility, the changes to the trial power will follow the trend below:

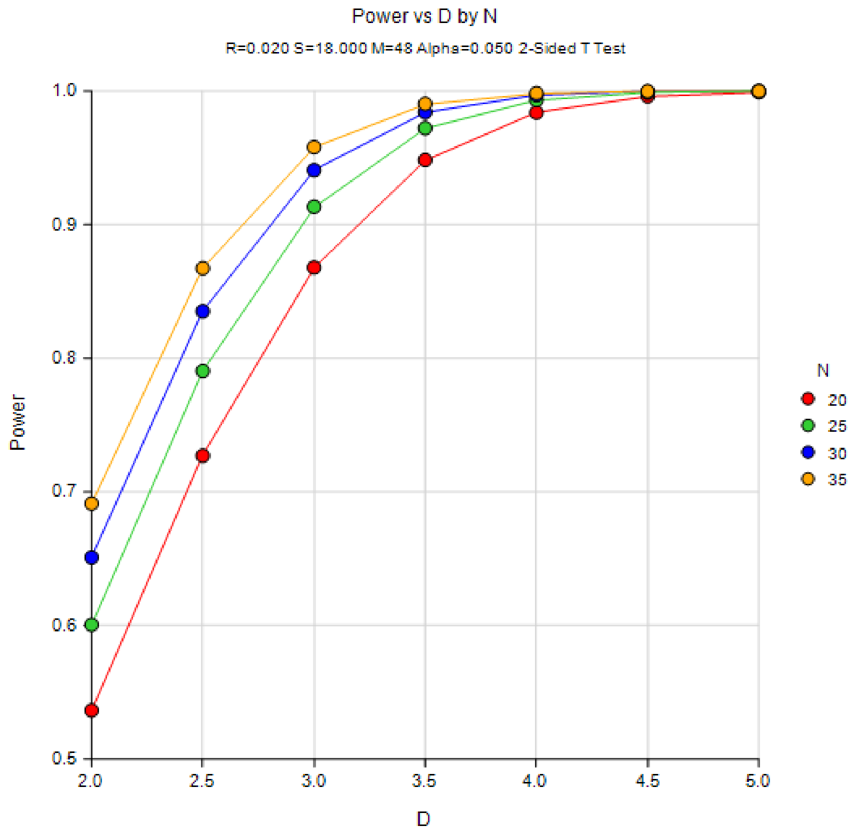

11. Quality Control

11.1 Modifications to the Protocol

The protocol must not be freely modified once it has been approved. If the protocol must be modified after the trial has started because of special cases such as difficulty in execution, an agreement has to be reached by the advisory committee after they discuss the matter. Only then can the protocol be modified or supplemented. The modified content has to be recorded in writing.

The process for modifying the protocol is as follows:

- Noticing that there is a clear problem and understanding the need to modify the protocol;
- Calling for a meeting of the advisory committee to discuss the matter and suggest a way to resolve the issue;
- Principal investigator signs and creates the "Protocol Modification Instructions";
- The modified protocol and "Protocol Modification Instructions" have to be sent to the Ethics

Committee for approval or be put on record;

- The modified protocol can be executed after obtaining the approval of the Ethics Committee.

11.2 Training

The principal investigator is responsible for creating a training plan for the project-related personnel. The experts and project manager appointed by the principal investigator will organize the training, complete the training records, and take custody of the training records in the project management document. All relevant research personnel shall receive their respective training to ensure that they are qualified to undertake the specific tasks assigned to them. The contents of the training for different research personnel are as follows:

- GCP: Principal investigator, managers of all partner organizations, and all staff members
- Subject protection and ethical requirements, including the signing requirement of informed consent, SAE, as well as the handling of protocol violation (PV) and the report requirements: Principal investigator and all staff members
- Research protocol: Principal investigator and all staff members
- Standard operating procedures for the trial, including intervention strategies, trial operation-related standard operating procedures such as blood pressure measurement and testing for 24-hr urinary sodium: Staff members responsible for and participating in the corresponding work
- Instructions on filling up the CRF: Staff members assigned for questioning and filling out the relevant forms
- Things to note on the implementation of the clinical research (e.g. selection of nursing homes and participants, inclusion and follow-up process, etc.): All field staff members
- Project workplan: Principal investigator and all the managers of participating organizations

Aside from these, the principal investigator and project coordinator can increase the training contents if needed during the whole course of the trial. For example, training on the weak links of the trial, training on updation of the protocol, and various targeted trainings on the standard operation procedures.

11.3 Monitoring

The PUCRI will designate a project specialist to monitor this trial, with the main aim of confirming whether the implementation of the trial, data record and analysis conform to the trial protocol and the relevant rules and regulations. Investigatorsshould fully cooperate with the

project specialist.

The project specialist will supervise and visit the research sites based on the "Monitoring Plan" that is drawn up beforehand. This will be done either through on-site visits or off-site visits. The contents of the supervision include:

- Completion and updation of the research document;
- Signing of informed consent form;
- Source data verifciation;
- Distribution and management of the salt substitute;
- Stock in and stock out records for salt and other foods with high-salt content of various nursing homes;
- The implementation of blood potassium monitoring plan for hyperkalemic participants and participants in the high-emphasis monitoring group;
- Occurrence, handling and reporting of SAEs and PVs;

The project specialist will complete a written monitoring report after every visit. The monitoring report will be submitted to all parties involved in project management as required by the "Monitoring Plan".

11.4 Quality Control for Data

A project specialist from the PUCRI will review the completeness and accuracy of the CRF data that is recorded by the researchers according to the monitoringplan. He/she will also guide the staff member who input the data on revising or adding data based on the requirements. The data management specialist from the PUCRI will conduct a logic check on the logical relationship of the CRF data, whether or not the data goes against the protocol, and whether or not it exceeds the normal range of reference. He/she will create a query table for incomplete, missing, or illogical data and request researchers to address, confirm, or correct the data in question. The database can only be locked and analyzed after all of the queries behind the data have been resolved.

12.Subject Protection

12.1 Ethics Review

This research protocol will be submitted to the PKU IRB for review and continuous review. It includes the completed research protocol, CRF, informed consent form, and other documents. The clinical trial can only be commenced after obtaining approval from the IRB. Throughout the implementation of the research, researchers shall abide by the ethical requirements of the GCP and the Declaration of Helsinki and guarantee the rights of the participants. We will regularly

submit a research progress report as requested by the IRB and report the state of the research implementation in a timely manner.

12.2 Informed Consent

This research will seek informed consent from the managers of the nursing homes and all elderly staying in the nursing homes. The informed consent of the nursing homes will be obtained through a partnership agreement with the nursing home. Whether it is from the nursing home or the elderly, the researchers should give a detailed introduction on the purpose of the research, research methods, potential benefits and risks, and what kind of cooperation is expected from them when obtaining the informed consent form. The researchers should also tell them that participating in this clinical research is completely voluntary, that they can withdraw from the experiment at any time, and that refusing or withdrawing from the trial will not affect the treatment or reasonable medical rights of the elderly. The researchers have to address any questions that the other party might have during the process and give the elderly enough time to consider and discuss with their family members. The researchers should also confirm that the other party has decided on their own whether or not to participate in the research after they have a sufficient understanding of the research. Should the elderly be willing to participate in the research, they will be required to sign a hardcopy informed consent form. Illiterate elderly can use their fingerprints or appoint someone to sign on their behalf. The researchers who have obtained informed consent will also have to sign and state the date of signature on the informed consent form at the same time as the other party. The elderly will receive a copy of the informed consent form. The original document will be appropriately kept by the researchers as part of the clinical research documents. Should there be a need to modify the protocol during the research implementation, or if new information is obtained that might influence the participants' continued participation in the research, the researchers have to make the appropriate modification to the informed consent form and seek informed consent from the participants again.

12.3 Confidentiality and Privacy

The personal information of all participants will be kept confidential and stored in a locked cabinet where the research materials are kept. Aside from members of the research group, only the supervisor from the Peking University Health Science Center and the PKU IRB has the right to read through the personal information of the participants. The participants' blood and urine samples will only be used for tests involving the research. Any leftover samples will be destroyed as biological samples on that day. Throughout the implementation of the research, all information

of the participants will be marked using the participants' specific serial numbers or initials. The results of this research will be published in the form of a scientific paper, but all personal information of the participants (including their names, ages, etc.) will remain confidential.

This research will not look into the privacy of the participants. Participants can choose not to answer any questions they deem to be sensitive during the investigation.

13.Trial Management

This research belongs in the topic of "Efficacy, Safety and Health Economics Evaluation of Different Salt Reduction Strategies on Cardiovascular Diseases", under the "Research of Key Nutrition and Action Intervention Techniques and Strategies for Cardiovascular Diseases" of the 2016 National Key R&D Program of China. It is sponsored by the Ministry of Science and Technology of the PRC. Professor Yangfeng WU from the PUCRI has been appointed as the principal investigator. The PUCRI will be the organization leading the project and is responsible for designing the research protocol, technical training, quality control, data analysis, conclusion and publication; the partner organizations of this research include the Changzhi Medical College in Shanxi Province, School of Public Health of Xi'an Jiaotong University in Shaanxi Province, and the Disease Prevention and Control Center of Hohhot City in Inner Mongolia. These three organizations are responsible for supplying the research sites, selecting and managing the participants, implementing the intervention strategies, and collecting research data.

13.1 Advisory Committee

Chairman: Junshi CHEN

Members: Darwin Labarthe, Kiang Liu, Bruce Neal, Minghui ZHAO, Xingshan ZHAO, Wenyi NIU, Yanfang WANG, Hai FANG, Runlin GAO, Xiaofeng LIANG, Yong HUO, Changsheng MA, Jing YANG, Guansheng MA, Jun MA, Jianguo XU

13.2 Data and Safety Monitoring Boards

Chairperson: Mei WANG, Yihong SUN

Members: Luxia ZHANG, Wei ZHAO, Xian LI

13.3 Brief Introduction of sites

1. Changzhi Medical College

The Changzhi Medical College was included in the National Basic Ability Construction Project of Western and Central China in 2016. In the past five years, the college has undertaken 7 projects of the National Natural Science Foundation of China and 86 provincial-level scientific research projects. Personnel from the college have published 2907 essays, of which 239 were included in the SCI. They have also published 77 academic publications, 3 translated works, and 345 pieces of teaching material. The "Journal of Changzhi Medical College" sponsored by this college has

exchanged knowledge on scientific publications with more than 100 colleges in the country.

The Changzhi Medical College has partnered with the principal investigator of this project, Professor Yangfeng WU, on many international projects and has a strong team of workers on the field. Personnel of the college have also participated in the fieldwork of the China Rural Health Initiative-Sodium Reduction Study (CHRI-SRS) in Shanxi Province. They were also responsible for the completion of all fieldworks for the "School-based education program to reduce salt intake in children and their families (School-EduSalt)". The organization is very experienced in on-site investigations and the implementation of salt reduction strategies, and is able to provide a good trial site for this project.

2. School of Public Health of XI'an Jiaotong University

The School of Public Health of XI'an Jiaotong University has tackled key science and technology problems of the country, supported various important specialized projects, undertaken projects from the Natural Science Foundation, tackled ministerial (provincial) problems, and partnered in more than 100 international projects. It has amassed more than 30 million yuan in research funds. It has also gotten 2 prizes of Klaus Schwarz awarded by the International Society of Biological Inorganic Chemistry, as well as more than 10 first and second prizes at the provincial level. Personnel from this school have published more than 2000 scientific papers, with over 400 of them included in the SCI.

The School of Public Health of XI'an Jiaotong University has partnered with the principal investigator of this project, Professor Yangfeng WU, on many international projects and has a strong team of workers on the field. They were also responsible for the fieldwork of the "China Salt Substitute Study" (CSSS) and the CHRI-SRS in Shaanxi Province. The organization is very experienced in on-site investigations and the implementation of salt reduction strategies, and is able to provide a good trial site for this project.

3. Disease Prevention and Control Center of Hohhot City

The Disease Prevention and Control Center of Hohhot City is mainly responsible for the overall management on prevention and control of infectious disease in the Hohhot City, children immunization, implementation of control and monitoring strategies for disinfection and vector organisms, as well as the prevention, control, and technical guidance of chronic non-infectious diseases.

The Disease Prevention and Control Center of Hohhot City has been active in scientific research over the years and the level of their scientific research has been steadily increasing. They have once awarded the first prize of the Hohhot City Science and Technology Progress Award in

2008. They have a young and strong working team in the field of chronic disease prevention and control. This organization is experienced in field investigations and is able to provide a good trial site for the project.

14. Storage of Research Documents and Records

The research organization must establish research documents and record files to ensure accurate reporting, explanation, and verification. These documents are classified into two areas: (1) Original documents which record clinical data such as changes in blood pressure and safety parameters. (2) Management files of the research organization, at least including the revised versions of research documents such as clinical research protocol, CRF, and informed consent forms, approval documents of the Ethics Committee, salt substitute management records, responsibility and authorization documents of the personnel of the research sites, training records, project progress plan and its updates, meeting minutes, etc.

The research organization has to store the above two categories of documents for at least 5 years after the project ends or is terminated. No research documents can be destroyed without the written approval of both the project manager from the PUCRI management office and the principal investigator.

Project management documents such as the supervision reports and progress summary that is made by the PUCRI must be stored at the PUCRI for at least five years after the project ends or is terminated.

15. References

1. Chen W-W, Gao R-L, Liu L-S, et al. China cardiovascular diseases report 2015: a summary. Journal of Geriatric Cardiology : JGC 2017; 14(1): 1-10.

2. Lawes CM, Vander Hoorn S, Rodgers A. Global burden of blood-pressure-related disease, 2001. The Lancet 2008; 371(9623): 1513-8.

3. Wu Y, Benjamin EJ, MacMahon S. Prevention and control of cardiovascular disease in the rapidly changing economy of China. Circulation 2016; 133(24): 2545-60.

4. From burden to "best buys": Reducing the economic impact of NCDs in low- and middle-income countries Executive summary 2011. http://www.who.int/nmh/publications/best_buys_summary/en/

5. Xinhuanet. The report on nutrition and chronic disease of Chinese residents published. http://wwwxinhuanetcom/live/20150630b/indexhtm 2015.

6. Chang HY, Hu YW, Yue CS, et al. Effect of potassium-enriched salt on cardiovascular mortality and medical expenses of elderly men. The American journal of clinical nutrition 2006; 83(6): 1289-96.

7. Group CSSSC. Salt substitution: a low-cost strategy for blood pressure control among rural Chinese. A randomized, controlled trial. Journal of hypertension 2007; 25(10): 2011-8.

8. Zhou B, Wang HL, Wang WL, Wu XM, Fu LY, Shi JP. Long-term effects of salt substitution on blood pressure in a rural north Chinese population. J Hum Hypertens 2013; 27(7): 427-33.

9. Zhao X, Yin X, Li X, et al. Using a low-sodium, high-potassium salt substitute to reduce blood pressure among Tibetans with high blood pressure: a patient-blinded randomized controlled trial. PLoS One 2014; 9(10): e110131.

10. 21 CFR 184.1622 - POTASSIUM CHLORIDE. https://www.gpo.gov/fdsys/search/pagedetails.action?collectionCode=CFR&browsePath=Title+21%2FChapter+I%2FSubchapter+B%2FPart+184%2FSubpart+B%2FSection+184.1622&granuleId=CFR-2010-title21-vol3-sec184-1622&packageId=CFR-2010-title21-vol3&collapse=true&fromBrowse=true

11. Losby JL, Patel MD, Schuldt MJ, Hunt MGS, Stracuzzi MJC, Johnston MY. Sodium-reduction strategies for meals prepared for older adults. Journal of public health management and practice: JPHMP 2014; 20(1 0 1): S23.

Evaluating the Efficacy, Safety, and Cost-Effectiveness of Using a Salt Substitute (SS) and Stepwise Salt Supply Control (SSSC) on Lowering the Blood Pressure of the Elderly in Residential Elderly Care Facilities

Research Organization	Peking University Clinical Research Institute
Principal Investigator	Yangfeng WU
Date of Writing	17-Oct-2019
Version Number	V1.4

CONTENT

Protocol Change Log 1

Protocol Approval Page 2

Protocol Synopsis 3

1.Background 5

2.Trial Purpose 7

2.1 Primary Purpose 7

2.2 Secondary Purpose 8

3.Trial Design 8

4.Participants 9

4.1 Inclusion and Exclusion Criteria of Nursing Homes 9

4.2 Inclusion and Exclusion Criteria of Individual Elderly 10

5.Trial process and data collection 10

5.1 Preparation Period 10

5.2 Baseline Assessments 10

5.3 Randomization 12

5.4 Intervention Scheme 13

5.5 Follow-up 19

6.Outcome Measure 22

6.1 Primary Outcome 22

6.2 Secondary Outcome 22

6.3 Compliance evaluation 23

7. Safety Considerations 23

7.1 Risk-Based Safety Monitoring and Management Plan 23

7.2 Adverse Events 25

7.3 Other Possible Trial-Related Adverse Reactions 25

7.4 Serious Adverse Events 25

7.5 Data Safty Monitoring Board 26

8. Data Management 27
8.1 Data Management Center 27
8.2 Case Report Form Design and Electronic Database Construction 27
8.3 Data Management Plan and Data Validation Plan 27
8.4 Data Entry and Data Audit 28
8.5 Database Locking and Data Verification 28
9. Statistical Analysis 29
10. Sample Size Calculation 30
11. Quality Control 32
11.1 Modifications to the Protocol 32
11.2 Training 32
11.3 Monitoring 33
11.4 Quality Control for Data 33
12.Subject Protection 34
12.1 Ethics Review 34
12.2 Informed Consent 34
12.3 Confidentiality and Privacy 35
13.Trial Management 35
14. Storage of Research Documents and Records 37
15. References 38

Protocol Change Log

Serial Number	Version Number	Revision Date	Author/Editor	Sections or Pages Changed
1	1.1	09/19/2017	Aoming JIN	Page 6, 2.2 Secondary Objectives
2	1.1	09/19/2017	Aoming JIN	Page 9, 5.2 Contents of the Baseline Survey
3	1.1	09/19/2017	Aoming JIN	Page 10, Laboratory Methodology, Fundus Examination Method Section
4	1.1	09/19/2017	Aoming JIN	Page 14, 5.5 Arrangement and Content of Follow-up
5	1.1	09/19/2017	Aoming JIN	Page 15, 6.2 Secondary Outcome
6	1.1	09/19/2017	Aoming JIN	Page 19, 8.3 Data Management Plan, 8.4 Data Entry and Audit
7	1.1	09/19/2017	Aoming JIN	Page 25, 12.3 Confidentiality and Privacy
8	1.2	12/08/2017	Aoming JIN	Page 3, Sample Size
9	1.2	12/08/2017	Aoming JIN	Page 7, 3 Trial Design
10	1.2	12/08/2017	Aoming JIN	Page 8, 4 Inclusion Criteria of Nursing Homes
11	1.2	12/08/2017	Aoming JIN	Page 21, 10 Sample Size Calculation
12	1.2	12/08/2017	Aoming JIN	Page 26, 13 Organizational Management
13	1.2	12/08/2017	Aoming JIN	Page14,18 Intervention Scheme
14	1.3	04/16/2018	Aoming JIN	Page 16, 7.1 Risk-Based Safety Monitoring and Management Plan
15	1.4	10/17/2019	Aoming JIN	Page 18, 7.4 Serious Adverse Events

Protocol Approval Page

I agree with this protocol (Version Number: V1.4, Version Date: 17/10/2019) and will conduct this trial in accordance with the Declaration of Helsinki as well as other relevant laws and regulations.

Peking University Clinical Research Institute

Signature of Principal Investigator:

Name of Principal Investigator (Printed): Yangfeng WU

Date:17-Oct-2019

Protocol Synopsis

Trial Title Evaluating the Efficacy, Safety, and Cost-Effectiveness of Using a Salt Substitute (SS) and Stepwise Salt Supply Control (SSSC) on Lowering the Blood Pressure of the elderly in Nursing Homes

Trial Purpose

The main purpose of the study is to determine the efficacy of using a salt substitute (SS) and stepwise salt supply control (SSSC) on lowering the blood pressure of the older adult living in senior residential facilities. At the same time, the research aims to observe the compliance and safety of the two intervention strategies, as well as evaluate their cost-effectiveness.

Trial Design

This is a multi-center, is a 2-year 2 × 2 factorial, cluster-randomized controlled trial. Senior residential facilities (geracomiums/old folk's homes) are randomized and used to test two salt reduction strategies, namely, 1) introduction of low-sodium salt substitute (SS) compared to continued use of regular salt.; and 2) Manage and control the salt procurement and supply channels of institutional kitchens, which will be achieved together with health education and health promotion for chefs. This will be referred to as the "stepwise salt supply control"

48 eligible senior residential facilities in Changzhi, Xi'an, Hohhot, and Yangcheng will be selected and assigned into one of the following four intervention groups using stratified randomization: 1) SS + SSSC; 2) SS only; 3) SSSC only; and 4) No SS and no SSSC (control). The intervention will be last for 2 years. Follow-ups will be conducted at 6, 12, 18, and 24 months after the intervention.

Sample Size

A total of 48 senior residential facilities and at least 20 members from each senior residential facilities for a total of at least 960 participants will be included in our study.

Participant Selection

Inclusion and Exclusion Criteria of Nursing Homes

Inclusion Criteria:

1) Have more than 20 elderly people staying there for a prolonged period.

2) Have a record of the elderlies' entry and exit from the nursing home, where they went, the reason they left, and the duration they left for.

3) Have a person responsible for the purchase of food and condiments, as well as an area designated for the storage and safekeeping of food.

4) Responsible for preparing the food consumed by the elderly and must not purchase processed food from outside the nursing home more than once a week.

5) Must be willing to accept the intervention to reduce salt usage and have a high degree of cooperation.

6) Signed the partnership agreement.

Exclusion Criteria:

1) Have participated in any salt reduction or other intervention trials in the past or currently.

Inclusion and Exclusion Criteria of Individual Elderly

Inclusion Criteria:

1) Living in the nursing home permanently or expectedly for the coming two years and do not spend more than a month outside of the nursing home.

2) Life expectancy over six months.

3) Age less than 55 years.

Exclusion Criteria:

1) with no data from all the three key measurements: blood pressure, blood sample and 24-hour urine will be excluded.

2) Clinically confirmed hyperkalemia.

Research Duration: 4 years

1.Background

Cardiovascular diseases are currently the leading cause of death in our country, accounting for 42.61% and 45.01%[1] of deaths in cities and rural areas respectively. With the changes in the lifestyles of the Chinese people, as well as the acceleration of the aging population and urbanization, the absolute number of deaths from cardiovascular diseases has been rapidly increasing, up 46% in 2013 as compared to 1990. Amongst those, there has been a 90.9% increase in deaths due to ischemic heart diseases and a 47.7% increase in deaths from cerebrovascular diseases. At the same time, the prevalence of cardiovascular diseases have also been rising steadily over the years. According to estimates, 290 million people have developed cardiovascular diseases, with 13 million developing strokes and 11 million developing coronary heart diseases. Faced with the massive population that have developed the diseases and the continually high death rate, the disease burden of cardiovascular diseases in our country has been increasing over time. It has become an important public health issue, and the prevention and control for it must not be delayed. Hypertension is an important risk factor for cardiovascular diseases. Globally, hypertension is related to 54% of strokes and 47% of ischemic heart attacks[2]. In our country, the prevalence of hypertension is also increasing each year. Based on the latest investigation results on a sample survey for hypertension involving 500000 people in the country, the prevalence rate of hypertension in our country is 23%, with 240 million people having hypertension. Controlling blood pressure has become an important precautionary measure as it will significantly reduce the risk of developing and dying from cardiovascular diseases.

Many researches have shown that an important factor of food consumption on blood pressure is the intake of salt (sodium). Reducing the intake of salt or replacing regular salt with a salt substitute can significantly reduce the blood pressure of patients with hypertension or high-risk individuals, preventing the development of cardiovascular diseases and lowering the number of deaths in the population[3]. The World Health Organization has even regarded reducing salt intake as one of the three "Best Practices" for the prevention of chronic diseases[4]. Despite that, our country still has a high-salt diet. The 2012 National Nutrition Survey revealed that the population

still consumes a high amount of sodium, equaling 14.5 g/d when converted to table salt, which is far higher than the World Health Organization's recommended daily intake of not more than 5 g per day for adults[5]. Thus, it is especially important that we develop and promote suitable and operable salt reduction intervention techniques amongst the high-risk population for cardiovascular diseases in our country.

The prevalence of hypertension is significantly increased amongst the elderly population, which greatly increases the risk of the development and death from cardiovascular diseases. Also, with the gradual deterioration of an individual's sense of taste with age, the elderly will also become less able to self-regulate their intake of sodium. Thus, the elderly population is both a high-risk group for cardiovascular diseases and a key population for the salt reduction strategies.

There have already been many large-scale randomized controlled trials showed that salt substitutes can lower the blood pressure of people with hypertension[6-9]. However, the long-term efficacy of salt substitutes on lowering the blood pressure of the average elderly population remains unclear. Furthermore, the main components of salt substitutes include sodium chloride and potassium chloride. Compared to regular salt, consuming the salt substitute that contains less sodium and more potassium can increase the intake of potassium, which might potentially affect the urine potassium, blood potassium and kidney function. Even though the Food and Drug Administration has deemed salt substitutes containing potassium chloride as safe in 1983 and reported that no adverse reactions were observed in the consumption of salt substitutes amongst the people with normal kidney function, the safety of these intervention strategies on the elderly population still lack ample evidence. Further research is required to obtain the relevant data, so as to build a solid foundation for the large-scale promotion of salt reduction strategies in the future[10].

Currently, salt substitutes on the market contain no more than 30% less sodium chloride than regular salt. Judging from the average salt consumption level of our population, merely using salt substitutes cannot push the Chinese diet toward the recommended salt intake in Chinese Dietary Guidelines. The centralized provision of meals for the elderly in nursing homes makes the kitchen of these nursing homes an optimal trial site for the salt reduction strategies. A research project targeted at reducing the salt intake of the collective elderly population in the US adopted a gradual

and acceptable approach to salt reduction. The intervention strategies used were targeted at the supply of meals for the elderly and the kitchen of the nursing homes. Specifically, the intervention strategies like replacing high-sodium foods with low-sodium foods, improving recipes, and changing the ways cooks prepared the foods were adopted. Ultimately, the sodium content of the meals supplied to the elderly decreased by 9.76% in the first year and 14.17% in the second year[11].

In our country, a large number of elderly live mainly in government-run geracomiums and publicly or privately-run nursing homes. Even though these two kinds of nursing homes are differentiated by whether or not a fee has to be paid for their use, both organizations provide meals for the elderly from their kitchens. Building on the intervention strategies used in past literature as well as the unique features of the facilities that house the elderly in our country, our research group has developed intervention strategies to gradually decrease salt usage and create a stepwise salt supply control. This will be done through controlling the elderly care facilities' purchase of salt and its supply channel and complemented by health education and health promotion for the cooks and the elderly staying in the facilities. The feasibility and efficacy of these salt reduction strategies on reducing the blood pressure of the elderly in Nursing Homes need to be evaluated. If proven to be effective and cost-effective, these salt reduction strategies together could become a new and important measure for the prevention and control of hypertension and cardiovascular disease in our country. It might be possible to lower the community's salt intake to less than 6 grams a day as recommended by the Chinese Nutrition Society, should the combination of these two strategies develop a larger effect. This is an important question that must be answered scientifically.

Thus, this study will evaluate the efficacy and safety of using salt substitutes and a stepwise salt supply control intervention on lowering the risk of cardiovascular diseases in the elderly in Nursing Homes , so as to build a scientific foundation for the large-scale promotion of salt reduction strategies.

2.Trial Purpose

2.1 Primary Purpose

To compare the effects of using only salt substitutes, only the stepwise salt supply control,

and using both intervention strategies in tandem on reducing the blood pressure of the elderly in Nursing Homes against a control group.

2.2 Secondary Purpose

- To investigate if salt substitutes would increase the prevalence rate of adverse reactions such as hyperkalemia, as well as whether the kidney function would affect that effect amongst the elderly in Nursing Homes by comparing the results against a control group.
- To investigate if the salt reduction strategies would decrease the development of cardiovascular diseases and the resulting deaths by comparing the results against a control group.
- To evaluate the degree of compliance and cost-effectiveness of adopting salt substitutes and the stepwise salt supply control in the elderly in Nursing Homes .
- To explore the efficacy of the intervention strategies on proteinuria in the elderly with damaged kidney function and whether the kidney function influences that effect.
- To evaluate the effect of the intervention strategies on fundus atherosclerosis.

3.Trial Design

This is a multi-center, is a 2-year 2 × 2 factorial, cluster-randomized controlled trial. Senior residential facilities (geracomiums/old folk's homes) are randomized and used to test two salt reduction strategies, namely, 1) introduction of low-sodium salt substitute (SS) compared to continued use of regular salt.; and 2) Manage and control the salt procurement and supply channels of institutional kitchens ,which will be achieved together with health education and health promotion for chefs. This will be referred to as the "stepwise salt supply control"

48 eligible senior residential facilities in Changzhi, Xi'an, Hohhot, and Yangcheng will be selected and assigned into one of the following four intervention groups using stratified randomization: 1) SS + SSSC; 2) SS only; 3) SSSC only; and 4) No SS and no SSSC (control). The intervention will be last for 2 years. Follow-ups will be conducted at 6, 12, 18, and 24 months after the intervention.

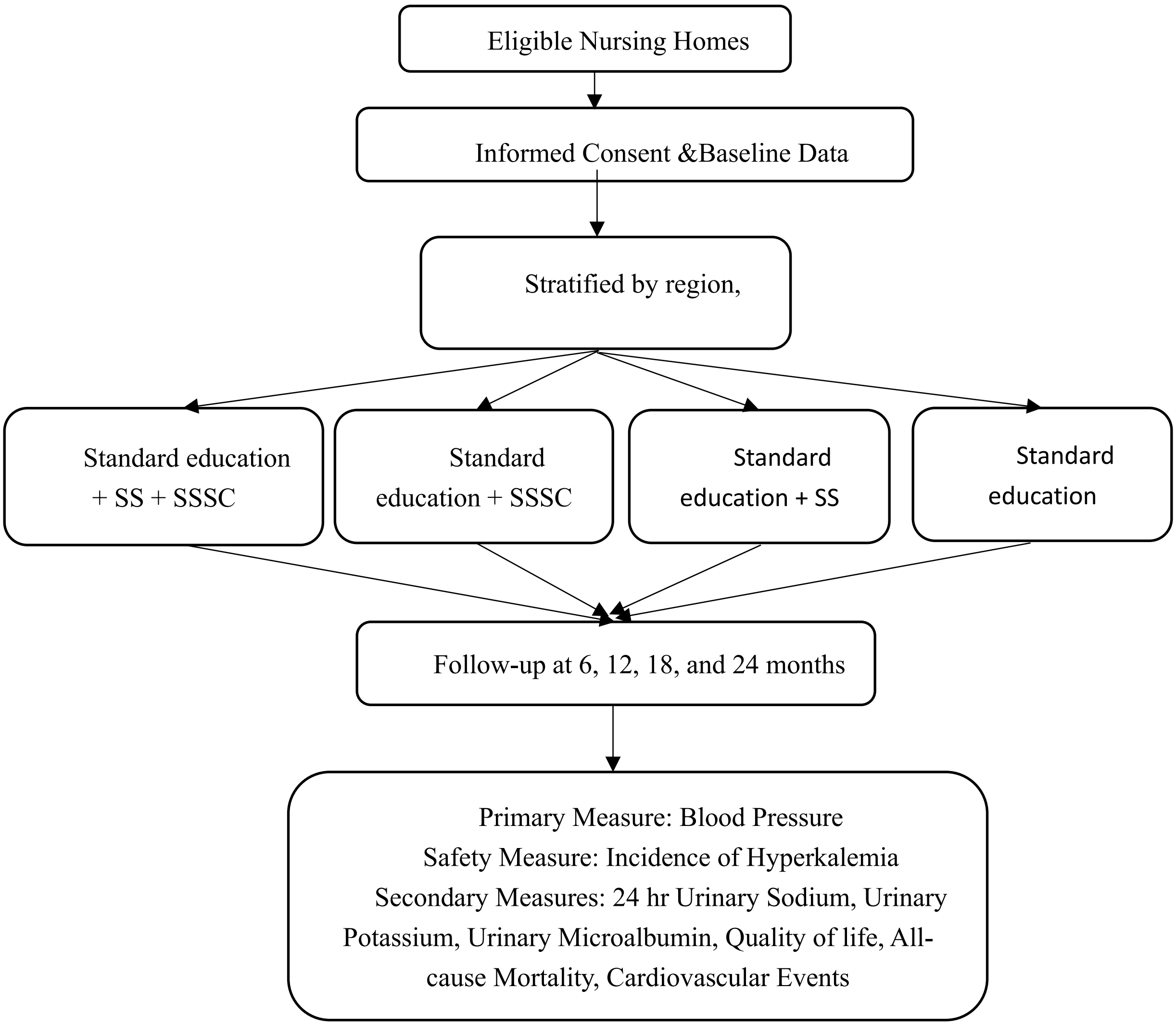

4.Participants

4.1 Inclusion and Exclusion Criteria of Nursing Homes

Inclusion Criteria:

1) Have more than 20 elderly people staying there for a prolonged period.

2) Have a record of the elderlies' entry and exit from the nursing home, where they went, the reason they left, and the duration they left for.

3) Have a person responsible for the purchase of food and condiments, as well as an area

designated for the storage and safekeeping of food.

4) Responsible for preparing the food consumed by the elderly and must not purchase processed food from outside the nursing home more than once a week.

5) Must be willing to accept the intervention to reduce salt usage and have a high degree of cooperation.

6) Signed the partnership agreement.

Exclusion Criteria:

1) Have participated in any salt reduction or other intervention trials in the past or currently.

4.2 Inclusion and Exclusion Criteria of Individual Elderly

Inclusion Criteria:

1) Living in the nursing home permanently or expectedly for the coming two years and do not spend more than a month outside of the nursing home.

2) Life expectancy over six months.

3) Age less than 55 years.

Exclusion Criteria:

1） with no data from all the three key measurements: blood pressure, blood sample and 24-hour urine will be excluded.

2） Clinically confirmed hyperkalemia.

5.Trial process and data collection

5.1 Preparation Period

Relevant organizations in the various regions will be responsible for the selection of eligible nursing homes in their region based on the inclusion criteria. After the nursing homes confirm their participation and sign the partnership agreement, the relevant organizations will head down to the nursing homes and select eligible elderly based on the inclusion and exclusion criteria.

5.2 Baseline Assessments

Baseline assessments will be arranged and conducted after the signing of the partnership agreement and informed consent forms.

Investigations on the nursing homes include:

- Accommodation cost of the elderly
- Types, quantity, and frequency of condiments purchased as well as the frequency and quantity of processed food and foods with high salt content purchased
- Usage of the salt and condiments purchased: Cooking, homemade preserved vegetables or fermented vegetables, feeding livestock

Investigations on the elderly include:

-Contents:

Questionnaire: General Demographic Data: Gender, age, ethnic group, education level, marital status, and medical insurance of the participant.

Lifestyle Information: Smoking, alcohol consumption, level of physical exercise, frequency of fruit and vegetable consumption.

Level of Satisfaction in Meals: How much they liked the meals provided by the nursing home, self-assessment of the level of saltiness in meals.

Disease History: Whether or not they are suffering from hypertension, diabetes, stroke, coronary heart disease, kidney disease, cancer, or COPD. As well as other disease histories and whether they are receiving treatment.

Information on Antihypertensive Drugs: Type, specification, and frequency of consumption of antihypertensive drugs.

Occurrence of an Endpoint Event: Whether or not there is all-cause mortality or cardiovascular events (coronary heart disease, congestive heart failure, heart arrhythmia, and stroke).

Health Status: Will be evaluated using the EuroQol Five Dimensions Questionnaire (EQ-5D).

Physical Examination: Weight, height, blood pressure, heart rate.

Laboratory Examination: Serum electrolytes (blood potassium), blood sugar and four items of blood lipid, kidney function (serum creatinine), and blood routine. Levels of 24-hr urinary sodium, urinary potassium, urinary creatinine, and quantitative microalbuminuria.

Eye Fundus Examination: Vision test and funduscopy.

-Methods:

- Questionnaire and Health Status: Professional investigators who have undergone standardized training will read through and check the nursing homes' travel records and medication information. The participants will be asked for the rest of the information. The information will be further verified through the New Rural Co-operative Medical System.
- Physical Examination: Professional investigators who have undergone standardized training will conduct the physical examination on the participants. See Annex 4 for specific details on the examination methods.
- Laboratory Examination: Participants will be required to empty their bladder and record the time in the morning. They will then be given a 24-hr urine bucket and urine cup to collect their urine over the next 24 hours. The urine collection will be stopped at the same time on the morning of the second day. The urine volume of the participants over 24 hours, left out situations of urine, and the start and end time of the collection will be recorded. After mixing the 24-hr urine sample thoroughly, 5ml of urine will be collected and stored at -20°C. 6.5ml of venous blood will be collected from participants on an empty stomach on the morning of their physical examinations. See Annex 5 for the specific requirements on blood sample collection, packaging, transportation, storage, and determination.
- Eye Fundus Examination: A Chinese standard long-distance visual chart will first be used to investigate the normal vision of the participants. Participants who are wearing glasses during the examination will be tested for their vision with glasses; participants who are not wearing glasses during the examination will be tested for their naked vision. Photographs of the participants' eye fundus were then taken using fundus cameras (TRC-NW Series (Topcon), CR-2 AF Model (Canon), etc.) in non-mydriatic conditions.

5.3 Randomization

The randomization process of this research will be conducted by the Peking University Clinical Research Institute. An independent statistician carried out the randomization after the baseline characteristics had been collected for study sites and individual participants. Nursing homes are

stratified by region before being randomly assigned to the experimental groups.

5.4 Intervention Scheme

Intervention Strategies

There are two main intervention strategies, namely salt substitute and stepwise salt supply control. All nursing homes will also receive general health education on how salt reduction can prevent hypertension and cardiovascular diseases.

1. General Health Education on Salt Reduction

This research will provide a general health education activity to all participating nursing homes (including nursing homes in the control group). The specific details are as follows:

Managers of all the nursing homes will be invited to the trial initiation meeting held at the the study center of the various regions. The aim, design, and requirements of the trial will be explained to them. This includes the health benefits and the existing conflicts that the international research community has on salt reduction and cardiovascular diseases.

At each facility, health education program on salt reduction will be delivered on the baseline survey initiating meeting (with the managers of nursing home, cooks, and all elderly living in the nursing home participated). Posters will also be put up. The main contents of the education program are: Basic health information (The dangers of consuming too much salt, the recommended amount of salt intake in China, and how to reduce salt intake, etc.).

2. Replacing Regular salt with a Salt Substitute

2.1 Salt Substitute management (supply and distribution)

Nursing homes that are allocated to the salt substitute group will use a standardized salt substitute product as assigned by our study. They will do a 100% replacement of the regular salt that they were using. This will be managed and implemented by an assigned professional from the site. Any personnel who are not involved in the trial cannot come into contact with the salt substitute used in this trial. The salt substitute to be used in this trial is a standardized low-sodium salt that contains potassium which can be found on the market. It will be tested by an independent third party to ensure that the product is qualified.

The salt substitute used in the trial must be supplied to the specific nursing homes in strict accordance to the trail requirements. The purchaser and cook of the nursing homes must not hand over or sell the salt substitute. They must not use the salt substitute for something else and most importantly, they must not let non-participants of this research consume the salt substitute.

The trained local investigators will audit the salt supply records once every three months to ensure that the nursing homes are using the salt substitute, so as to evaluate the compliance of the intervention.

2.2 Training on the Correct Usage of the Salt Substitute

This research will provide extra training on the correct usage of the salt substitute for the nursing homes in the SS group. The purchasers and managers of salt in these nursing homes will receive a standardized training and learn about the composition of the salt substitute, its effects, and potential side effects. The core pieces of information are "the salt substitute is not less salty but can lower blood pressure" and "the salt substitute is still a salt, so it's best to still cut down on it".

3. Kitchen-based SSSC program

3.1 Controlling Salt Supply with the Aim of Gradually Reducing Salt Usage in the Kitchens

Assigned personnel from the local study center will be responsible for the target setting and implementation of the salt supply control. This person and the person responsible for the salt substitute intervention strategy cannot be the same person.

➢ Management Target and Plan for Gradual Reduction of Cooking Salt Usage

The main strategy behind this intervention is to control the person purchasing salt for the nursing homes, so as to gradually reduce salt usage in the kitchen in a stepwise manner. The intervention strategy consists of 4 stages. The first stage is the preparatory stage, which lasts for a month. The nursing homes will continue the amount of salt they normally use in their cooking and begin recording how much salt they use to be used as a baseline reference. The second stage consists of two steps, with every step lasting 3 months for a total of 6 months. At this stage, the salt reduction target for every step will be 10% from the nursing home's baseline salt usage. The third stage consists of four steps, with every step lasting 3 months for a total of 12 months. At this

stage, the salt reduction target for every step will be 5% from the nursing home's baseline salt usage. The fourth stage is the maintenance stage. The amount of salt used in the nursing homes should now decrease to 60% of their baseline salt usage. This will not be lowered further until the end of the research. (As per diagram below)

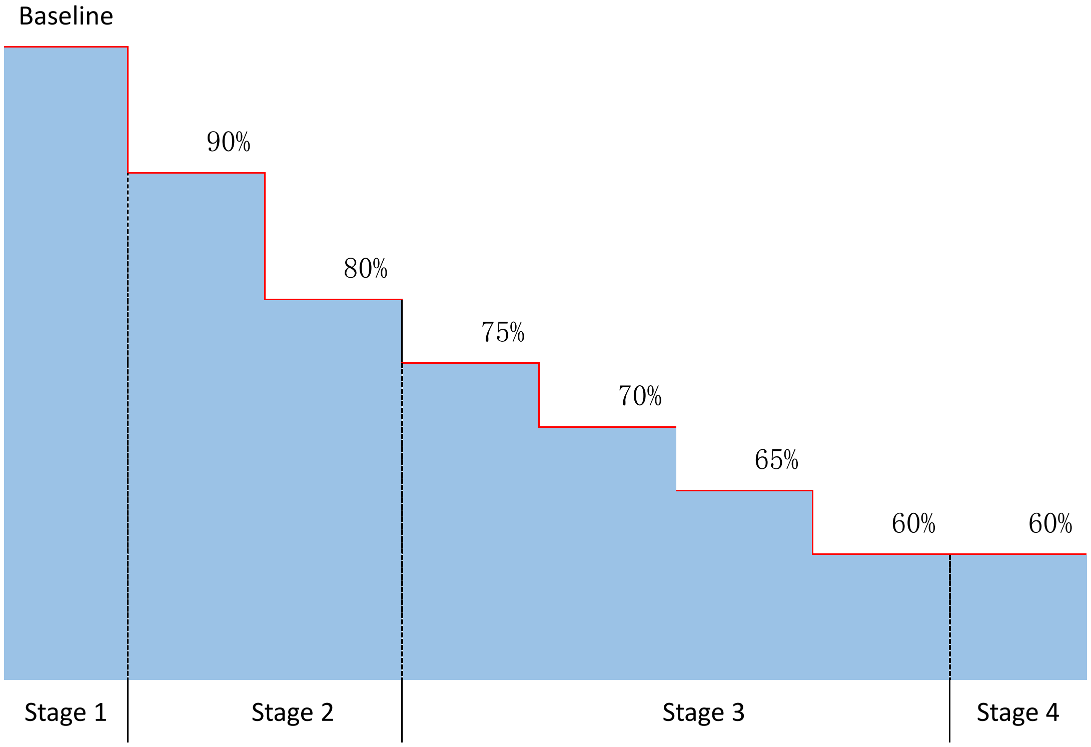

Kitchen-based stepwise salt supply control program.

➢ Management of Stepwise Salt Supply Control and evaluation

Nursing homes that have been assigned to the SSSC group will send their purchasers and cooks to receive standardized technical training. See Annex 2 (Stepwise Salt Supply Control Technical Training) for detailed contents of the training.

Through the training, purchasers will learn and understand the salt reduction targets throughout the various stages and the specific ways to control salt supply; and cooks will learn how to weigh 5% and 10% of their ordinary salt usage. The two parties have to learn the reason for salt reduction and techniques to communicate with the elderly.

The person-in-charge from the sub-sites will audit the stock in and stock out records once every three months to evaluate if the goal for the stage has been met. This will be the basis for the

decision on whether to activate the next stage of the intervention and intervention target. Nursing homes are considered to have met the target if the difference between their actual salt usage and the target salt usage is no more than ±30% and can move on to the next stage. A forum will also be held for the elderly to gather feedback and opinions. For nursing homes that fail to meet the target, a forum has to be held between the managers of the nursing homes, personnel controlling the supply of salt, cooks, and an elderly representative to identify challenges and resolve them. These nursing homes will also keep their original salt reduction target for the next 3 months. They will only go on to the next target after they meet their current target. Should a nursing home fail to meet half of the expected target for 2 consecutive stages, the intervention strategy will end for the nursing home at their current salt usage level. Should a nursing home exceed the salt reduction target of their current stage, they will be requested to slow down their rate of salt reduction and comply with the planned target.

➢ Incentives

Results-based incentives for the purchasers and cooks will be set up in accordance with the salt reduction effects and progress of the nursing homes.

3.2 Maintaining a Stock in and Stock out Record for the Purchase of Salt, Condiments, and Foods with High Salt Content, as well as A Record for the Number of Diners per Meal

Nursing homes that are assigned to the SSSC group have to establish a stock in and stock out record for condiments and foods with high salt content, as well as a record for the number of diners per meal. They will also have to record this information truthfully on the standardized stock in and stock out record form and diner record form provided by the research group (see Annex 3).

Items that have to be recorded in the stock in form include:

- Table salt
- Condiments (e.g. soy sauce, chicken bouillon, MSG, fermented chili bean paste, chili paste, sweet bean sauce, etc.)
- Foods with high salt content (e.g. salted vegetables, pickled vegetables, preserved eggs, fermented vegetables, etc.)

- Processed foods (e.g. instant noodles, vermicelli, bread, sausages, etc.)

Purchasers have to keep a detailed record of the item type purchased, date, quantity, and number of items left on the purchase record form.

Salt and condiments have to be placed in locked storage rooms and managed by the purchasers. Cooks are not allowed to obtain these items on their own. If the condiments in the kitchen have been used up and needs to be replenished, the cook has to inform the manager about the type and amount of condiment needed. The purchaser will procure the required condiment from the storage room and replenish the stock in the kitchen. At the same time, the purchaser has to record the type, date, and quantity of the item that was taken out of the storage room in detail in the stock out form. Aside from that, a daily record of the number of diners in the nursing homes has to be kept (includes the elderly and staff).

The stock in, stock out, and number of diners will be recorded immediately after the randomization has been done and will end when the intervention is over.

3.3 Stepwise Salt Supply Control Plus Health Education

- An extra and targeted health education will be supplied to nursing homes assigned to the SSSC group. Its aim is to increase the degree of compliance of the members of the nursing homes to the intervention measures and reduce the potential obstacles and resistance. This event will be executed by the personnel responsible for the SSSC intervention strategy from the sub-sites.
- Health Education Target Audience. Includes managers of nursing homes, purchasers, cooks, as well as the elderly living in the nursing homes for a prolonged period and their caretakers.
- health education activity.

1) An SSSC initiation meeting will be held in every nursing home and all personnel stated above will be invited. A standardized lesson will be conducted on the benefits of consuming less salt, the overall aim of the activity, etc;

2) A forum for the elderly will be held during the inspection once every 3 months to obtain feedback and opinions about the salt reduction intervention, as well as to launch the targeted

health education;

3) Release health education publicity materials during appropriate times: Such as promotional posters, pamphlets, etc;

4) Along with technical training, engage in targeted health education with the managers, purchasers, and cooks of the nursing home. Muster an subjective initiative to launch various salt reduction strategies on their own and increase the level of awareness and thought for the elderly's health.

4. Salt Supply

During the intervention period, the salt (regular salt and salt substitute) was uniformly produced and provided free of charge by suppliers designated by Peking University Clinical Research Institute. Suppliers have to ensure that they have a sufficient supply of the salt substitute during the intervention period, and there should not be a shortage and out of stock.

5.5 Follow-up

- Follow-up timings: 6, 12, 18, and 24 months after the randomization

  Follow-up content: See the Table below.
- Follow-up method:

Questionnaire: Professional investigators who have undergone standardized training will read through and check the travel records and medication information of the nursing homes, as well as question the participants. The information will be further verified through the New Rural Co-operative Medical System.

Physical examination: A physical examination will be conducted on participants. Laboratory Examination: Participants will be required to empty their bladder and record the time in the morning. They will then be given a 24-hr urine bucket and urine cup to collect their urine over the next 24 hours. The urine collection will be stopped at the same time on the morning of the second day. The urine volume of the participants over 24 hours, left out situations of urine, and the start and end time of the collection will be recorded. After mixing the 24-hr urine sample thoroughly, 5ml of urine will be collected using a catheter and stored at -20℃. 4ml of venous blood will be collected from participants on an empty stomach on the morning of their physical examinations.

Research Follow-up Form

Time / Items	Preparation Period	Baseline	Follow-up Period						Early Withdrawal
			Month 6	Month 12	Month 18	Month 24	At any time	Transferring to Another Hospital	
Signing of Informed Consent Form	√								
Confirmation of Inclusion and Exclusion Criteria	√								
Basic Information Page	√								
Demographic Data		√							
Lifestyle		√							
Satisfaction in Meals		√	√	√	√	√			√
History of Disease and Treatment		√							
EuroQol Five Dimensions Questionnaire (EQ-5D)		√		√		√			√

Height, Weight		√				√			√
Blood Pressure, Heart Rate		√	√	√	√	√			√
Blood Routine		√				√			√
Blood Sugar, Four Items of Blood Lipid		√				√			√
24hr Urine		√				√			√
Eye Fundus Examination		√				√			√
Antihypertensive Drug Information		√	√	√	√	√			√
Collection of Hospitalization Records		√						√	
Reports of Adverse Reactions							√		
CRF Signature Page						√			√

- Early Withdrawal from Trial

- Criteria for early withdrawal:
    1. Researchers determine that it is not suitable for the research to continue because of adverse events;
    2. Participants request to withdraw from the research;
    3. Going against the protocol and others.
- The reason for early withdrawal should be recorded for participants who withdraw from the trial after meeting the early withdrawal criteria. And the following examination items should be performed and recorded during the last visit to the best of the ability:
    – Laboratory examination: Blood routine, kidney function, blood sugar, blood lipid, 24-hr urine
    – Physical examination: Height, weight, blood pressure, heart rate
    – Information about antihypertensive drug consumption, satisfaction in meals
    – EuroQol Five Dimensions Questionnaire (EQ-5D)
    – Endpoint events, safety incidents
    – Monitoring and record of adverse events

6.Outcome Measure

6.1 Primary Outcome

The primary outcome for the study is the difference of SBP between baseline and at 24 months of intervention.

6.2 Secondary Outcome

1. The incidence of hyperkalemia : as the main safety evaluation index. According to the 2012 Kidney Disease Improving Global Outcomes (Kidney Disease Improving Global Outcomes), serum potassium> 5.5 mmol/L is diagnosed as hyperkalemia; continuous measurement of serum potassium> 5.5 mmol/L is persistent hyperkalemia. Otherwise, it is a single attack.
2. The change in 24-hour urinary sodium、potassium and microalbumin
3. The change in diastolic blood pressure
4. European Five-Dimensional Health Scale (EQ-5D) score
5. Endpoint events (death, cardiovascular events (coronary heart disease, congestive heart failure, arrhythmia, stroke))
6. Fundus Atherosclerosis

6.3 Compliance evaluation

- Proportion of nursing homes that 100% use salt substitute in the SS group
- The proportion of nursing homes that reduce their salt usage by more than 20% in the SSSC group
- Satisfaction level of the elderly in intervention groups

7. Safety Considerations

7.1 Risk-Based Safety Monitoring and Management Plan

- **All participants will be screened for hyperkalemia and its related risk factors during the baseline investigation, Month 12 follow-up, and Month 24 follow-up.**

All participants enrolled in the trial will be tested for blood potassium and blood creatinine levels during the baseline investigation. Meanwhile, the information about the antihypertensive drugs (ACEI, ARB, potassium-sparing diuretics, etc.) taken by the participants will also be collected.

All participants enrolled in this trial will be tested once again for blood potassium and blood creatinine levels during the Month 12 and Month 24 follow-up.

- **Based on the results of the screening, any elderly who fit the clinical diagnostic criteria for hyperkalemia will be transferred to a local secondary hospital or above for further blood potassium and ECG testing. They will then be treated for hyperkalemia confirmed by the doctor. The elderly who are not diagnosed with hyperkalemia but fit any of the following conditions will be designated as the high risk group for monitoring.**

1. Participants with blood potassium >5.5mmol/L;
2. Participants with 15<=eGFR<30mL/min/1.73m^2 (Patients with Stage 4 chronic renal insufficiency).

The high risk group for monitoring will receive a blood potassium test every 3 months. Participants who are clinically diagnosed with hyperkalemia will be transferred to a local secondary hospital or above for hyperkalemia treatment. Participants who are not diagnosed with hyperkalemia will continue to be monitored for blood potassium 3 months later. The monitoring can be stopped temporarily should the blood potassium level of the participants <=5.5mmol/L in two consecutive tests. Otherwise, they will continue to be tested for blood potassium once every 3 months.

- **Blood potassium monitoring plan for the SS intervention group:**

All participants in the SS intervention group, aside from those who are confirmed to have

hyperkalemia or are placed in the high risk group for monitoring during the baseline examination, will go through an extra safety screening during the third and sixth month after the start of the intervention. Screening method: questionnaire and blood potassium test. A questionnaire will first be given to the participants during the screening. The contents of the questionnaire include whether or not participants had symptoms and physical signs of hyperkalemia and kidney disease, as well as what medication they are currently taking. If the questionnaire reveals that the participants have obvious symptoms, physical signs, and a clear inducement of hyperkalemia (i.e. at least one "Yes" to the questions in the second section of the questionnaire), a blood potassium test will have to be conducted for them. For participants whose questionnaires do not reveal the risk of hyperkalemia, they can be tested for blood potassium if they are willing to draw blood. All participants of the SS intervention group have to go through at least one blood potassium test during the two monitorings. (See Annex 16.6 for details about the questionnaire) Based on the monitoring results (clinical diagnosis of hyperkalemia or high risk group for monitoring), participants will undergo the relevant risk monitoring and treatment as mentioned above.

- **Clinical Diagnosis of Hyperkalemia and Handling Scheme (According to the 2012 clinical guidelines of the Kidney Disease Improving Global Outcomes)**

- Participant with a serum potassium of >5.5mmol/L:

Immediately notify the nursing home where the participant resides in. The nursing home should take the participant to the hospital for another blood potassium test and ECG examination.

- Should the doctor diagnose the participant sent to the hospital with hyperkalemia based on the blood potassium test and other symptoms, the participant will be recommended to immediately undergo treatment such as taking hydrochlorothiazide, sodium polystyrene sulfonate, or if necessary, undergo emergency intravenous infusion to lower potassium levels. If the participant is currently taking antihypertensive drugs like ACEI/ARB, he/she will be required to stop taking those medicines and switch to antihypertensive drugs such as calcium channel blockers, α-receptor blockers, and β-receptor blockers or αβ-receptor blockers. The participant should also be advised to pay attention to their diet to avoid fresh jujube, peach, banana, tomato, soy, tofu, corn, seafood, bacon and other potassium-rich fruits, vegetables, fish, shrimps, crabs, meats, and nuts. The hyperkalemic patient can return to the nursing home after recovery. A review will be done two weeks later. If the participant's blood potassium level is once again >5.5mmol/L and the participant is in the SS intervention group, then he/she will go through treatment to reduce potassium levels and be terminated from the research. Otherwise, the participant will continue to be referred for potassium-lowering treatment and continue to be monitored.

- **Early termination of research**

The Participants will be stopped from SS intervention if they presented with three consecutive serum potassium measurements >5.5mmol/L. The participants can discuss with the nursing home to be transferred to a nursing home that is not participating in the research. Alternatively, the cooks of the nursing home may prepare food using regular salt just for the participants.

7.2 Adverse Events

Adverse events are any adverse medical incidents that happen to the participant after participating in the research. It does not have to have a causal relationship with the intervention strategies.

Adverse events can be any of the following adverse and unexpected physical signs (including abnormal laboratory data), symptoms, or diseases. It does not have to be related to the intervention strategies.

7.3 Other Possible Trial-Related Adverse Reactions

Gastrointestinal adverse reactions: Participants complain about symptoms such as nausea, vomiting, and diarrhoea. Should these reactions occur, the nursing home should be notified to send the participants to the hospital for treatment. Get a doctor to diagnose if the symptoms are a result from the intervention strategies. If these adverse reactions are due to the intervention strategies of this research, then the participants must be stopped for the reseach. The participants can discuss with the nursing home to be transferred to a nursing home that is not participating in the research. Alternatively, the cooks of the nursing home may prepare food using regular salt just for the participants.

Changes in blood pressure: The blood pressure of participants who regularly take antihypertensive drugs must be routinely monitored during the research period. If there is a significant decrease in their blood pressure, notify the nursing home and send them to the hospital. Get a doctor to gradually adjust the prescription of the drugs to prevent any fainting incidents that might result from an overly low blood pressure. After the research has ended, conduct a follow-up on the participants who regularly take antihypertensive drugs and monitor the changes in their blood pressure after the salt reduction strategies have ended. The prescription of their drugs should be adjusted to prevent any sudden increase in their blood pressure.

7.4 Serious Adverse Events

A serious adverse event (SAE) is defined as having any of the following outcomes resulting from the intervention:

- Threat to life
- Requiring hospitalization
- Permanent or significant disability and dysfunction

Should an SAE occur during the course of the research, researchers must adopt the suitable treatment measure on the participant immediately and end the trial.

Should an SAE occur, researchers have to find out the details and record the following content, whether or not it has to do with the intervention strategies: Time of occurrence, end time, measures adopted, relationship with the investigational drugs, etc. For expected SAEs that are unrelated to the research, researchers have to conduct the appropriate recordkeeping and report it to the Ethics Committee after the research is over. For unexpected SAEs or SAEs that are related to the research, a report has to be made in accordance with the provisions of the Ethics Committee.

Should any adverse events occur, researchers have to record the following on the CRF: Time of occurence, level of severity, end time, measures adopted, and outcome.

The severity of adverse events can be classified into the following 3 levels:

- Mild: Light symptoms and physical signs. Usually a one-off event that does not require treatment and will not affect daily activities. Symptoms would automatically be relieved with rest;
- Moderate: Symptoms and physical signs persist for a longer time. Slightly affects daily activities. Will recover with simple treatment;
- Severe: Symptoms and physical signs persist for an even longer period. Severely affects normal work and activities. Will not easily recover with simple treatment.

Researchers must immediately tend to adverse events that occur during the process of the research appropriately and in accordance with medical guidelines. Researchers can increase the number of follow-ups and laboratory tests on the participants if necessary. Follow-ups should be conducted until the adverse event is resolved or until the participant is in stable condition or fully recovers.

7.5 Data Safty Monitoring Board

Data Safty Monitoring Board has been established. This committee will analyze the safety data of the research project 2 times a year. Should the risk of adverse events be clearly larger than the potential benefits of the research project, the Safety Evaluation Committee will recommend that the research be terminated early. Otherwise, the research will continue until the intervention plan is over.

8. Data Management

8.1 Data Management Center

The Data Management Division of Peking University Clinical Research Institute (PUCRI-DM) will be responsible for all data management work of this trial.

8.2 Case Report Form Design and Electronic Database Construction

Investigator will be responsible for drafting a case report form (CRF) for the trial by following the template of the PUCRI-CRF. The draft CRF will be submitted to the PUCRI-DM for their opinions on data management and revision before the final CRF is formed.

The final CRF used by the PUCRI-DM will serve as the foundation for the construction of an electronic database using Epidata software.

8.3 Data Management Plan and Data Validation Plan

The PUCRI-DM is responsible for drafting the "Data Management Plan" and "Data Validation Plan" for the research.

Researchers from all sub-sites are responsible for collecting the primary data for the baseline survey and all follow-ups. They should record the data timely, completely, correctly, and clearly on the paper CRF during the collection process. The PUCRI-DM is responsible for constructing an electronic database based on the paper CRF before the start of the research and supplying it to the various sub-sites. The electronic database will not include the names of the participants which will be replaced with serial numbers coding. The respective sub-sites are responsible for the entry of data from the paper CRF to the electronic database, and double entries will be performed by different person respectively. The electronic database will be submitted to the PUCRI-DM after the data entry process. The PUCRI-DM will compare and verify the data. The PUCRI-DM will consolidate all of the queries they found from the data validation, as well as the queries or accidents during the data entry process into a query form. Researchers from the various sub-sites should address these queries until there are no more queries in the data. The data will then be locked in the electronic database for future data analysis and usage.

The paper CRF will be kept and stored by the respective sub-sites. The paper CRF should be filed according to the serial number of the facilities for elderly and stored in its own locked cabinet. A search catalog should also be filled up for easy reference. The manager of the site must be notified whenever the researchers of the sits have to use the CRF for investigation, record, and

data validation purposes. Researchers also have to register at the CRF management logbook. Aside from the personnel responsible for the custody of the paper CRF, no other personnel is allowed to retrieve, read, and modify the data. The PUCRI-DM is responsible for the storage of the electronic data files. This includes the database, verification procedures, analysis procedures, analysis results, codebook, explanatory documents, etc. These files should be classified and stored with multiple backups on different disks or recording media, and be stored appropriately to prevent any damage. Aside from the researchers on this project, no other personnel is allowed to read, use, and modify the electronic data. In addition, the PUCRI-DM shall ensure the safety of the research data.

This plan will be finalized and signed after the PUCRI-DM, researchers, and auditors finalize the research protocol, and before the start of the project. The PUCRI-DM will hold a standardized training on how to fill up and store the paper CRF, enter data into the electronic database, etc. for the researchers of the sub-sites before the start of the baseline survey. They will ensure that the researchers of each sub-site follow the data management requirements specified in this plan.

The "Data Validation Plan" is a document that describes the details of data validation for this project. It will be drafted by the PUCRI-DM after the research protocol and the research CRF are finalized, and then reviewed by the researchers. The "Data Validation Plan" is a document that outlines the points for the data validation and data validating methods based on the requirements of the research protocol and the specific contents of the CRF.

8.4 Data Entry and Data Audit

All sub-sites will be responsible for data entry, while the PUCRI-DM will be responsible for the data validation. The sub-sites shall follow the rules of a double blind recording method, with two data record staff entering the data at the same time. The PUCRI-DM will then check through and compare the records. For data that is inconsistent between the two records, the PUCRI-DM will verify and modify the data with reference to the paper CRF until both files in the database are identical. Should there be queries or abnormal values in the paper CRF during the data entry process, the PUCRI-DM will send these queries to the field researchers for answering.

8.5 Database Locking and Data Verification

The research data will be frozen after the data of all participants have been entered and all queries have been addressed. Researchers will no longer be permitted to modify the data, so as to guarantee the stability of the research data.

The PUCRI-DM will draft the "Data Management Report" for the frozen database and submit it to the data review committee. Any possible problems with the data in the report will be resolved in a meeting where the researchers, statistician, and PUCRI-DM are present. A division of the

statistical population will also be conducted. The database will be locked should the researchers confirm that there are no queries with the data in the frozen database and the division of the statistical population is determined.

9. Statistical Analysis

Baseline Data Analysis:

Participants will be described with respect to demographic information (age, gender, ethnic group, education, etc.), anthropometrics (weight, height, BMI, etc.), physical examination (SBP, DBP, etc.), life style and diet habits, labs (serum potassium, renal function, urinary sodium, urinary potassium, urinary microalbumin, etc.), comorbidities, medication history at baseline, both overall and separately for the intervention and control groups. Categorical data will be summarized by numbers and percentages. Continuous data will be summarized by mean, SD and range if data are normal and median, IQR and range if data are skewed. We will conduct one-way ANOVA on continuous variables and x2 test on categorical variables to check if the baseline characteristics balanced across randomized groups and to identify potential confounding variables.

Effect Evaluation: Results from ITT analysis will be reported as the primary result and the one from PP analysis will be reported as the secondary result.

Primary outcome is the change in SBP from baseline to follow-up

Secondary outcome includes change in DBP from baseline to follow-up, changes in 24-hr urinary sodium and urinary microalbumin; the risk of developing cardiovascular events

We will use multilevel mixed-effects model to control the cluster effect and confounding variables. We will compare the difference of changes in blood pressure before and after the intervention between SS and non-SS, SSSC and non-SSSC, SS + SSSC and SS only or SSSC only respectively.

Analysis for adherence: Use ITT to analyze the results

We will use multilevel mixed-effects model to control the cluster effect and confounding variables. We will compare the changes in serum potassium levels before and after the intervention, changes in incidence of hyperkalemia before and after the intervention, and incidences of other possible adverse effect between SS and non-SS. We will use multilevel mixed-effects model to control the cluster effect and confounding variables. We will compare the changes in serum potassium levels before and after the intervention, changes in the incidence of hyperkalemia before and after the intervention, and incidences of other possible adverse reactions between SS + SSSC and SS only or SSSC only.

Safety Evaluation: Use PP to analyze the results

Make use of a multilevel mixed-effects model to control the clustering effect and confounding variables. Compare the changes in blood potassium levels before and after the intervention, changes in the accumulated number of hyperkalemia incidences before and after the intervention, and incidences of other possible adverse reactions between SS and non-SS. Make use of a multilevel mixed-effects model to control the clustering effect and confounding variables. Compare the changes in blood potassium levels before and after the intervention, changes in the accumulated number of hyperkalemia incidences before and after the intervention, and incidences of other possible adverse reactions between SS + SSSC and SS only or SSSC only.

Subgroup Analysis:

Subgroup analysis will be conducted according to the baseline renal function (eGFR) stages to understand the effect modification of baseline renal function.

Cost-effective analysis:

A cost-effectiveness measure will be estimated based on the incremental cost of achieving each 1–mm Hg reduction in SBP. The cost is the fee (unit: Yuan)associated with intervention, including cost for salt substitute, faculty training, health education, diagnose and treatment for hyperkalemia. Incremental cost-effective ratio (ICER) will be calculated as below:

IC = Cost initial – Cost complete

IE = SBP initial – SBP complete

ICER = IC/IE

10. Sample Size Calculation

The research hypothesis is that the "salt substitute intervention would significantly reduce the systolic blood pressure of patients with hypertension", with the test power of 0.80 and the significance level at 0.05. According to previous studies, consuming salt substitutes for a prolonged period can lower the systolic blood pressure of patients with hypertension by roughly 2~5mmHg. This research hypothesizes that the systolic blood pressure of the elderly participants would decrease by 3.0mmHg, with a standard deviation of 18mmHg. Since the number of groups is 48 and the intra-group correlation coefficient is 0.02, 16 participants are needed in every group calculated by NCSS-PASS software. In addition, considering that about 20% of the participants might be lost to follow-up or be removed from the research due to non-compliance, it is finally determined that 20 participants will be included in each nursing home, for a total of 960 participants across 48 nursing homes.

Based on the sample size calculated above, the sample size for the four intervention groups is as follows:

		SS intervention		
		Yes	No	Total
SSSC intervention	Yes	240	240	480
	No	240	240	480
	Total	480	480	960

For the other intervention strategy, it can be estimated from the previous research experience that the SSSC intervention strategy can reduce the systolic blood pressure of the elderly by 4.0mmHg. With the significance level, number of groups, and intra-group correlation coefficient constant, and based on the sample size calculated above, it can be concluded that the power of applying this intervention strategy to reducing systolic blood pressure by 4.0mmHg will be 96.6%.

Given the potential changes of actual systolic blood pressure difference during the trial and the number of participants recruited from each facility, the changes to the trial power will follow the trend below:

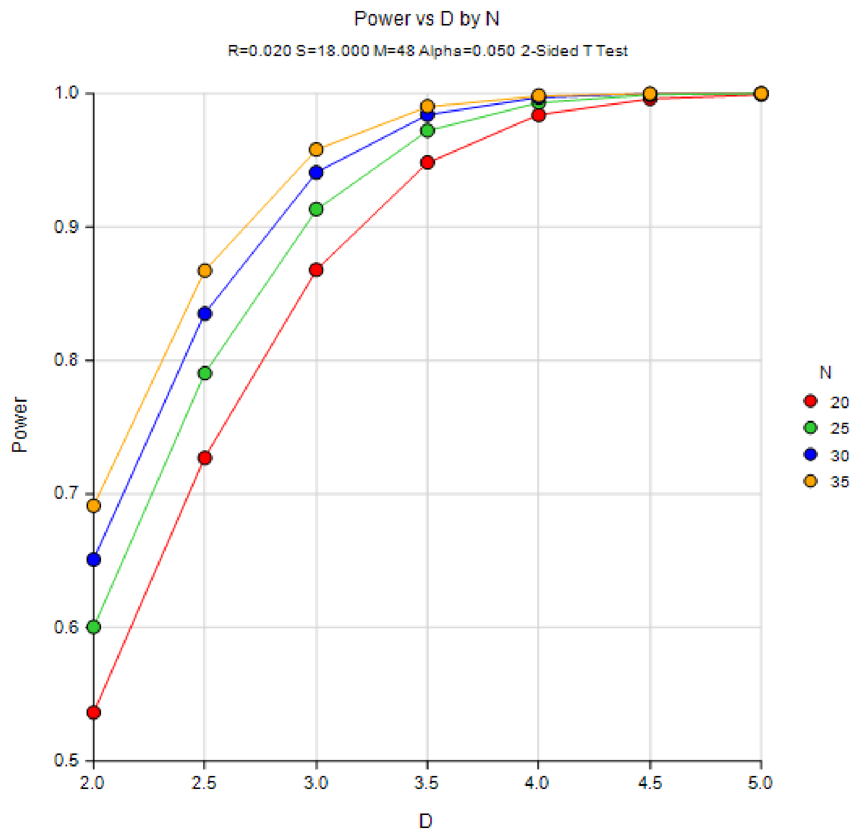

11. Quality Control

11.1 Modifications to the Protocol

The protocol must not be freely modified once it has been approved. If the protocol must be modified after the trial has started because of special cases such as difficulty in execution, an agreement has to be reached by the advisory committee after they discuss the matter. Only then can the protocol be modified or supplemented. The modified content has to be recorded in writing.

The process for modifying the protocol is as follows:

- Noticing that there is a clear problem and understanding the need to modify the protocol;
- Calling for a meeting of the advisory committee to discuss the matter and suggest a way to resolve the issue;
- Principal investigator signs and creates the "Protocol Modification Instructions";
- The modified protocol and "Protocol Modification Instructions" have to be sent to the Ethics Committee for approval or be put on record;
- The modified protocol can be executed after obtaining the approval of the Ethics Committee.

11.2 Training

The principal investigator is responsible for creating a training plan for the project-related personnel. The experts and project manager appointed by the principal investigator will organize the training, complete the training records, and take custody of the training records in the project management document. All relevant research personnel shall receive their respective training to ensure that they are qualified to undertake the specific tasks assigned to them. The contents of the training for different research personnel are as follows:

- GCP: Principal investigator, managers of all partner organizations, and all staff members
- Subject protection and ethical requirements, including the signing requirement of informed consent, SAE, as well as the handling of protocol violation (PV) and the report requirements: Principal investigator and all staff members
- Research protocol: Principal investigator and all staff members
- Standard operating procedures for the trial, including intervention strategies, trial operation-related standard operating procedures such as blood pressure measurement and testing for 24-hr urinary sodium: Staff members responsible for and participating in the corresponding work
- Instructions on filling up the CRF: Staff members assigned for questioning and filling out the relevant forms

- Things to note on the implementation of the clinical research (e.g. selection of nursing homes and participants, inclusion and follow-up process, etc.): All field staff members
- Project workplan: Principal investigator and all the managers of participating organizations

Aside from these, the principal investigator and project coordinator can increase the training contents if needed during the whole course of the trial. For example, training on the weak links of the trial, training on updation of the protocol, and various targeted trainings on the standard operation procedures.

11.3 Monitoring

The PUCRI will designate a project specialist to monitor this trial, with the main aim of confirming whether the implementation of the trial, data record and analysis conform to the trial protocol and the relevant rules and regulations. Investigatorsshould fully cooperate with the project specialist.

The project specialist will supervise and visit the research sites based on the "Monitoring Plan" that is drawn up beforehand. This will be done either through on-site visits or off-site visits. The contents of the supervision include:

- Completion and updation of the research document;
- Signing of informed consent form;
- Source data verifciation;
- Distribution and management of the salt substitute;
- Stock in and stock out records for salt and other foods with high-salt content of various nursing homes;
- The implementation of blood potassium monitoring plan for hyperkalemic participants and participants in the high-emphasis monitoring group;
- Occurrence, handling and reporting of SAEs and PVs;

The project specialist will complete a written monitoring report after every visit. The monitoring report will be submitted to all parties involved in project management as required by the "Monitoring Plan".

11.4 Quality Control for Data

A project specialist from the PUCRI will review the completeness and accuracy of the CRF data that is recorded by the researchers according to the monitoringplan. He/she will also guide the staff member who input the data on revising or adding data based on the requirements. The data

management specialist from the PUCRI will conduct a logic check on the logical relationship of the CRF data, whether or not the data goes against the protocol, and whether or not it exceeds the normal range of reference. He/she will create a query table for incomplete, missing, or illogical data and request researchers to address, confirm, or correct the data in question. The database can only be locked and analyzed after all of the queries behind the data have been resolved.

12.Subject Protection

12.1 Ethics Review

This research protocol will be submitted to the PKU IRB for review and continuous review. It includes the completed research protocol, CRF, informed consent form, and other documents. The clinical trial can only be commenced after obtaining approval from the IRB. Throughout the implementation of the research, researchers shall abide by the ethical requirements of the GCP and the Declaration of Helsinki and guarantee the rights of the participants. We will regularly submit a research progress report as requested by the IRB and report the state of the research implementation in a timely manner.

12.2 Informed Consent

This research will seek informed consent from the managers of the nursing homes and all elderly staying in the nursing homes. The informed consent of the nursing homes will be obtained through a partnership agreement with the nursing home. Whether it is from the nursing home or the elderly, the researchers should give a detailed introduction on the purpose of the research, research methods, potential benefits and risks, and what kind of cooperation is expected from them when obtaining the informed consent form. The researchers should also tell them that participating in this clinical research is completely voluntary, that they can withdraw from the experiment at any time, and that refusing or withdrawing from the trial will not affect the treatment or reasonable medical rights of the elderly. The researchers have to address any questions that the other party might have during the process and give the elderly enough time to consider and discuss with their family members. The researchers should also confirm that the other party has decided on their own whether or not to participate in the research after they have a sufficient understanding of the research. Should the elderly be willing to participate in the research, they will be required to sign a hardcopy informed consent form. Illiterate elderly can use their fingerprints or appoint someone to sign on their behalf. The researchers who have obtained informed consent will also have to sign and state the date of signature on the informed consent form at the same time as the other party. The elderly will receive a copy of the informed consent

form. The original document will be appropriately kept by the researchers as part of the clinical research documents. Should there be a need to modify the protocol during the research implementation, or if new information is obtained that might influence the participants' continued participation in the research, the researchers have to make the appropriate modification to the informed consent form and seek informed consent from the participants again.

12.3 Confidentiality and Privacy

The personal information of all participants will be kept confidential and stored in a locked cabinet where the research materials are kept. Aside from members of the research group, only the supervisor from the Peking University Health Science Center and the PKU IRB has the right to read through the personal information of the participants. The participants' blood and urine samples will only be used for tests involving the research. Any leftover samples will be destroyed as biological samples on that day. Throughout the implementation of the research, all information of the participants will be marked using the participants' specific serial numbers or initials. The results of this research will be published in the form of a scientific paper, but all personal information of the participants (including their names, ages, etc.) will remain confidential.

This research will not look into the privacy of the participants. Participants can choose not to answer any questions they deem to be sensitive during the investigation.

13.Trial Management

This research belongs in the topic of "Efficacy, Safety and Health Economics Evaluation of Different Salt Reduction Strategies on Cardiovascular Diseases", under the "Research of Key Nutrition and Action Intervention Techniques and Strategies for Cardiovascular Diseases" of the 2016 National Key R&D Program of China. It is sponsored by the Ministry of Science and Technology of the PRC. Professor Yangfeng WU from the PUCRI has been appointed as the principal investigator. The PUCRI will be the organization leading the project and is responsible for designing the research protocol, technical training, quality control, data analysis, conclusion and publication; the partner organizations of this research include the Changzhi Medical College in Shanxi Province, School of Public Health of Xi'an Jiaotong University in Shaanxi Province, and the Disease Prevention and Control Center of Hohhot City in Inner Mongolia. These three organizations are responsible for supplying the research sites, selecting and managing the participants, implementing the intervention strategies, and collecting research data.

13.1 Advisory Committee

Chairman: Junshi CHEN

Members: Darwin Labarthe, Kiang Liu, Bruce Neal, Minghui ZHAO, Xingshan ZHAO, Wenyi NIU, Yanfang WANG, Hai FANG, Runlin GAO, Xiaofeng LIANG, Yong HUO, Changsheng MA, Jing YANG, Guansheng MA, Jun MA, Jianguo XU

13.2 Data and Safety Monitoring Boards

Chairperson: Mei WANG, Yihong SUN

Members: Luxia ZHANG, Wei ZHAO, Xian LI

13.3 Brief Introduction of sites

1. Changzhi Medical College

The Changzhi Medical College was included in the National Basic Ability Construction Project of Western and Central China in 2016. In the past five years, the college has undertaken 7 projects of the National Natural Science Foundation of China and 86 provincial-level scientific research projects. Personnel from the college have published 2907 essays, of which 239 were included in the SCI. They have also published 77 academic publications, 3 translated works, and 345 pieces of teaching material. The "Journal of Changzhi Medical College" sponsored by this college has exchanged knowledge on scientific publications with more than 100 colleges in the country.

The Changzhi Medical College has partnered with the principal investigator of this project, Professor Yangfeng WU, on many international projects and has a strong team of workers on the field. Personnel of the college have also participated in the fieldwork of the China Rural Health Initiative-Sodium Reduction Study (CHRI-SRS) in Shanxi Province. They were also responsible for the completion of all fieldworks for the "School-based education program to reduce salt intake in children and their families (School-EduSalt)". The organization is very experienced in on-site investigations and the implementation of salt reduction strategies, and is able to provide a good trial site for this project.

2. School of Public Health of XI'an Jiaotong University

The School of Public Health of XI'an Jiaotong University has tackled key science and technology problems of the country, supported various important specialized projects, undertaken projects from the Natural Science Foundation, tackled ministerial (provincial) problems, and partnered in more than 100 international projects. It has amassed more than 30 million yuan in research funds. It has also gotten 2 prizes of Klaus Schwarz awarded by the International Society of Biological Inorganic Chemistry, as well as more than 10 first and second prizes at the provincial level. Personnel from this school have published more than 2000 scientific papers, with over 400 of them included in the SCI.

The School of Public Health of XI'an Jiaotong University has partnered with the principal investigator of this project, Professor Yangfeng WU, on many international projects and has a strong team of workers on the field. They were also responsible for the fieldwork of the "China Salt

Substitute Study" (CSSS) and the CHRI-SRS in Shaanxi Province. The organization is very experienced in on-site investigations and the implementation of salt reduction strategies, and is able to provide a good trial site for this project.

3. Disease Prevention and Control Center of Hohhot City

The Disease Prevention and Control Center of Hohhot City is mainly responsible for the overall management on prevention and control of infectious disease in the Hohhot City, children immunization, implementation of control and monitoring strategies for disinfection and vector organisms, as well as the prevention, control, and technical guidance of chronic non-infectious diseases.

The Disease Prevention and Control Center of Hohhot City has been active in scientific research over the years and the level of their scientific research has been steadily increasing. They have once awarded the first prize of the Hohhot City Science and Technology Progress Award in 2008. They have a young and strong working team in the field of chronic disease prevention and control. This organization is experienced in field investigations and is able to provide a good trial site for the project.

4. Yangcheng Ophthalmology Hospital

The Yangcheng Ophthalmology Hospital is located in Jincheng City, Shanxi Province. It is a publicly-run specialist hospital. The hospital has well-equipped facilities and a good working relationship with organizations such as the Yangcheng Health Bureau, and can provide a good trial site for the project.

14. Storage of Research Documents and Records

The research organization must establish research documents and record files to ensure accurate reporting, explanation, and verification. These documents are classified into two areas: (1) Original documents which record clinical data such as changes in blood pressure and safety parameters. (2) Management files of the research organization, at least including the revised versions of research documents such as clinical research protocol, CRF, and informed consent forms, approval documents of the Ethics Committee, salt substitute management records, responsibility and authorization documents of the personnel of the research sites, training records, project progress plan and its updates, meeting minutes, etc.

The research organization has to store the above two categories of documents for at least 5 years after the project ends or is terminated. No research documents can be destroyed without the written approval of both the project manager from the PUCRI management office and the principal investigator.

Project management documents such as the supervision reports and progress summary that is made by the PUCRI must be stored at the PUCRI for at least five years after the project ends or is terminated.

15. References

1. Chen W-W, Gao R-L, Liu L-S, et al. China cardiovascular diseases report 2015: a summary. Journal of Geriatric Cardiology : JGC 2017; 14(1): 1-10.

2. Lawes CM, Vander Hoorn S, Rodgers A. Global burden of blood-pressure-related disease, 2001. The Lancet 2008; 371(9623): 1513-8.

3. Wu Y, Benjamin EJ, MacMahon S. Prevention and control of cardiovascular disease in the rapidly changing economy of China. Circulation 2016; 133(24): 2545-60.

4. From burden to "best buys": Reducing the economic impact of NCDs in low- and middle-income countries Executive summary 2011. http://www.who.int/nmh/publications/best_buys_summary/en/

5. Xinhuanet. The report on nutrition and chronic disease of Chinese residents published. http://wwwxinhuanetcom/live/20150630b/indexhtm 2015.

6. Chang HY, Hu YW, Yue CS, et al. Effect of potassium-enriched salt on cardiovascular mortality and medical expenses of elderly men. The American journal of clinical nutrition 2006; 83(6): 1289-96.

7. Group CSSSC. Salt substitution: a low-cost strategy for blood pressure control among rural Chinese. A randomized, controlled trial. Journal of hypertension 2007; 25(10): 2011-8.

8. Zhou B, Wang HL, Wang WL, Wu XM, Fu LY, Shi JP. Long-term effects of salt substitution on blood pressure in a rural north Chinese population. J Hum Hypertens 2013; 27(7): 427-33.

9. Zhao X, Yin X, Li X, et al. Using a low-sodium, high-potassium salt substitute to reduce blood pressure among Tibetans with high blood pressure: a patient-blinded randomized controlled trial. PLoS One 2014; 9(10): e110131.

10. 21 CFR 184.1622 - POTASSIUM CHLORIDE. https://www.gpo.gov/fdsys/search/pagedetails.action?collectionCode=CFR&browsePath=Title+21%2FChapter+I%2FSubchapter+B%2FPart+184%2FSubpart+B%2FSection+184.1622&granuleId=CFR-2010-title21-vol3-sec184-1622&packageId=CFR-2010-title21-vol3&collapse=true&fromBrowse=true

11. Losby JL, Patel MD, Schuldt MJ, Hunt MGS, Stracuzzi MJC, Johnston MY. Sodium-reduction strategies for meals prepared for older adults. Journal of public health management and practice: JPHMP 2014; 20(1 0 1): S23.

Summary of Changes in Protocol Amendments

**Document History**	
**Document**	**Date**
Amendment 3 (Version 1.4) used for IRB continued review	17 Oct 2019
Amendment 2 (Version 1.3) used for IRB continued review	16 Apr 2018
Amendment 1 (Version 1.2) used for IRB continued review	8 Dec 2017
Original Protocol (Version 1.1) used for IRB initial review	19 Sep 2017

Amendment 3, 17 Oct 2019: Final version of study protocol

The summary of changes provided here describe the major changes made from Original Protocol to Amendment 3, including detailed description of changes and the sections where the changes were made as well as the corresponding rationales.

Summary of Changes to the Protocol			
Date of amendments	Sections with amendments	Description of Change	Brief Rationale
Amendment 1, dated 8 Dec 2017	Section 3 Study Design	The number of eligible senior residential facilities enrolled in the study changed from 36 to 48. Adding Yangcheng county as the forth region. The number of regions of the study became 4 regions in Northern China.	One of the three regions could only recruit 8 facilities rather than 12 facilities that was originally planned (12 in each region). To ensure the study power and prevent any dropout in future years, the study decided to invite the fourth region to participate in the study and the total number of facilities were also increased.
	Section 4 Participants	Revised the inclusion criteria of eligible facilities. The least number of residents living in facilities changed from 30 to 20.	Improve the feasibility of the study so that more facilities could be eligible for recruitment.
	Section 5.4 Study Interventions	Revised the description of the study salt (regular salt or salt substitute) supply. All study salt was provided centrally free of cost, rather than	Due to the funding constraints, we tried and successfully got the support from the China Salt Yulin

		purchased by each facility.	Co, Ltd, to provide free study salt, both the salt substitute and regular salt, for free throughout the 2 years of intervention period. It should also help to enhance the cooperation from the facilities.
	Section 10 Sample Size	Revised the sample size. The number of clusters changed from 36 to 48. The number of participants in each facilities changed from 30 to 20. The corresponding sample size was changed from 1080 to 960.	Due to the increase in number of regions and facilities.
	Section 13 Management plan	Revised the description of the cooperative institution. Added Yangcheng Ophthalmology Hospital, Shanxi, as the forth study institution.	As given above, to increase resources for completion of the study.
Amendment 2, dated 16 Apr 2018	Section 7 Safety consideration	Detailed the safety monitoring plan for participants allocated to the salt substitute.	To intensify the safety monitoring plan to detect the possible cases of hyperkalemia that might be induced by the use of salt substitute that contains enriched potassium, and improve the protection of participant's safety.
Amendment 3, dated 17 Oct 2019	Section 7.4 Serious Adverse Event	Updated description of reporting of Serious Adverse Events. For expected and not relevant SAEs, do not need to follow the 'in-time reporting to IRB' rule, but record them and submit a summary to the IRB at the end of the study. For unexpected or study relevant SASs, still need to follow the 'in-time reporting to IRB' rule.	In response to the IRB's policy changes.

STATISTICAL ANALYSIS PLAN

Diet, ExerCIse and carDiovascular hEalth (DECIDE)–Salt Reduction Strategies for Seniors in Residential Facilities (DECIDE-Salt)

Version 1.0 April 19,2021

Drafted By: Yifang Yuan

Reviewed by: Pei Gao, Bruce Neal, Kiang Liu

Approved by: Yangfeng Wu

STATISTICAL ANALYSIS PLAN APPROVAL SHEET

Study: DECIDE-Salt

Version: 1.0

Version date: April 19, 2021

The undersigned have reviewed this plan and find it to be consistent with the requirements of the protocol as it applies to their respective areas.

Signature: 武阳丰 **Date** 2021-4-19

Yangfeng Wu, Project PI

Signature: [illegible] **Date** 2021-4-19

Pei Gao, Statistician

Signature: [illegible] **Date** 2021-4-19

Yifang Yuan, Data analyst

Table of Contents

List of abbreviations 5

1. Introduction 1

   1.1 Background and rationale 1

   1.2 Objectives 1

2. Study design 1

   2.1 Trial design 1

   2.2 Eligibility criteria 2

   2.3 Outcome definitions 2

   2.4 Randomization 3

   2.5 Sample size 3

   2.6 Framework 3

   2.7 Statistical interim analysis and stopping guidance 4

   2.8 Timing of final analysis 4

   2.9 Timing of outcome assessments 4

3. Statistical Principles 4

   3.1 Confidence intervals and p values 5

   3.2 Adherence and protocol deviations 5

   3.3 Analysis populations 5

   3.4 Missing data 6

   3.5 Outliers 7

4. Description of study population 7

   4.1 Study flow diagram 7

   4.2 Baseline characteristics 7

5. Analysis methods 8

   5.1 Primary outcome 8

      5.1.1 Primary analysis 8

      5.1.2 Secondary analysis 10

   5.2 Secondary outcomes 12

      5.2.1 Analysis for continuous secondary outcome 12

      5.2.2 Analysis for events 13

5.3 Analysis for safety outcome 13
5.3.1 safety outcomes 14
5.3.3 safety monitoring data 16
5.4 Cost-effective analysis 17
5.5 Analysis for adherence 17
5.6 Subgroup analysis 17
5.7 Sensitivity analysis 18
5.8 Statistical software 19
5.9 Revisions to protocol 20
6 Main tables and figures 20
6.1 Tables 20
6.2 Figures 25
7 References 28

List of abbreviations

Abbreviations	Definition
BMI	body mass index
BP	blood pressure
DBP	diastolic blood pressure
FAS	full-analysis set
HR	heart rate
LOCF	last observation carried forward
MI	multiple imputation
PPS	per-protocol analysis set
SAE	serious adverse events
SAS	safety set
SBP	systolic blood pressure
SS	salt substitute
SSSC	stepwise salt supply control

1. Introduction

1.1 Background and rationale

High sodium intake has been considered as the leading dietary risk factor for deaths and disability adjusted life-years among older adults. High-quality randomized trials to evaluate the effects of practical sodium reduction strategies are lacking.

1.2 Objectives

The primary aim of the present study is to determine the efficacy of two practical and scalable sodium reduction intervention strategies on systolic blood pressure (SBP) among older adults living in senior residential facilities: (1) a stepwise reduction in the supply of regular salt to institutional kitchens and (2) replacing regular salt with salt substitute. The null hypothesis is that neither of the two sodium reduction interventions will reduce SBP. And if the effect of both interventions do exist, the interaction between SS and SSSC in lowering SBP during study period will be further analyzed.

The secondary aims are to:

1) evaluate the safety of these strategies, particularly risks of hyperkalemia with salt substitute and hyponatremia in those receiving either or both interventions.
2) evaluate whether sodium reduction intervention can reduce the risk of cardiovascular events and death
3) assess the cost-effectiveness and adherence of sodium reduction strategies
4) assess the impact of sodium reduction intervention on microalbuminuria

2. Study design

2.1 Trial design

DECIDE-Salt is a 2-year 2 × 2 factorial, cluster randomized controlled trial testing the efficacy, safety, and cost-effectiveness of 2 intervention strategies for reducing dietary sodium intake

among older adults in residential facilities. The 2 interventions are (1) a controlled stepwise reduction of salt supply (SSSC) compared to “usual care (no reduction in salt supply)” and (2) introduction of low sodium salt substitute (SS) compared to regular salt. Each facility is randomly assigned to 1 of 4 groups: both SSSC and SS, SSSC only, SS only, and neither.

2.2 Eligibility criteria

Eligible participants were ≥55 years old at entry, expected to live in the facility for at least 2 years, and signed informed consent. Persons with physician-confirmed hyperkalemia, expected absence from the facility >1 month per year, or incomplete blood pressure measurement at the baseline were excluded.

The number of ineligible participants, if any, will be reported, with reasons for ineligibility

2.3 Outcome definitions

The primary outcome for the study is SBP assessed as continuous variable (in mmHg) evaluated during 24-month follow-up.

Secondary outcomes:

1) DBP assessed as continuous variable (in mmHg) evaluated during 24-month follow-up.
2) 24-hour urinary sodium (in mmol/d), potassium (in mmol/d), and microalbumin excretion (In mg/d) assessed as continuous variable evaluated during 24-month follow-up.
3) Incidence of hyperkalemia. A new case of hyperkalemia is defined as having serum potassium >5.5 mmol/L at either month 12 or 24 among participants with normal serum potassium at the baseline, regardless of clinical manifestations.
4) the risk of developing all-cause mortality, cardiovascular events (fatal and nonfatal stroke, fatal and nonfatal myocardial infarction, hospitalization or death caused by congestive heart failure)
5) ED-5Q during 24-month follow-up.

6) Incidence of renal dysfunction: A new case of renal dysfunction is defined as having eGFR <60 at either 12 or 24 months with normal eGFR at the baseline, regardless of clinical manifestations.
7) Incidence of hyponatremia: A new case of hyponatremia is defined as having serum sodium <135 mmol/L at either 12 or 24 months with normal serum sodium at the baseline, regardless of clinical manifestations.
8) A cost-effectiveness measure will be estimated based on the incremental cost of achieving each 1–mm Hg reduction in SBP

2.4 Randomization

The 48 eligible senior residential facilities (clusters) were randomized through a central computerized process in a 1:1:1:1 allocation ratio to the 4 intervention groups, with stratification by four regions, (Changzhi , Xi'an, Hohhot and Yangcheng). An independent statistician carried out the randomization after the baseline characteristics had been collected for study sites and individual participants. The allocation codes are kept by the statistician, and only the local staff responsible for the implementation of interventions have been informed. The outcome assessment team is independent of the staff responsible for implementing the interventions

2.5 Sample size

The study was designed to provide at least 80% power (with a 2-sided $\alpha = 0.05$) to detect a net reduction in mean SBP of at least 3.0 mm Hg between groups with either or both interventions compared to control. The power estimate assumed a standard deviation of systolic blood pressure of 18 mmHg based on previous studies, an intra-cluster correlation coefficient of 0.02, and a loss of follow-up rate of 20%, resulting in a sample size of 960 individuals drawn from 48 clusters, each with 20 participants.

2.6 Framework

Both primary and secondary aim are testing for difference.

2.7 Statistical interim analysis and stopping guidance

Interim analyses are not planned.

2.8 Timing of final analysis

The first main report of the trial will be prepared for the evaluation of efficacy and safety of sodium reduction intervention when every participant has reached 24 months follow-up and data for the primary endpoint has been received and cleaned (anticipated to be End of March, 2021).

2.9 Timing of outcome assessments

The schedule of the study procedure and the expected visit windows are given in **Table 1** as below.

TABLE 1 TIME-LINE FOR FOLLOW-UP

Time-point / Items	Preparation	Baseline	Follow-up						Quit
			6-month	12-month	18-month	24-month	Whenever necessary	Referral	
Informed consent	√								
Eligible criteria confirmation	√								
Basic information	√								
Demographics		√							
Lifestyle		√							
Diet survey		√	√	√	√	√			√
Past history		√							
EQ-5D		√		√		√			√
Anthropometrics		√				√			√
Vital signs		√	√	√	√	√			√
Complete blood count		√				√			√
Glucose and lipid panel		√				√			√
24-hour urine		√				√			√
Ophthalmoscopy		√				√			√
Antihypertensive meds		√	√	√	√	√			√
Hospitalization information		√						√	
Adverse event							√		
Signature page						√			√

3. Statistical Principles

The analysis will be conducted by intention-to-treat.

3.1 Confidence intervals and p values

All applicable statistical tests will be 2-sided and will be performed using a 5% significance level. All confidence intervals presented will be 95% and two-sided.

3.2 Adherence and protocol deviations

Compliance is assessed mainly based on the number of facilities that have adhered to the protocol throughout the study period. Other information that helps to assess compliance includes: 1) the 24hr sodium and potassium excretion; 2) site monitoring data on the 3-monthly checking on the salt supply, in against to the goal of consumption set by the interventionist according to the study plan; 3) degree of satisfaction with food prepared at the facilities. Descriptive statistics on the percent of compliant facility (N (%)) will be summarized.

A protocol deviation is defined as a failure to adhere to the protocol. The following are pre-specified definition of protocol violations:

1) For all facilities: dropping out of the study before the end of trial
2) Failure to adhere to intervention as planned:
    a. For facilities assigned to salt substitute intervention: stopping using the salt provided by researchers before the end of trial
    b. For facilities assigned to stepwise salt reduction strategy intervention: refusal to comply with the stepwise strategy

3.3 Analysis populations

Full-analysis set (FAS):

For the analysis of the primary outcome (SBP), FAS will include all eligible randomized subjects with baseline SBP measured and at least 1 post-baseline measurement on SBP, according to the intervention they were randomized to receive. No imputation will be used for primary outcome in main efficacy analysis.

For the analysis of clinical events, FAS will include all eligible randomized subjects with baseline SBP measured, follow-up SBP measurements are not required.

Per-protocol analysis set (PPS):

PPS consists all subjects who: 1) meet the above criteria for FAS 2) do not experienced major protocol deviation during the study period.

Safety set (SAS):

SAS will consist of all randomized subjects who: 1) have baseline serum potassium measured 2) have received at least one month of study intervention, regardless of their eligibility. Subject will be analyzed according to the intervention they actually received.

3.4 Missing data

No imputation will be conducted for primary analysis of primary outcome, secondary outcomes and safety outcomes.

For survival analysis, those who loses to follow-up will be regarded as right-censored and the censoring date will be the date of last encounter. If the date of occurrence of an event is not recorded, it will be imputed as the median of the date of last follow-up and the one when event reports.

For sensitivity analysis of SBP difference from baseline to 24-month SBP, 24-month SBP will be imputed using multiple imputation. The model will be:

$$Y_i = \beta_0 + \sum \beta_i X_i + e_i$$

$$e_i \sim N(0, \sigma_e^2)$$

Where Y_i is the SBP for subject i at month 24; β_0 is the intercept; X_i is the variable for imputation; e_i is the random error.

Variables for multiple imputation of 24-month SBP includes SBP, DBP and pulse at baseline, 6 months, 12 months and 18 months, as well as residential facilities, center, age, gender, education, BMI, past history of hypertension, diabetes and coronary artery disease, lifestyle information, medication use in baseline and follow-up.

3.5 Outliers

There is a separate procedure for the data management and queries solving in the DECIDE-Salt study. All data will be cleaned and datasets be closed formally before starting the statistical analyses described in this document. Thus, no more specific procedures for outliers in main outcomes will be conducted.

4. Description of study population

4.1 Study flow diagram

The "CONSORT" diagram comprising the total number, number of people screened, eligible, consented, randomized, receiving their allocated intervention, withdrawing/lost to follow-up will be used for summarization.

The number of ineligible participants, if any, will be reported, with reasons for ineligibility. The reasons for lose to follow-up over the course of the trial will be classified as "death", "hospitalization"," temporary leave of absence", "quit", "else".

The study flow chart will be represented in **Figure 1**.

4.2 Baseline characteristics

Participants will be described with respect to demographic information (age, gender, race, education), anthropometrics (weight, height, BMI), physical examination (SBP, DBP, HR), life style and diet habits, labs (serum potassium, renal function, urinary electrolytes), comorbidities, medication history at baseline, both overall and separately for the intervention and control groups.

Categorical data will be summarized by numbers and percentages. Continuous data will be summarized by mean, SD and range if data are normal and median, IQR and range if data are skewed. Minimum and maximum values will also be presented for continuous data.

Baseline characteristics will be described in **Table 1.**

5. Analysis methods

5.1 Primary outcome

The primary outcome is SBP evaluated during 24-month follow-up, to be compared between intervention groups.

5.1.1 Primary analysis

The primary analysis will be performed in FAS dataset. The results will be regarded as the main findings.

A pre-specified linear mixed effects model will be performed to model repeated measures of SBP of the same individual over time. The model will include intervention status and baseline SBP as fixed effects, as well as cluster and time as random effects. Separated models will be built for each intervention, either SS alone or SSSC alone. The model would be:

$$y_{ijt} = \beta_0 + \mu_{1i} + \mu_{2j} + \beta_1 x_{ij} + \beta_2 c_{ij} + \beta_3 t + e_{ijt}$$

$$e_{ijt} \sim N(0, \sigma_e^2)$$

$$\mu_{1i} \sim N\left(0, \sigma_{\mu 1}^2\right)$$

$$\mu_{2j} \sim N(0, \sigma_{\mu 2}^2)$$

Where *i* represents the ith subjects, *j* represents the jth cluster (facility), *t* represents time point t, y_{ijt} is the SBP measured for subject *i* in cluster *j* at time *t* (follow-up month), baseline SBP will not be included in y_{ijt} ; β_0 is the mean outcome in the control group at baseline; μ_{1i} is an individual random effect to allow for multiple repeats per person; μ_{2j} is a cluster random effect on baseline SBP to allow for between-cluster heterogeneity ; x_{ij} , the predictor of interest, either SS or SSSC, is an indicator of the intervention for subject i in cluster j (1 indicates receive intervention while 0 indicates not receive intervention); β_1 , the coefficient of interest , is the effect coefficient of intervention; c_{ij} is the baseline SBP measured for subject i in cluster j ; e_{ijt} is the random error for the measurement of subject i in cluster j at time t.

The estimation of β_1 , its 95% confidence interval and the corresponding p value will be reported in **Table 2.**

We will also evaluate the interaction between SS and SSSC in lowering SBP during study period. If the effect of both interventions do exist, we will add interaction term in the above model to test the interaction between SS and SSSC. The model would be:

$$y_{ijt} = \beta_0 + \mu_{1i} + \mu_{2j} + \beta_1 x_{1ij} + \beta_2 x_{2ij} + \beta_3 x_{1ij} * x_{2ij} + \beta_4 c_{ij} + \beta_5 t + e_{ijt}$$

$$e_{ijt} \sim N(0, \sigma_e^2)$$

$$\mu_{1i} \sim N(0, \sigma_{\mu 1}^2)$$

$$\mu_{2j} \sim N(0, \sigma_{\mu 2}^2)$$

Where *i* represents the ith subjects, *j* represents the jth cluster (facility), *t* represents time point t, y_{ijt} is the SBP measured for subject *i* in cluster *j* at time *t* (follow-up month*)* , baseline SBP will not be included in y_{ijt}; β_0 is the mean outcome in the control group at baseline; ; μ_{1i} is an individual random effect to allow for multiple repeats per person; μ_{2j} is a cluster random effect

on baseline SBP to allow for between-cluster heterogeneity ; x_{1ij} , one of the predictors of interest, is an indicator of the intervention SS for subject i in cluster j (1 indicates receive SS while 0 indicates not receive SS); β_1 , the coefficient of interest , is the effect coefficient of SS; x_{2ij} , one of the predictors of interest, is an indicator of the intervention SSSC for subject i in cluster j (1 indicates receive SSSC while 0 indicates not receive SSSC); β_2 , the coefficient of interest , is the effect coefficient of SSSC; β_3 is the effect coefficient of SS and SSSC interaction; c_{ij} is the baseline SBP measured for subject i in cluster j; e_{ijt} is the random error for the measurement of subject i in cluster j at time t.

The estimation of β_1 , β_2 , β_3 and their 95% confidence interval and the corresponding p value will be reported in **Table 2.**

We will not adjust for the multiple tests, i.e. we will test both primary aims at p=0.05, because the efficacy and effectiveness of salt substitute in lowering blood pressure has been proved in previous studies with RCT design and our current study on this specific intervention is considered an implementation study, which will inform us the effectiveness of applying the particular intervention in this particular setting, senior residential facilities. Only the SSSC is considered a novel intervention that has never been rigorously tested before.

.

5.1.2 Secondary analysis

Various secondary analysis will be conducted to examine the robustness of the conclusions of primary analysis.

5.1.2.1 Controlling for potential confounders

We will test if there is any imbalance between groups in baseline variables. We will additionally include the covariates that differs significantly at the baseline (p<0.05) to adjust confounding.

The models would be:

$$y_{ijt} = \beta_0 + \mu_{1i} + \mu_{2j} + \beta_1 x_{ij} + \beta_2 c_{ij} + \sum BZ_{ij} + \beta_3 t + e_{ijt}$$

$$e_{ijt} \sim N(0, \sigma_e^2)$$

$$\mu_{1i} \sim N\left(0, \sigma_{\mu 1}^2\right)$$

$$\mu_{2j} \sim N\left(0, \sigma_{\mu 2}^2\right)$$

Where i represents the ith subjects, j represents the jth cluster (facility), t represents time point t, y_{ijt} is the SBP measured for subject i in cluster j at time t (follow-up month), baseline SBP will not be included in y_{ijt} ; β_0 is the mean outcome in the control group at baseline; μ_{1i} is an individual random effect to allow for multiple repeats per person; μ_{2j} is a cluster random effect on individual baseline SBP to allow for between-cluster heterogeneity ; x_{ij} , the predictor of interest, either SS or SSSC, is an indicator of the intervention for subject i in cluster j (1 indicates receive intervention while 0 indicates not receive intervention); β_1 , the coefficient of interest , is the effect coefficient of intervention; c_{ij} is the baseline SBP measured for subject i in cluster j ; Z_{ij} is the baseline covariate other than SBP measured for subject i in cluster j ; e_{ijt} is the random error for the measurement of subject i in cluster j at time t.

If the interaction effect in primary analysis exists, then below model will be built for adjustment of covariates:

$$y_{ijt} = \beta_0 + \mu_{1i} + \mu_{2j} + \beta_1 x_{1ij} + \beta_2 x_{2ij} + \beta_3 x_{1ij} * x_{2ij} + \beta_4 c_{ij} + \sum BZ_{ij} + +\beta_5 t + e_{ijt}$$

$$e_{ijt} \sim N(0, \sigma_e^2)$$

$$\mu_{1i} \sim N\left(0, \sigma_{\mu 1}^2\right)$$

$$\mu_{2j} \sim N\left(0, \sigma_{\mu 2}^2\right)$$

Where i represents the ith subjects, j represents the jth cluster (facility), t represents time point t, y_{ijt} is the SBP measured for subject i in cluster j at time t (follow-up month) , baseline SBP will not be included in y_{ijt}; β_0 is the mean outcome in the control group at baseline; ; μ_{1i} is an individual random effect to allow for multiple repeats per person; μ_{2j} is a cluster random effect on individual baseline SBP to allow for between-cluster heterogeneity ; x_{1ij} , one of the predictors of interest, is an indicator of the intervention for subject i in cluster j (1 indicates receive SS while 0 indicates not receive SS); β_1 , the coefficient of interest , is the effect coefficient of SS; x_{2ij} , one of the predictors of interest, is an indicator of the intervention for subject i in cluster j (1 indicates receive SSSC while 0 indicates not receive SSSC); β_2 , the coefficient of interest , is the effect coefficient of SSSC; β_3 is the effect coefficient of SS and SSSC interaction; c_{ij} is the baseline SBP measured for subject i in cluster j; Z_{ij} is the baseline covariate other than SBP measured for subject i in cluster j; e_{ijt} is the random error for the measurement of subject i in cluster j at time t.

These results will be presented in **Table 3.**

5.1.2.2 Analysis in PPS

The per protocol analysis will be conducted in PPS. The results will be presented in **Table 3**.

5.2 Secondary outcomes

Secondary outcome for the effectiveness of intervention includes DBP, 24h urinary sodium, 24h urinary potassium and microalbumin, EQ-5D and events.

5.2.1 Analysis for continuous secondary outcome

For continuous secondary outcomes we will use the same models as that for the primary outcome to test the difference between intervention groups.

If the distribution of the outcome variable is normal or mild skew, no transformation will be applied. If the outcome variable is heavily right-skewed, a log transformation will be conducted.

If the outcome variable is heavily left-skewed, a power transformation (e.g. box-cox transformation) will be conducted.

Results for continuous secondary outcomes will be reported in **Table 2** and **Table 3**.

5.2.2 Analysis for events

Frailty survival models (cox proportional hazards model with mixed effects) that account for within-cluster homogeneity will be used for the analysis of all-cause mortality, cardiovascular events (fatal and nonfatal stroke, fatal and nonfatal myocardial infarction, hospitalization or death caused by congestive heart failure). The proportional hazards assumption will be confirmed using statistics and graphs based on the Schoenfeld residuals. If more than one events took place during the study period, only the first event will be counted. Separated models will be built for each intervention, either SS alone or SSSC alone. The cluster(facility) will be regarded as the random effect. The models would be:

$$h_{ij}(t) = h_0(t)\exp(\beta_1 x_{ij} + \mu_j)$$

Where $h_{ij}(t)$ is the hazard function for the ith subject in jth cluster. $h_0(t)$ denotes the baseline hazard function. ; x_{ij} , the predictor of interest, either SS alone or SSSC alone, is an indicator of the intervention for subject i in cluster j (1 indicates receive intervention while 0 indicates not receive intervention); β_1 , the coefficient of interest , is the effect coefficient of intervention; μ_j denotes the random effect associated with the jth cluster. An additional multivariate model will be conducted to controlling for the potential unbalanced covariates at baseline, if any.

The results for event will be presented in **Table 4** and **Table 5**.

5.3 Analysis for safety outcome

All analysis for safety outcomes will be conducted on safety dataset.

5.3.1 safety outcomes

The safety outcomes include:

1) Incidence of hyperkalemia;
2) Incidence of renal dysfunction;
3) Incidence of hyponatremia;
4) Mean serum potassium and sodium during follow-up

5.3.2 Randomized comparisons

5.3.2.1 Incidence of hyperkalemia

Only participants without hyperkalemia at baseline will be included in the study. A generalized linear mixed model accounting for clustering will be used for incidence of hyperkalemia

1) To evaluate the effect of SS or SSSC alone, the model will be:

The model will include intervention status as fixed effects, as well as cluster as random effects. Thus, the model would be:

$$Logit(\ P(y_{ij} = 1)\ ) = \ \beta_0 + \mu_j + \beta_1 x_{ij} + e_{ij}$$

$$e_{ij} \sim N(0, \sigma_e^2)$$

$$\mu_j \sim N\left(0, \sigma_{\mu j}^2\right)$$

Where *i* represents the ith subjects, *j* represents the jth cluster (facility), y_{ij} is the probability of hyperkalemia for subject *i* in cluster *j*; β_0 is the mean outcome in the control group; ; μ_j is a cluster random effect to allow for between-cluster heterogeneity ; x_{ij} , the predictor of interest, either SS or SSSC, is an indicator of the intervention for subject i in cluster j (1 indicates receive intervention while 0 indicates not receive intervention), β_1 , the coefficient of interest, is the effect coefficient of intervention; e_{ij} is the random error for the measurement of subject i in cluster j.

2) To test the interaction effect of SS and SSSC, the model will be:

The model will include intervention status as fixed effects, as well as cluster as random effects. Thus, the model would be:

$$Logit(P(y_{ij} = 1)) = \beta_0 + \mu_j + \beta_1 x_{1ij} + \beta_2 x_{2ij} + \beta_3 x_{1ij} * x_{2ij} + e_{ij}$$

$$e_{ij} \sim N(0, \sigma_e^2)$$

$$\mu_j \sim N(0, \sigma_{\mu j}^2)$$

Where i represents the ith subjects, j represents the jth cluster (facility), y_{ij} is the probability of hyperkalemia for subject i in cluster j; β_0 is the mean outcome in the control group; ; μ_j is a cluster random effect to allow for between-cluster heterogeneity ; x_{1ij} is an indicator of the SS for subject i in cluster j (1 indicates receive intervention while 0 indicates not receive intervention), β_1 is the effect coefficient of intervention; x_{2ij} is an indicator of the SSSC for subject i in cluster j (1 indicates receive intervention while 0 indicates not receive intervention), β_2 is the effect coefficient of intervention; β_3 is the coefficient for interaction between SS and SSSC; e_{ij} is the random error for the measurement of subject i in cluster j.

Secondary analysis, with adjustment for unhealthy conditions [2] included hypertension, diabetes mellitus, coronary heart disease, stroke, chronic kidney disease, cancer, chronic obstructive pulmonary disease, being bedridden, or eGFR < 60 mL/min/1.73m2 will be conducted for both aims above.

5.3.2.2 incidence of renal dysfunction

Analysis of incidence of renal dysfunction will follow the same approaches as that for incidence of hyperkalemia.

5.3.2.3 incidence of hyponatremia

Analysis of incidence of renal dysfunction will follow the same approaches as that for incidence of hyperkalemia.

5.3.2.4 Serum potassium and sodium

Both descriptive and model analyses will follow the same approaches as that for the efficacy analyses for primary outcome.

These results will be reported in **Table 6** and **Table 7**

5.3.3 safety monitoring data

A monitoring plan was conducted in participants allocated to salt substitute to detect possible hyperkalemia due to the use of salt substitute. This includes additional assessment of serum potassium and self-reported questionnaires regarding symptoms of hyperkalemia and medication use at 3- and 6- month. All salt substitute recipients are required to have at least 1 blood assay within 6 months of starting intervention.

Thus, the analysis of safety monitoring data will be performed only on safety dataset in SS group. The data includes serum potassium tests and hyperkalemia questionnaire at month 3, month 6 in SS group.

Below variables will be analyzed:

1) incidence of hyperkalemia.
2) Serum potassium
3) incidence of positive result for hyperkalemia questionnaire

We will list the number of participants with hyperkalemia identified by the monitoring plan in each screening and the results of re-test of serum potassium. We will particularly focus on the trend with time in the number of patients with occasional and persistent abnormal serum potassium.

5.4 Cost-effective analysis

This analysis will be performed in FAS set. A cost-effectiveness measure will be estimated based on the incremental cost of achieving each 1–mm Hg reduction in SBP. The cost is the fee (unit: Yuan)associated with intervention, including cost for salt substitute, faculty training, health education, diagnose and treatment for hyperkalemia. Incremental cost-effective ratio (ICER) will be calculated as below:

$$IC = Cost_{initial} - Cost_{complete}$$

$$IE = SBP_{initial} - SBP_{complete}$$

$$ICER = IC/IE$$

5.5 Analysis for adherence

Compliance is assessed based on 1) the number of facilities that have adhered to the study protocol throughout the study period 2) the 24hr urinary sodium and potassium excretion; 3) site monitoring data on the 3-monthly checking on the salt supply, in against to the goal of consumption set by the interventionist according to the study plan;

Descriptive statistics on the percent of compliant facility (N (%)) will be summarized.

For urinary electrolytes, both descriptive and model analyses will follow the same approaches as that for the efficacy analyses according to variable's math character, categorical or continuous.

For salt supply monitoring data, descriptive statistics on the percent compliance (N (%)) will be summarized in total and by each region.

5.6 Subgroup analysis

The following pre-specified subgroup analyses will be performed on the primary outcomes and safety outcomes stratified by:

1) baseline blood pressure:
    a. normal: baseline SBP < 140mmHg and DBP < 90mmHg
    b. stage 1: baseline BP 140-159/90-99mmHg
    c. stage 2 or above: baseline SBP > 160mmHg or DBP > 100mmHg
2) hypertension status:
    a. normotensive: baseline BP < 140/90mmHg and not taking antihypertensive medication within 2 weeks before baseline
    b. hypertensive: had baseline SBP ≥ 140mm Hg or DBP ≥ 90mmHg, or on antihypertensive medications within 2 weeks before baseline

3) region:
    a. Changzhi
    b. Xi'an
    c. Hohhot
    d. Yangcheng
4) Sex:
    a. Male
    b. Female
5) Age
    a. < 70 years
    b. ≥ 70 years

    Using 70 years as the cut-off is because the mean age at the baseline was about 71 years.

6) educational attainment
    a. illiterate, semi-illiterate or completed primary school
    b. completed junior high school or above

Results will be presented on forest plots in **Figure 3** with the interaction results alongside

5.7 Sensitivity analysis

Sensitivity analysis will be performed as below:

1) the per-protocol analysis of outcomes
2) to assess the effect of intervention on SBP change from baseline to 24-month, we will first use multiple imputation to impute missing value at 24-month. Then we will perform mixed model to account for clustering. The model would be:

$$y_{ij} = \beta_0 + \mu_j + \beta_1 x_{ij} + \beta_2 c_{ij} + \sum BZ_{ij} + e_{ij}$$

$$e_{ij} \sim N(0, \sigma_e^2)$$

$$\mu_j \sim N\left(0, \sigma_{\mu j}^2\right)$$

Where i represents the ith subjects, j represents the jth cluster (aka. facility), y_{ij} is the SBP difference measured for subject i in cluster j ; β_0 is the mean outcome in the control group at baseline; μ_j is a cluster random effect to allow for between-cluster heterogeneity; x_{ij} is an indicator of the intervention for subject i in cluster j (1 indicates receive intervention while indicates not receive intervention), β_1 is the effect coefficient of intervention; c_{ij} is the baseline SBP measured for subject i in cluster j; Z_{ij} is the covariates other than baseline SBP; e_{ij} is the random error for the measurement of subject i in cluster j.

3) Since it may take several months for SSSC to take effect, we will conduct a sensitivity analysis for SSSC after excluding data in 6-month.
4) Since more missing data was observed in Xi'an, we will perform a sensitivity analysis after excluding participants from Xi'an.
5) For the analysis of urine electrolytes, a sensitivity analysis will be conducted after excluding possibly incomplete 24h urine collection. Incomplete 24h urine sample is defined as urine volume < 500ml/d.
6) For the analysis of events, a sensitivity analysis will be conducted on all randomized participants, regardless of the availability of the baseline BP measurements.

5.8 Statistical software

The analysis will be carried out using SAS version 9.4. Other software such as R, Stata or python may be used if necessary.

5.9 Revisions to protocol

Protocol	Changes in SAP	Justification
The primary outcome for the study was the change in SBP from baseline to 2-year follow-up.<br><br>The primary analysis of intervention effects will use a generalized estimating equation with an exchangeable covariance structure accounting for clustering and adjustment for baseline SBP.	The primary outcome for the study is changed to SBP evaluated during 24-month follow-up.<br><br>A pre-specified linear mixed effects model will be performed to model repeated measures of SBP of the same individual over time.	To account for the impact of missing data due to high mortality and leaving from the residential facilities, all available follow-up data in SBP will be used instead of that just in 24-month alone. Thus, linear mixed model, which naturally fits to hierarchical data, is more appropriate than GEE in such situation.
Continuous secondary outcomes were changes in diastolic blood pressure; serum potassium; and 24-hour urinary sodium, potassium, and microalbumin excretion.	Like the primary outcome SBP, all continuous secondary outcomes are change to the mean level evaluated during 24-month follow-up	Same as above.
	Add secondary outcomes: Incidence of renal dysfunction: A new case of renal dysfunction is defined as having eGFR <60 at either 12 or 24 months, regardless of clinical manifestations.	We are particularly interested in the safety concerns about salt reduction strategies. Renal dysfunction is a potential side effect of salt reduction.

6 Main tables and figures

6.1 Tables

Table 1. Baseline Characteristics of study population

	All<br>N =	SS+SSSC<br>N =	SSSC<br>N =	SS<br>N =	control<br>N =	p value
**Cluster level**						
Number of senior residential facilities, n						
Number of study participants per facility, median (IQR)						
**Individual level**						
**Demographics and anthropometrics**						
Age, mean(SD)						

Male, n (%)
Number of participants by center, n (%)
Changzhi
Xi'an
Hohhot
Yangcheng
Education level, n (%)
Primary school and below
Secondary school
High school
Undergraduate & above
BMI, mean (SD)
**Life style**
Smoking status, n (%)
Current smokers
Ex-smoker
Never-smokers
Alcohol intake, n (%)
Yes
No
**Comorbidities**
Hypertension, n (%)
Yes
No
Coronary artery disease, n (%)
Yes
No
Diabetes, n (%)
Yes
No
Stroke, n (%)
Yes
No
Renal disease, n (%)
Yes
No
**Medication use**
Antihypertensive medication, n (%)
Yes
No
Potassium-sparing medications, n (%)
Yes
No

**Vital signs**	
SBP, mean(SD)	
DBP, mean(SD)	
Pulse, mean(SD)	
**Labs**	
Total cholesterol, mean(SD)	
LDL-C, mean(SD)	
Triglycerides, median(IQR)	
HDL-C, mean(SD)	
Fasting glucose, mean(SD)	
Serum potassium, mean(SD)	
Serum sodium, mean(SD)	
eGFR, mean(SD)	
Urinary potassium, mean(SD)	
Urinary sodium, mean(SD)	
Urinary creatinine, mean(SD)	
Urine volume, median(IQR)	

Table 2. The effect of intervention on changes of primary outcome and secondary outcomes

	SS		SSSC		p for interaction
	Difference of outcome (95%CI)	p value	Difference of outcome (95%CI)	p value	
**Primary outcome**					
SBP,mmHg					
**Secondary outcome**					
DBP,mmHg					
urinary sodium,mmol/d					
urinary potassium,mmol/d					
microalbumin, mg/d					
serum potassium,mmol/l					
serum sodium,mmol/l					

Table 3. The effect of intervention on adjusted changes of primary outcome and secondary outcomes

	SS		SSSC		p for interaction
	Difference of outcome (95%CI)	p value	Difference of outcome (95%CI)	p value	
**Primary outcome**					
SBP,mmHg					

**Secondary outcome**	
DBP,mmHg	
urinary sodium,mmol/d	
urinary potassium,mmol/d	
microalbumin, mg/d	
serum potassium,mmol/l	
serum sodium,mmol/l	

Models were adjusted for

Table 4. Relative risk for all-cause mortality and cardiovascular events

Total N	SS			SSSC			p for interaction
N(%)	N(%)	Hazard ratio (95%CI)	p value	N(%)	Hazard ratio (95%CI)	p value	
All-cause mortality							
Cardiovascular events							

Table 5. Adjusted relative risk for all-cause mortality and cardiovascular events

Total N	SS			SSSC			p for interaction
N(%)	N(%)	Hazard ratio (95%CI)	p value	N(%)	Hazard ratio (95%CI)	p value	
All-cause mortality							
Cardiovascular events							

Models were adjusted for

Table 6. Relative risk for safety outcomes

	Total N	SS			SSSC			p for interaction
	N(%)	N(%)	Relative risk (95%CI)	p value	N(%)	Relative risk (95%CI)	p value	
Incidence of hyperkalemia								
Incidence of renal dysfunction								
Incidence of hyponatremia								

Table 7. Adjusted relative risk for safety outcomes

	Total N	SS	SSSC

	N(%)	N(%)	Relative risk (95%CI)	p value	N(%)	Relative risk (95%CI)	p value	p for interaction
Incidence of hyperkalemia								
Incidence of renal dysfunction								
Incidence of hyponatremia								

Models were adjusted for

6.2 Figures

Figure 1. Flow diagram

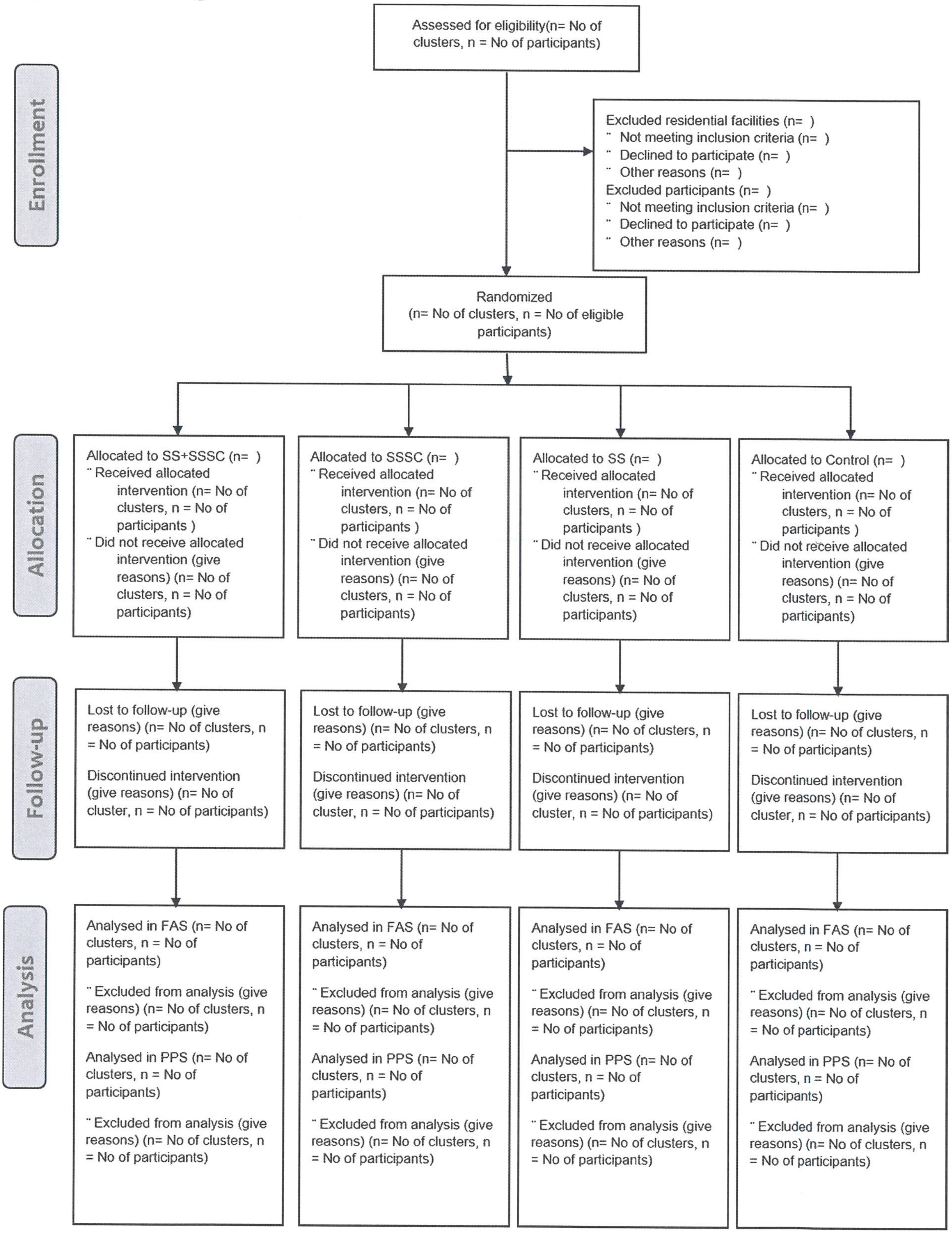

Figure 2. Trends in mean SBP and DBP and their respective 95%CI by intervention and control group

Mean SBP and DBP and their respective 95%CI in baseline, 6-month, 12-month, 18-month and 24-month will be plotted by SSSC + SS, SSSC, SS and control group

A. SBP

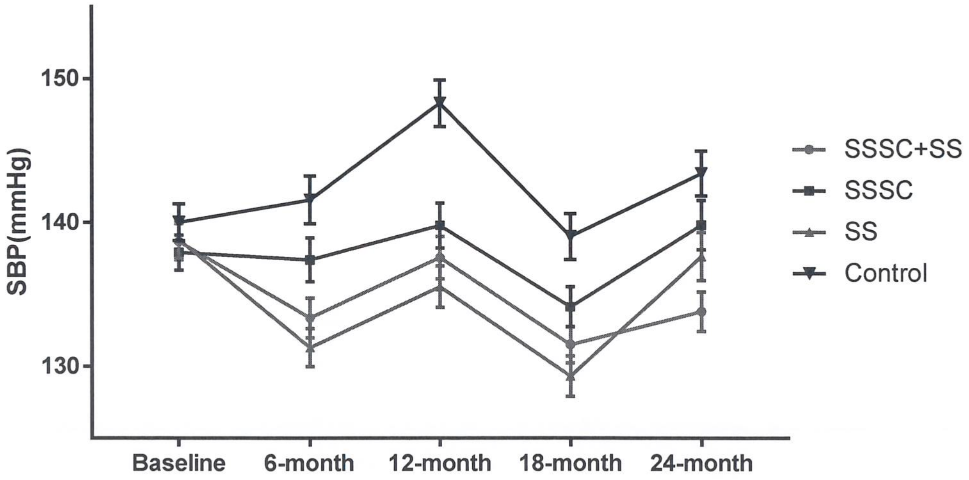

B. DBP

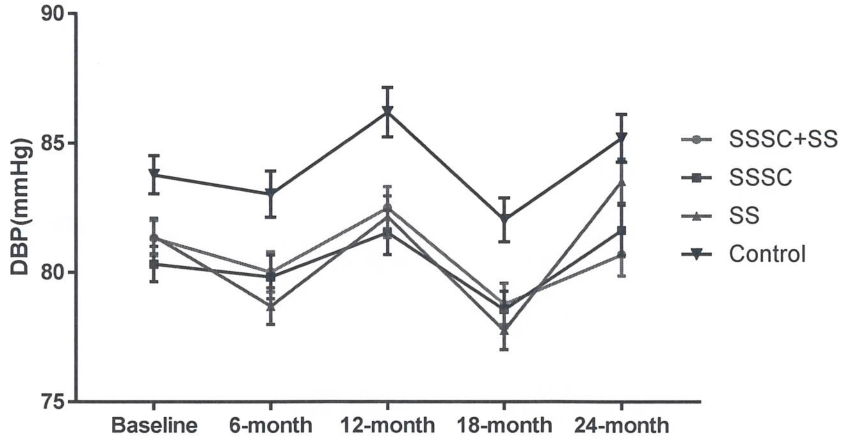

Figure 3. Forest plot for subgroup analysis

Results from subgroup analysis by age, sex, region, educational attainment, baseline BP, baseline hypertensive status and baseline antihypertensive medication use will be summarized by forest plot.

A. For SSSC

	SSSC			non-SSSC			Coefficient (95%CI)of intervention (Forest plots)	P for interaction
	Total	mean	SD	Total	mean	SD		
**Overall**								
**Region**								
Changzhi								
Xi'an								
Hohhot								
Yangcheng								
**Age, years**								
< 65								
>= 65								
**Sex**								
Male								
Female								
**Education**								
illiterate, semi-illiterate or completed primary school								
completed junior high school or above								
**Baseline BP**								
Normal								
Stage 1								
Stage 2 or above								
**Hypertension status**								
Normotensive								
Hypertensive								
**Antihypertensive medication**								
No potassium-sparing medications								
Potassium-sparing medication use								

0

Favors SSSC   Favors non-SSSC

B. For SS

	SS			non-SS			Coefficient (95%CI)of intervention (Forest plots)	P for interaction
	Total	mean	SD	Total	mean	SD		
**Overall**								
**Region**								
Changzhi								
Xi'an								
Hohhot								
Yangcheng								
**Age, years**								
< 65								
>= 65								
**Sex**								
Male								
Female								
**Education**								
illiterate, semi-illiterate or completed primary school								
completed junior high school or above								
**Baseline BP**								
Normal								
Stage 1								
Stage 2 or above								
**Hypertension status**								
Normotensive								
Hypertensive								
**Antihypertensive medication**								
No potassium-sparing medications								
Potassium-sparing medication use								
							0<br>Favors SS / Favors non-SS	

7 References

1. Gamble C, Krishan A, Stocken D, et al. Guidelines for the Content of Statistical Analysis Plans in Clinical Trials. *JAMA*. 2017;318(23):2337-2343. doi:10.1001/jama.2017.18556
2. Jin A, Zhao M, Sun Y, et al. Normal range of serum potassium, prevalence of dyskalaemia and associated factors in Chinese older adults: a cross-sectional study. BMJ Open 2020;10:e039472. doi: 10.1136/bmjopen-2020-039472
