# Supplementary material for: Cost-Effectiveness of Salt Substitute and Salt Supply Restriction in Eldercare Facilities: The DECIDE-Salt Cluster Randomized Clinical Trial
Source: JAMA Netw Open. 2024 Feb 12;7(2):e2355564. doi: 10.1001/jamanetworkopen.2023.55564 (PMC10862151; doi:10.1001/jamanetworkopen.2023.55564)
Supplement: Supplement 2. — eFigure 1. Flow Chart eTable 1. Questionnaires to Obtain Facility-Level and Provincial-Level Cost Indicators at Different Stages of Intervention eTable 2. Baseline Characteristics of Study Participants by Randomized Comparisons (n = 1612) eTable 3. Comparisons in Baseline Characteristics Between Participants Analyzed and Not Analyzed for the Assessment of Effect on Blood Pressure Among 1612 Eligible Participants eTable 4. Estimated MACE Treatment Cost for Each Participant at Different Stages of Intervention eTable 5. Estimated Changes in Antihypertension Medication Cost for Each Participant at Different Stages of Intervention eTable 6. Health Indicators for Study Participants at Different Stages of Intervention eTable 7. Economic Evaluation Results With ICER and ICUR Values eFigure 2. Probabilistic Sensitivity Analysis of the Restricted Supply Intervention at 2 Years Using Monte Carlo Simulation eTable 8. Projected Health Benefits and Costs When Salt Substitute Is Rolled Up Through All Eldercare Facilities in China [file jamanetwopen-e2355564-s002.pdf]

Supplemental Online Content

Lai X, Yuan Y, Wang H, et al; DECIDE-Salt Research Group. Cost-effectiveness of salt substitute and salt supply restriction in eldercare facilities: the DECIDE-Salt cluster randomized clinical trial. *JAMA Netw Open*. 2024;7(2):e2355564. doi:10.1001/jamanetworkopen.2023.55564

eFigure 1. Flow Chart

eTable 1. Questionnaires to Obtain Facility-Level and Provincial-Level Cost Indicators at Different Stages of Intervention

eTable 2. Baseline Characteristics of Study Participants by Randomized Comparisons (n = 1612)

eTable 3. Comparisons in Baseline Characteristics Between Participants Analyzed and Not Analyzed for the Assessment of Effect on Blood Pressure Among 1612 Eligible Participants

eTable 4. Estimated MACE Treatment Cost for Each Participant at Different Stages of Intervention

eTable 5. Estimated Changes in Antihypertension Medication Cost for Each Participant at Different Stages of Intervention

eTable 6. Health Indicators for Study Participants at Different Stages of Intervention

eTable 7. Economic Evaluation Results With ICER and ICUR Values

eFigure 2. Probabilistic Sensitivity Analysis of the Restricted Supply Intervention at 2 Years Using Monte Carlo Simulation

eTable 8. Projected Health Benefits and Costs When Salt Substitute Is Rolled Up Through All Eldercare Facilities in China

This supplemental material has been provided by the authors to give readers additional information about their work.

© 2024 Lai X et al. *JAMA Network Open.*

eFgure 1. Flow Chart

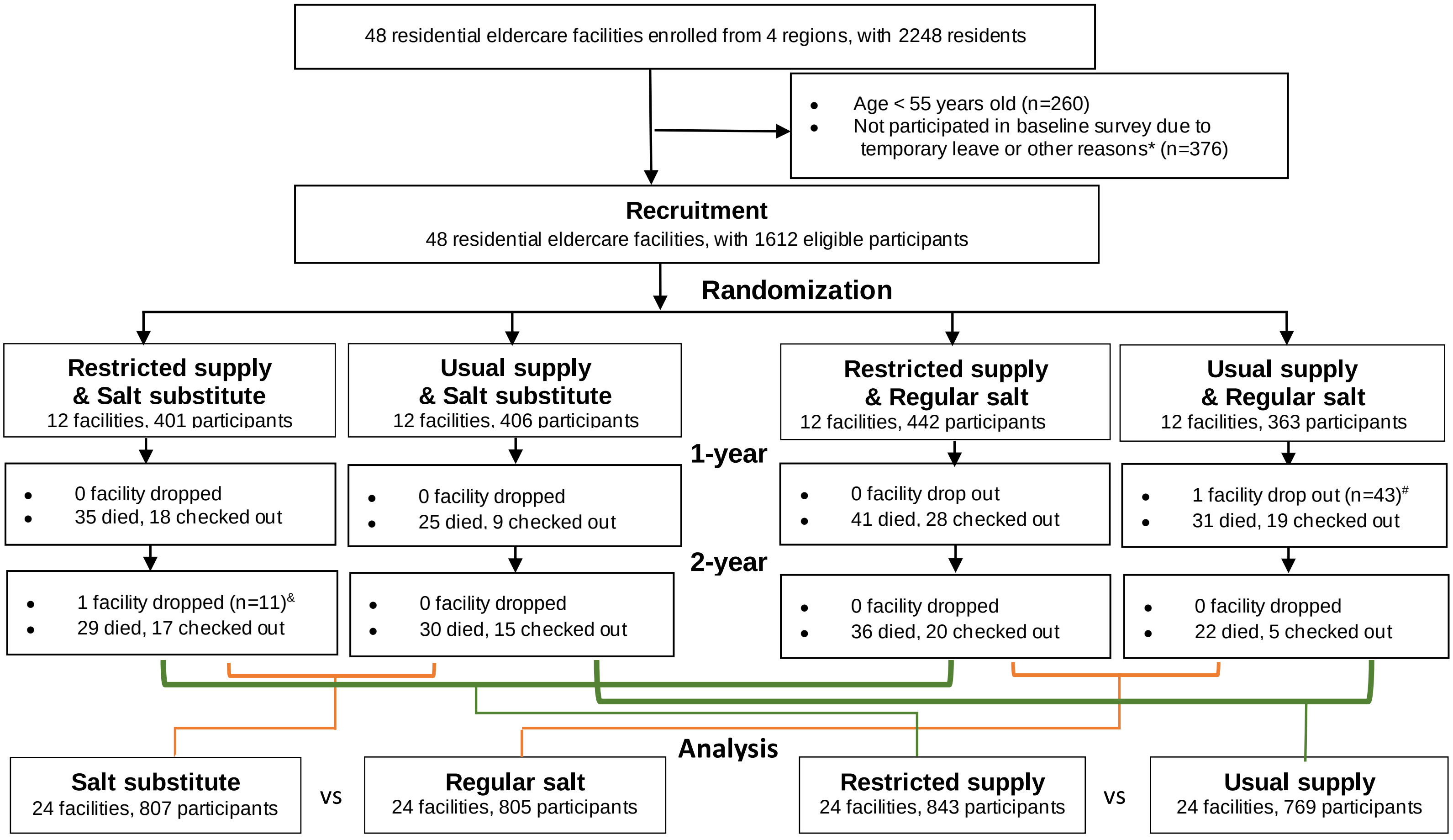

* Reasons for not participating the baseline survey: 185 (49.2%) were on temporary leave (mostly for treatment of their illness at hospitals or personal business such as travel to the relatives), 33(8.8%) were handicapped persons (with deaf, mute, blindness, dementia, etc.), 59(15.7%) were with severely ill or bedridden, 32(8.5%) refused baseline survey, and 67 (17.8%) with unknown reasons.

[#] the facility dropped due to administration reason. [&] the facility dropped due to manager incompliance.

© 2024 Lai X et al. *JAMA Network Open.*

eTable 1. Questionnaires to Obtain Facility-Level and Provincial-Level Cost Indicators at Different Stages of Intervention

Table 1.1 Cost indicators at the provincial level filled in by managers in charge of the salt substitute intervention

City:	Stages of intervention (month)								
**Items**	**0 (Baseline)**	**3**	**6**	**9**	**12**	**15**	**18**	**21**	**24**
Intra-city transportation cost (yuan)									
Average income (yuan) / month									
Average working days / week									
Average working hours / day									
Overall time spent on training facility managers, procurement personnel and chefs about salt substitute (hours)									

Table 1.2 Cost indicators at the provincial level filled in by managers in charge of the restricted supply intervention

City:	Stages of intervention (month)								
**Items**	**0 (Baseline)**	**3**	**6**	**9**	**12**	**15**	**18**	**21**	**24**
Intra-city transportation cost (yuan)									
Average income (yuan) / month									
Average working days / week									
Average working hours / day									
Overall time spent on training facility managers, procurement personnel and chefs about restricted supply (hours)									
Overall time spent on intensive health education for elderly and caregivers about restricted supply (hours)									
Overall time spent on symposiums for eldercare facilities about restricted supply (hours)									

© 2024 Lai X et al. *JAMA Network Open.*

Table 1.3 Cost indicators at the facility level filled in by facility managers

<table>
<tr><th>City:</th><th>Eldercare facility:</th><th></th><th colspan="8">Stages of intervention (month)</th></tr>
<tr><th colspan="2">Items</th><th>0 (Baseline)</th><th>3</th><th>6</th><th>9</th><th>12</th><th>15</th><th>18</th><th>21</th><th>24</th></tr>
<tr><td colspan="2">Number of accommodated elderly people in the facility</td><td></td><td></td><td></td><td></td><td></td><td></td><td></td><td></td><td></td></tr>
<tr><td colspan="2">Weight of regular salt per bag (g) [a]</td><td></td><td></td><td></td><td></td><td></td><td></td><td></td><td></td><td></td></tr>
<tr><td colspan="2">Amount of purchased regular salt (in bags) / 3 months [a]</td><td></td><td></td><td></td><td></td><td></td><td></td><td></td><td></td><td></td></tr>
<tr><td colspan="2">Weight of salt substitute per bag (g) [b]</td><td></td><td></td><td></td><td></td><td></td><td></td><td></td><td></td><td></td></tr>
<tr><td colspan="2">Amount of purchased salt substitute (in bags) / 3 months [b]</td><td></td><td></td><td></td><td></td><td></td><td></td><td></td><td></td><td></td></tr>
<tr><td rowspan="6">Facility manager</td><td>Average income (yuan) / month</td><td></td><td></td><td></td><td></td><td></td><td></td><td></td><td></td><td></td></tr>
<tr><td>Average working days / week</td><td></td><td></td><td></td><td></td><td></td><td></td><td></td><td></td><td></td></tr>
<tr><td>Average working hours / day</td><td></td><td></td><td></td><td></td><td></td><td></td><td></td><td></td><td></td></tr>
<tr><td>Time spent on attending the kick-off meeting of salt substitute (hours) [b]</td><td></td><td rowspan="2" colspan="8">-</td></tr>
<tr><td>Time spent on attending the kick-off meeting of restricted supply (hours) [c]</td><td></td></tr>
<tr><td>Time spent on regular training about restricted supply (hours) [c]</td><td></td><td></td><td></td><td></td><td></td><td></td><td></td><td></td><td></td></tr>
<tr><td rowspan="7">Procurement personnel</td><td>Average income (yuan) / month</td><td></td><td></td><td></td><td></td><td></td><td></td><td></td><td></td><td></td></tr>
<tr><td>Average working days / week</td><td></td><td></td><td></td><td></td><td></td><td></td><td></td><td></td><td></td></tr>
<tr><td>Average working hours / day</td><td></td><td></td><td></td><td></td><td></td><td></td><td></td><td></td><td></td></tr>
<tr><td>Time spent on attending the kick-off meeting of salt substitute (hours) [b]</td><td></td><td rowspan="2" colspan="8">-</td></tr>
<tr><td>Time spent on attending the kick-off meeting of restricted supply (hours) [c]</td><td></td></tr>
<tr><td>Time spent on recording and controlling salt supply (hours) [c]</td><td></td><td></td><td></td><td></td><td></td><td></td><td></td><td></td><td></td></tr>
<tr><td>Time spent on regular training about restricted supply (hours) [c]</td><td></td><td></td><td></td><td></td><td></td><td></td><td></td><td></td><td></td></tr>
<tr><td rowspan="6">Chefs</td><td>Average income (yuan) / month</td><td></td><td></td><td></td><td></td><td></td><td></td><td></td><td></td><td></td></tr>
<tr><td>Average working days / week</td><td></td><td></td><td></td><td></td><td></td><td></td><td></td><td></td><td></td></tr>
<tr><td>Average working hours / day</td><td></td><td></td><td></td><td></td><td></td><td></td><td></td><td></td><td></td></tr>
<tr><td>Time spent on attending the kick-off meeting of salt substitute (hours) [b]</td><td></td><td rowspan="2" colspan="8">-</td></tr>
<tr><td>Time spent on attending the kick-off meeting of restricted supply (hours) [c]</td><td></td></tr>
<tr><td>Time spent on regular training about restricted supply (hours) [c]</td><td></td><td></td><td></td><td></td><td></td><td></td><td></td><td></td><td></td></tr>
<tr><td colspan="2">Supporting health promotion materials such as posters nad banners (yuan)</td><td></td><td></td><td></td><td></td><td></td><td></td><td></td><td></td><td></td></tr>
</table>

[a] Only answered by the regular salt group.
[b] Only answered by the salt substitute group.
[c] Only answered by the restricted supply group.

© 2024 Lai X et al. *JAMA Network Open.*

eTable 2. Baseline Characteristics of Study Participants by Randomized Comparisons (n = 1612)

Indicators	Overall (n = 1612)	Salt substitute versus regular salt		Restricted supply versus usual supply	
		Salt substitute (n=807)	Regular salt (n=805)	Restricted supply (n=843)	Usual supply (n=769)
**Cluster level**					
Number of facilities, n	48	24	24	24	24
Number of study participants per facility, median (IQR)	28.5 (23,42.5)	28 (22,41)	29 (23, 43)	30 (20,44)	28 (23,39)
**Individual level**					
**Demographics and anthropometrics**					
Age, yrs, mean (SD)	71.0 (9.5)	70.8 (9.4)	71.2 (9.6)	71.6 (9.8)	70.5 (9.2)
Male, n (%)	1230 (76.3)	615 (76.2)	615 (76.4)	623 (73.9)	607 (78.9)
Study site, n (%)					
Changzhi	489 (30.3)	215 (26.6)	274 (34.0)	279 (33.1)	210 (27.3)
Xi'an	495 (30.7)	244 (30.2)	251 (31.2)	228 (27.1)	267 (34.7)
Hohhot	334 (20.7)	166 (20.6)	168 (20.9)	184 (21.8)	150 (19.5)
Yangcheng	294 (18.2)	182 (22.6)	112 (13.9)	152 (18.0)	142 (18.5)
Education at junior high school or above, n (%)	485 (32.4)	227 (31.4)	258 (33.3)	270 (34.9)	215 (29.7)
Hypertension, n (%)	1047 (65.0)	520 (64.4)	527 (65.5)	541 (64.2)	506 (65.8)
Anti-hypertension medication, n (%)	623 (38.6)	306 (37.9)	317 (39.4)	337 (40.0)	286 (37.3)
Systolic blood pressure, mean (SD)	137.5 (21.3)	137.1 (21.9)	138.0 (20.7)	137.1 (20.8)	138.0 (21.8)
Diastolic blood pressure, mean (SD)	80.5 (11.6)	80.4 (11.8)	80.7 (11.5)	79.9 (11.2)	81.2 (12)

IQR, interquartile range; SD, standard deviation.

© 2024 Lai X et al. *JAMA Network Open.*

eTable 3. Comparisons in Baseline Characteristics Between Participants Analyzed and Not Analyzed for the Assessment of Effect on Blood Pressure Among 1612 Eligible Participants

Baseline variables	Analyzed (n=1219)								Not analyzed (n=393)							p#
	Salt substitute	Regular salt (N=597)	p*		Restricted supply	Usual supply (N=574)	p**		Salt substitute	Regular salt (N=208)	p*		Restricted supply	Usual supply	p**	
% of women	103 (16.6%)	99 (16.6%)	0.9912		137 (21.2%)	65 (11.3%)	0.9996		89 (48.1%)	91 (43.8%)	0.3867		83 (41.9%)	97 (49.7%)	0.9997	<0.0001
Mean age	69.5± 8.7	69.9± 9.0	0.825		70.6± 9.4	68.6± 8.0	0.6308		75.3± 10.3	75.2± 10.1	0.4327		74.6± 10.2	75.9± 10.1	0.8251	0.0007
junior high or above	157 (26.6%)	165 (28.2%)	0.8927		189 (30.8%)	133 (23.6%)	0.1293		70 (52.6%)	93 (49.2%)	0.8578		81 (50.6%)	82 (50.6%)	0.3305	0.0209
% of current smoker	248 (39.9%)	240 (40.2%)	0.6521		246 (39.0%)	242 (43.0%)	0.6027		25 (13.5%)	26 (12.5%)	0.5248		31 (18.8%)	20 (12.4%)	0.5315	0.0016
% of current drinker	69 (11.1%)	66 (11.1%)	0.9593		67 (10.6%)	68 (12.1%)	0.493		7 (3.8%)	14 (6.8%)	0.2015		12 (7.3%)	9 (5.6%)	0.5189	0.0373
Mean BMI	24.1± 3.6	24.2± 3.3	0.682		24.2± 3.4	24.0± 3.5	0.6196		23.4± 3.7	24.9± 3.7	0.042		23.4± 3.5	24.9± 3.9	0.0442	0.774
% of hypertension	417 (67.0%)	406 (68.0%)	0.9453		431 (66.8%)	392 (68.3%)	0.7059		103 (55.7%)	121 (58.2%)	0.588		110 (55.6%)	114 (58.5%)	0.3853	0.0031
% of CVD	183 (29.4%)	153 (25.7%)	0.4169		180 (34.4%)	156 (32.6%)	0.5266		44 (23.8%)	89 (42.8%)	0.4242		54 (40.9%)	79 (51.3%)	0.6409	0.6714
% of Bedridden or other severe disease	18 (2.9%)	27 (4.5%)	0.2763		28 (5.4%)	17 (3.6%)	0.3273		26 (14.1%)	32 (15.4%)	0.6474		21 (16.0%)	37 (24.2%)	0.5381	0.0009
% of anti-hypertension medication	257 (41.3%)	240 (40.2%)	0.5312		278 (43.1%)	219 (38.2%)	0.1888		49 (26.5%)	77 (37.0%)	0.1134		59 (30.0%)	67 (34.5%)	0.1595	0.0044
SBP	138.4± 21.9	138.82± 20.6	0.886		138.2± 21.3	139.1± 21.3	0.6543		132.8± 21.3	135.6± 21	0.6023		133.8± 19.2	134.7± 23	0.1787	0.0566
DBP	81.1± 11.7	81.6± 11.8	0.7054		80.5± 11.6	82.3± 11.81	0.5416		77.9± 11.8	78.2± 10.1	0.6093		78.1± 9.5	78.0± 12.2	0.3885	0.196

*p value was obtained from models to compare baseline characteristics between participants assigned with salt substitute versus regular salt among analysed and not analysed, accounting for clustering at facility level.

**p value was obtained from models to compare baseline characteristics between participants assigned with restricted supply versus usual supply among analysed and not analysed, accounting for clustering at facility level.

#p value was obtained from models to compare baseline characteristics between participants analysed and not analysed, accounting for clustering at facility level.

© 2024 Lai X et al. *JAMA Network Open.*

eTable 4. Estimated MACE Treatment Cost for Each Participant at Different Stages of Intervention

Indicators	Total (n=1612)	Salt substitute (n=807)	Regular salt (n=805)	Restricted supply (n=843)	Usual supply (n=769)
**Number of episodes**					
For 1 year					
Stroke	41	18	23	20	21
Acute myocardial infarction	1	0	1	0	1
Congestive heart failure	3	1	2	2	1
For 2 years					
Stroke	82	35	47	43	39
Acute myocardial infarction	2	0	2	1	1
Congestive heart failure	6	2	4	3	3
**Average treatment cost, $***					
For 1 year	46.00	37.44	54.59	41.19	51.28
For 2 years	92.01	72.88	111.18	91.78	92.26

MACE, major adverse cardiovascular events.
* Costs are reported in 2020 US dollars, $1 = 6.90 yuan. Cost per episode in 2020 was extracted from China Health Statistical Yearbook 2021, with a mean cost of stroke espisode being $1606.14, mean cost of acute myocardial infarction espisode being $4403.09, and mean cost of congestive heart failure espisode being $1300.58. In the absence of uncertainty ranges for the costs, the standard deviation was assumed to be 12.5% of the base value to allow for probabilistic sensitivity analysis.

© 2024 Lai X et al. *JAMA Network Open.*

eTable 5. Estimated Changes in Antihypertension Medication Cost for Each Participant at Different Stages of Intervention

Indicators	Total (n=1612)	Salt substitute (n=807)	Regular salt (n=805)	Restricted supply (n=843)	Usual supply (n=769)
**Number of participants reporting anti-hypertension medication**					
Baseline	689	341	348	368	321
At 6-month	403	195	208	222	181
At 12-month	387	190	197	203	184
At 18-month	342	164	178	190	152
At 24-month	437	209	228	238	199
**Changes in anti-hypertension medication cost, $***					
At 12-month	-7.01	-7.19	-6.83	-6.99	-7.04
p-value†		0.924		0.638	
At 24-month	-16.91	-17.48	-16.34	-16.89	-16.93
p-value†		0.381		0.593	

* Costs are reported in 2020 US dollars, $1 = 6.90 yuan. Daily cost of anti-hypertension medication was calculated based on the procurement price of anti-hypertension drugs, with a mean of $0.137027. In the absence of uncertainty ranges for the costs, the standard deviation was assumed to be 12.5% of the base value to allow for probabilistic sensitivity analysis.
† Between-group comparisons were done using two-sample Wilcoxon rank-sum (Mann–Whitney) test.

© 2024 Lai X et al. *JAMA Network Open.*

eTable 6. Health Indicators for Study Participants at Different Stages of Intervention

Health indicators	Stage	Salt substitute versus regular salt		Restricted supply versus usual supply	
		Salt substitute	Regular salt	Restricted supply	Usual supply
Systolic blood pressure (mmHg)	Baseline	137.12	137.98	137.14	137.99
	At 1-year	136.22	143.37	138.78	140.83
	At 2-year	133.88	138.81	134.14	138.43
Hypertension prevalence (%)	Baseline	59.60	61.86	60.38	61.12
	At 1-year	62.71	75.06	67.68	69.84
	At 2-year	60.31	71.83	66.96	64.80
MACE incidence (%)	At 1-year	2.35	3.11	2.61	2.86
	At 2-year	4.21	6.46	5.46	5.20
Cardiovascular mortality (%)	At 1-year	1.49	2.24	1.90	1.82
	At 2-year	2.48	3.98	3.44	2.99

MACE, major adverse cardiovascular events.

© 2024 Lai X et al. *JAMA Network Open.*

eTable 7. Economic Evaluation Results With ICER and ICUR Values

Table 7.1 Cost-effectiveness analysis at different stages of intervention with ICER values

Indicators	Salt substitute versus Regular salt		Restricted supply versus Usual supply	
	For 1 year	For 2 years	For 1 year	For 2 years
**Systolic blood pressure**				
Incremental cost, $	-9.77	-25.95	-9.00	0.71
Incremental effect, mmHg (95% CI)	7.41 (3.29, 11.53)	7.14 (3.79, 10.48)	0.21 (-4.46, 4.88)	0.58 (-3.36, 4.51)
Incremental cost-effectiveness ratio	-1.32 (-2.84, -0.03)	-3.63 (-7.47, -0.06)	-42.86 (-123.89, 128.69)	1.23 (-8.87, 10.15)
**Hypertension prevalence**				
Incremental cost, $	-9.77	-25.95	-9.00	0.71
Incremental effect, percentage points (95% CI)	5.52 (0.71, 10.33)	5.09 (0.37, 9.80)	1.71 (-3.11, 6.53)	-0.22 (-4.48, 4.04)
Incremental cost-effectiveness ratio	-176.96 (-429.83, -6.76)	-509.75 (-1269.74, -9.43)	-526.40 (-4985.18, 3779.40)	-323.45 (-1090.65, 1025.86)
**MACE incidence (cumulative)**				
Incremental cost, $	-9.77	-25.95	-9.00	0.71
Incremental effect, percentage points (95% CI)	0.84 (-0.71, 2.39)	2.27# (0.09, 4.45)	0.24 (-1.25, 1.73)	-0.04† (-2.15, 2.07)
Incremental cost-effectiveness ratio	-1162.87 (-6311.13, 216.07)	-1143.01 (-2917.31, -34.81)	-3750.60 (-38612.06, 32955.87)	-1778.99 (-2304.55, 2070.84)
**Cardiovascular mortality (cumulative)**				
Incremental cost, $	-9.77	-25.95	-9.00	0.71
Incremental effect, percentage points (95% CI)	0.74 (-0.51, 1.99)	1.14* (-0.57, 2.85)	0.18 (-1.04, 1.41)	0.03‡ (-1.67, 1.73)
Incremental cost-effectiveness ratio	-1320.02 (-6331.09, 86.24)	-2276.00 (-9110.78, 47.09)	-5000.81 (-45249.93, 42030.74)	2371.98 (-2700.79, 2494.59)

CI, confidence interval; MACE, major adverse cardiovascular events.

# An absolute difference in MACE incidence of 2.27% (0.09 to 4.45) at 2-year corresponds to a 40% relative reduction in MACE risk in the salt substitute group compared with the regular salt group (hazard ratio: 0.60 [0.38 to 0.96]).

* An absolute difference in cardiovascular mortality of 1.14% (-0.57 to 2.85) at 2-year corresponds to a 36% relative reduction in cardiovascular mortality in the salt substitute group compared with the regular salt group (hazard ratio: 0.64 [0.44 to 0.92]).

© 2024 Lai X et al. *JAMA Network Open.*

Table 7.2 Cost-utility analysis at different stages of intervention with ICUR values

Indicators	Intervention group	Control group	Difference
**Salt substitute versus Regular salt**			
For 1 year			
Cost, $	42.44	52.21	-9.77
Utility, QALYs (95% CI)*	-0.1073 (-1878, -0268)	-0.1389 (-0.2322, -0.0455)	0.0390 (-0.1004, 0.1783)
Incremental cost-utility ratio		-250.47 (-664.14, -5.56)	
For 2 years			
Cost, $	76.27	102.22	-25.95
Utility, QALYs (95% CI)*	-0.2678 (-0.3751, -0.1606)	-0.3451 (-0.4676, -0.2227)	0.0934 (-0.1059, 0.2926)
Incremental cost-utility ratio		-277.80 (-720.84, -10.23)	
**Restricted supply versus Usual supply**			
For 1 year			
Cost, $	43.04	52.04	-9.00
Utility, QALYs (95% CI)*	-0.1330 (-0.2217, -0.0444)	-0.1131 (-0.1988, -0.0274)	-0.0146 (-0.1538, 0.1246)
Incremental cost-utility ratio		616.54 (1.25, 1641.98)	
For 2 years			
Cost, $	89.66	88.94	0.71
Utility, QALYs (95% CI)*	-0.3144 (-0.4296, -0.1993)	-0.2981 (-0.4132, -0.1830)	-0.0006 (-0.2005, 0.1994)
Incremental cost-utility ratio		-1185.99 (-3058.88, -36.82)	

QALYs, quality-adjusted life years; CI, confidence interval.

* The difference in utility was derived from a linear mixed model with adjustment for baseline value and clustering effect at facility level. The SD of QALY values was large because we considered the discounted lifetime QALY loss of cardiovascular deaths in the calculation of QALY change. In probabilistic sensitivity analyses, the plausibility range was assumed to be ±25% of the base value.

© 2024 Lai X et al. *JAMA Network Open.*

Table 7.3 One-way sensitivity analysis of the salt substitute intervention at 2-year with ICUR values

Scenarios	Incremental cost, $	Incremental effectiveness, QALYs	Incremental cost-utility ratio
Base case	-25.95	0.0934	-277.80
Discount rate			
0%	-25.95	0.1178	-220.26
8%	-25.95	0.0814	-318.75
Operating cost composition			
Only consider facility-level costs	-26.71	0.0934	-285.93
Only consider salt cost	-26.91	0.0934	-288.09
Cost of substitute salt (base case price: $2.03/kg)			
The lowest market price ($0.94/kg)	-32.65	0.0934	-349.62
The highest market price ($11.59/kg)	33.08	0.0934	354.22
MACE treatment cost			
Not consider the cost	12.35	0.0934	132.20
20% reduction	-18.29	0.0934	-195.79
20% increase	-33.60	0.0934	-359.79
Anti-hypertension medication cost			
Not consider the cost	-24.81	0.0934	-265.63
20% reduction	-25.72	0.0934	-275.36
20% increase	-26.17	0.0934	-280.22
QALY change			
20% reduction	-25.95	0.0747	-347.25
20% increase	-25.95	0.1121	-231.50

MACE, major adverse cardiovascular events; QALYs, quality-adjusted life years.

© 2024 Lai X et al. *JAMA Network Open.*

eFigure 2. Probabilistic Sensitivity Analysis of the Restricted Supply Intervention at 2 Years Using Monte Carlo Simulation

The red line denotes the willingness to pay threshold, which is $10435 per QALY gained.

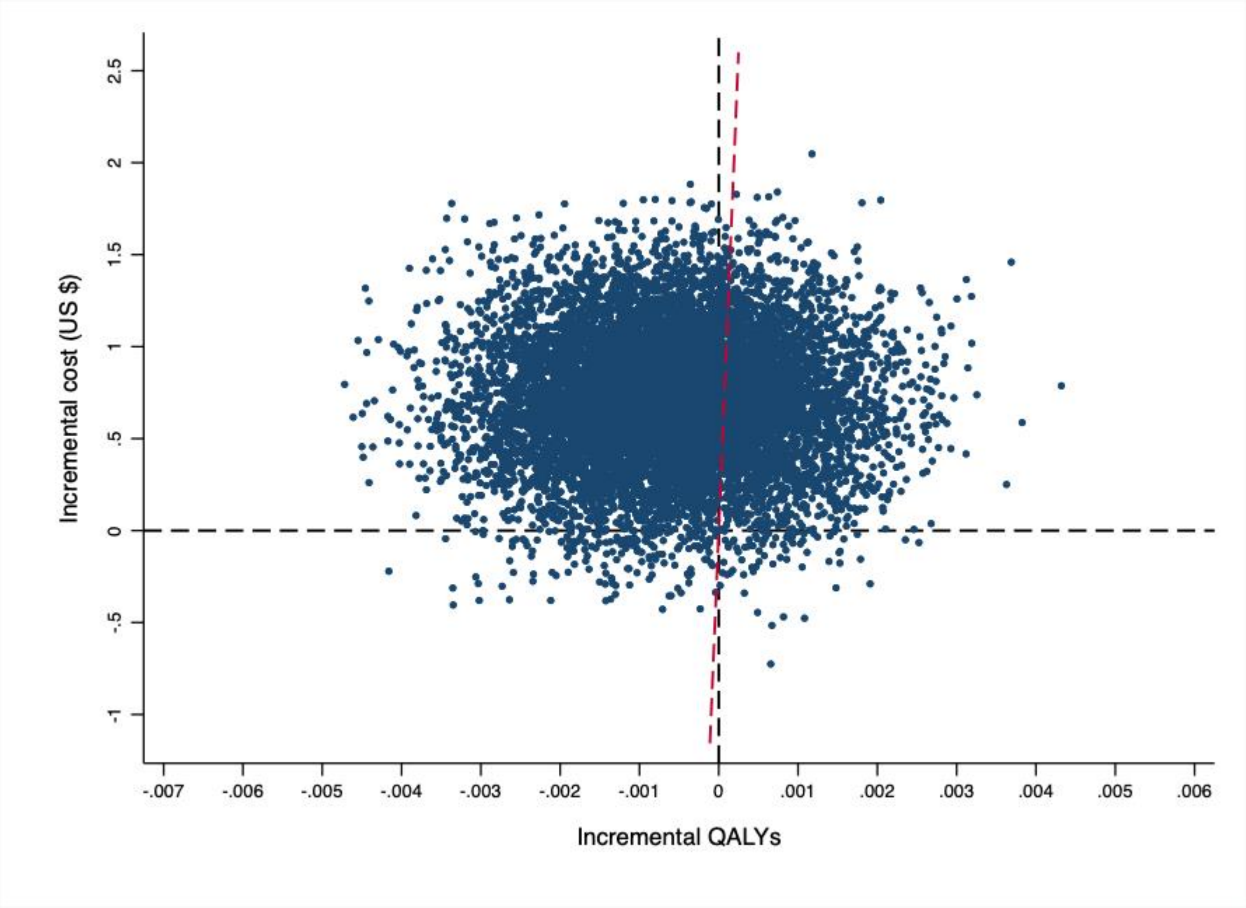

© 2024 Lai X et al. *JAMA Network Open.*

eTable 8. Projected Health Benefits and Costs When Salt Substitute Is Rolled Up Through All Eldercare Facilities in China

Indicators	For 1 year	For 2 years
**Facility and population information**		
Total number of eldercare facilities	40,587	40,587
Total number of cared elderly population, thousand *	2,119	2,119
**Estimated health benefits**		
Number of MACEs averted	17,800	48,101
Number of patients with blood pressure under control	116,969	107,857
Number of QALYs gained	82,641	197,915
**Estimated costs, $**		
Cost for replacing regular salt with salt substitute	16,403,210	28,570,569
Cost saved on treatment of MACEs	36,349,993	81,145,171
Cost saved on anti-hypertension medication use	752,399	2,407,675
Total cost saved	20,699,182	54,982,278

MACE, major adverse cardiovascular events; QALYs, quality-adjusted life years.

* In 2022, there were a total of 43,410 civil service facilities providing 5,452 thousand beds in China. The vast majority of these were eldercare facilities, numbering 40,587 with 5,183 housand beds. The remaining included mental health service facilities, child welfare and protection agencies, etc. Collectively, these facilities accommodated a total of 2,299 thousand individuals. Therefore, in this study, we assumed that the number of cared elderly individuals amounted to 2,119 thousand (2,229*5,183/5,452).

© 2024 Lai X et al. *JAMA Network Open.*
