# Supplementary material for: Cost-Effectiveness of Salt Substitute and Salt Supply Restriction in Eldercare Facilities: The DECIDE-Salt Cluster Randomized Clinical Trial
Source: JAMA Netw Open. 2024 Feb 12;7(2):e2355564. doi: 10.1001/jamanetworkopen.2023.55564 (PMC10862151; doi:10.1001/jamanetworkopen.2023.55564)
Supplement: Supplement 3. — Nonauthor Collaborators [file jamanetwopen-e2355564-s003.pdf]

Supplemental Online Content: Nonauthor Collaborators
*First name, last name, and suffix (if applicable) are required and will appear in PubMed.

*Group Name(s): DECIDE-Salt Research group							
***First Name and Middle Initial(s)**	***Last Name**	***Suffix (eg, Jr, III)**	**Academic Degrees**	**Institution**	**Location (city, state/province, country)**	**Role or Contribution, eg, chair, principal investigator**	**Group (if more than 1 Group listed in the byline) and/or Subgroup (eg, Steering Committee)**
Junshi	Chen		PhD	China National food safety risk assessment center	Beijing, China	Chair of DECIDE-Salt Study Advisory Committee	DECIDE-Salt Study Advisory Committee
Bruce	Neal		M.B., Ch.B., Ph.D.	The George Institute for Global Health, University of New South Wales	Sydney, Australia	Expert in DECIDE-Salt Study Advisory Committee	DECIDE-Salt Study Advisory Committee
Darwin	Labarthe		M.D., M.P.H., Ph.D.	Northwestern University Feinberg School of Medicine,	Chicago, United States	Expert in DECIDE-Salt Study Advisory Committee	DECIDE-Salt Study Advisory Committee
Paul	Elliott		M.B., B.S., Ph.D	School of Public Health, Faculty of Medicine, Imperial College London	London,UK	Expert in DECIDE-Salt Study Advisory Committee	DECIDE-Salt Study Advisory Committee
Minghui	Zhao		M.D.，PhD	Renal Division, Department of Medicine, Peking University First Hospital	Beijing, China	Expert in DECIDE-Salt Study Advisory Committee	DECIDE-Salt Study Advisory Committee
Wenyi	Niu		B.M.	Department of Social Medicine and Health Education, Peking University School of Public Health,	Beijing, China	Expert in DECIDE-Salt Study Advisory Committee	DECIDE-Salt Study Advisory Committee
Runlin	Gao		MD	Department of Cardiology，Fuwai Hospital，Peking Union Medical College & Chinese Academy of Medical Sciences	Beijing, China	Expert in DECIDE-Salt Study Advisory Committee	DECIDE-Salt Study Advisory Committee
Xiaofeng	Liang		M.Sc.	Chinese Preventive Medicine Association	Beijing, China	Expert in DECIDE-Salt Study Advisory Committee	DECIDE-Salt Study Advisory Committee
Yong	Huo		MD	Department of Cardiology，Peking University First Hospital	Beijing, China	Expert in DECIDE-Salt Study Advisory Committee	DECIDE-Salt Study Advisory Committee
Changsheng	Ma		MD	Department of Cardiology，Beijing Anzhen Hospital，Capital Medical University	Beijing, China	Expert in DECIDE-Salt Study Advisory Committee	DECIDE-Salt Study Advisory Committee

Supplemental Online Content: Nonauthor Collaborators
*First name, last name, and suffix (if applicable) are required and will appear in PubMed.

*First Name and Middle Initial(s)	*Last Name	*Suffix (eg, Jr, III)	Academic Degrees	Institution	Location (city, state/province, country)	Role or Contribution, eg, chair, principal investigator	Group (if more than 1 Group listed in the byline) and/or Subgroup (eg, Steering Committee)
Yihong	Sun		MD	Department of Cardiology, China-Japan Friendship Hospital	Beijing, China	Chair of DECIDE-Salt Study Data Monitoring and Safety Board	DECIDE-Salt Study Data Monitoring and Safety Board
Xun	Tang		PhD	Department of Epidemiology and Biostatistics, Peking University School of Public Health	Beijing, China	Expert in DECIDE-Salt Study Data Monitoring and Safety Board	DECIDE-Salt Study Data Monitoring and Safety Board
Wei	Zhao		MD	Department of Cardiology	Beijing, China	Expert in DECIDE-Salt Study Data Monitoring and Safety Board	DECIDE-Salt Study Data Monitoring and Safety Board
Zhun	Sui		MD	Department of Nephrology, Peking University People's Hospital	Beijing, China	Expert in DECIDE-Salt Study Data Monitoring and Safety Board	DECIDE-Salt Study Data Monitoring and Safety Board
Jinwei	Wang		MD	Department of Medicine, Peking University First Hospital	Beijing, China	Expert in DECIDE-Salt Study Data Monitoring and Safety Board	DECIDE-Salt Study Data Monitoring and Safety Board
Xingshan	Zhao		MD	Department of Cardiology, Beijing Jishuitan Hospital	Beijing, China	Chair of DECIDE-Salt Study Clinical Outcomes Adjudication Committee	DECIDE-Salt Study Clinical Outcomes Adjudication Committee
Xin	Du		MD	Department of Cardiology, Beijing Anzhen Hospital, Capital Medical University	Beijing, China	Expert in DECIDE-Salt Study Clinical Outcomes Adjudication Committee	DECIDE-Salt Study Clinical Outcomes Adjudication Committee
Weiping	Sun		MD	Department of Neurology, Peking University First Hospital	Beijing, China	Expert in DECIDE-Salt Study Clinical Outcomes Adjudication Committee	DECIDE-Salt Study Clinical Outcomes Adjudication Committee
Gaoqiang	Xie		Ph.D	Peking University Clinical Research Institute	Beijing, China	Data Management	DECIDE-Salt Collaborative Study participanting centers: Peking University Clinical Research Institute

Supplemental Online Content: Nonauthor Collaborators
*First name, last name, and suffix (if applicable) are required and will appear in PubMed.

*First Name and Middle Initial(s)	*Last Name	*Suffix (eg, Jr, III)	Academic Degrees	Institution	Location (city, state/province, country)	Role or Contribution, eg, chair, principal investigator	Group (if more than 1 Group listed in the byline) and/or Subgroup (eg, Steering Committee)
Fengzhi	Wang			Peking University Clinical Research Institute	Beijing, China	Data Manager	DECIDE-Salt Collaborative Study participanting centers: Peking University Clinical Research Institute
Shulan	Zhu			Peking University Clinical Research Institute	Beijing, China	Financial Manager	DECIDE-Salt Collaborative Study participanting centers: Peking University Clinical Research Institute
Lin	Feng		PhD	Peking University Clinical Research Institute	Beijing, China	Data collection	DECIDE-Salt Collaborative Study participanting centers: Peking University Clinical Research Institute
Jianhui	Yuan		B.M.	Changzhi Medical College	Shanxi, China	Data collection	DECIDE-Salt Collaborative Study participanting centers: Changzhi Medical College
Peifen	Duan		M.Sc.	Changzhi Medical College	Shanxi, China	Data collection	DECIDE-Salt Collaborative Study participanting centers: Changzhi Medical College
Yanbo	Han			Changzhi Medical College	Shanxi, China	Data collection	DECIDE-Salt Collaborative Study participanting centers: Changzhi Medical College

Supplemental Online Content: Nonauthor Collaborators
*First name, last name, and suffix (if applicable) are required and will appear in PubMed.

*First Name and Middle Initial(s)	*Last Name	*Suffix (eg, Jr, III)	Academic Degrees	Institution	Location (city, state/province, country)	Role or Contribution, eg, chair, principal investigator	Group (if more than 1 Group listed in the byline) and/or Subgroup (eg, Steering Committee)
Xiaorui	Cui		BM	Changzhi Medical College	Shanxi, China	Data collection	DECIDE-Salt Collaborative Study participanting centers: Changzhi Medical College
Decheng	Li		BM	Changzhi Medical College	Shanxi, China	Data collection	DECIDE-Salt Collaborative Study participanting centers: Changzhi Medical College
Peng	Gao			Changzhi Medical College	Shanxi, China	Data collection	DECIDE-Salt Collaborative Study participanting centers: Changzhi Medical College
La’e	Cao		B.M	Yangcheng Ophthalmology Hospital	Shanxi, China	Data collection	DECIDE-Salt Collaborative Study participanting centers:Yangcheng Ophthalmology Hospital
Lili	Cheng		B.M.	Yangcheng Ophthalmology Hospital	Shanxi, China	Data collection	DECIDE-Salt Collaborative Study participanting centers:Yangcheng Ophthalmology Hospital
Lili	Zhang			Yangcheng Ophthalmology Hospital	Shanxi, China	Data collection	DECIDE-Salt Collaborative Study participanting centers:Yangcheng Ophthalmology Hospital

Supplemental Online Content: Nonauthor Collaborators
*First name, last name, and suffix (if applicable) are required and will appear in PubMed.

*First Name and Middle Initial(s)	*Last Name	*Suffix (eg, Jr, III)	Academic Degrees	Institution	Location (city, state/province, country)	Role or Contribution, eg, chair, principal investigator	Group (if more than 1 Group listed in the byline) and/or Subgroup (eg, Steering Committee)
Lidong	JI			Yangcheng Ophthalmology Hospital	Shanxi, China	Data collection	DECIDE-Salt Collaborative Study participanting centers:Yangcheng Ophthalmology Hospital
Wenjie	Yu			Yangcheng Ophthalmology Hospital	Shanxi, China	Data collection	DECIDE-Salt Collaborative Study participanting centers:Yangcheng Ophthalmology Hospital
Hui	Zhang		B.A.	Department of nutrition and food safety, Hohhot Center for Disease Control and Prevention	Inner Mongolia, China	Data collection	DECIDE-Salt Collaborative Study participanting centers:Hohhot Center for Disease Control and Prevention
Sujuan	Wang		B.A	Department of nutrition and food safety, Hohhot Center for Disease Control and Prevention	Inner Mongolia, China	Data collection	DECIDE-Salt Collaborative Study participanting centers:Hohhot Center for Disease Control and Prevention
Ran	Zhuo			Department of nutrition and food safety, Hohhot Center for Disease Control and Prevention	Inner Mongolia, China	Data collection	DECIDE-Salt Collaborative Study participanting centers:Hohhot Center for Disease Control and Prevention

*First name, last name, and suffix (if applicable) are required and will appear in PubMed.

*First Name and Middle Initial(s)	*Last Name	*Suffix (eg, Jr, III)	Academic Degrees	Institution	Location (city, state/province, country)	Role or Contribution, eg, chair, principal investigator	Group (if more than 1 Group listed in the byline) and/or Subgroup (eg, Steering Committee)
Jing	Hang			Department of nutrition and food safety, Hohhot Center for Disease Control and Prevention	Inner Mongolia, China	Data collection	DECIDE-Salt Collaborative Study participanting centers:Hohhot Center for Disease Control and Prevention
Li	Yang			Department of nutrition and food safety, Hohhot Center for Disease Control and Prevention	Inner Mongolia, China	Data collection	DECIDE-Salt Collaborative Study participanting centers:Hohhot Center for Disease Control and Prevention
Xiaoqin	QI			Department of nutrition and food safety, Hohhot Center for Disease Control and Prevention	Inner Mongolia, China	Data collection	DECIDE-Salt Collaborative Study participanting centers:Hohhot Center for Disease Control and Prevention
Senke	Hu		B.M.	Department of Public Health, Xi’an Jiaotong University	Shaanxi, China	Data collection	DECIDE-Salt Collaborative Study participanting centers:Xi’an Jiaotong University
Yanxi	Wang			Department of Public Health, Xi’an Jiaotong University	Shaanxi, China	Data collection	DECIDE-Salt Collaborative Study participanting centers:Xi’an Jiaotong University
Yuqi	Wang		MS	Department of Public Health, Xi’an Jiaotong University	Shaanxi, China	Data collection	DECIDE-Salt Collaborative Study participanting centers:Xi’an Jiaotong University

Supplemental Online Content: Nonauthor Collaborators
*First name, last name, and suffix (if applicable) are required and will appear in PubMed.

*First Name and Middle Initial(s)	*Last Name	*Suffix (eg, Jr, III)	Academic Degrees	Institution	Location (city, state/province, country)	Role or Contribution, eg, chair, principal investigator	Group (if more than 1 Group listed in the byline) and/or Subgroup (eg, Steering Committee)
Yang	Shen		MS	Department of Public Health, Xi'an Jiaotong University	Shaanxi, China	Data collection	DECIDE-Salt Collaborative Study participanting centers:Xi'an Jiaotong University
Huan	Chang		MS	Department of Public Health, Xi'an Jiaotong University	Shaanxi, China	Data collection	DECIDE-Salt Collaborative Study participanting centers:Xi'an Jiaotong University
