# Supplementary material for: Cost-Effectiveness of Salt Substitute and Salt Supply Restriction in Eldercare Facilities: The DECIDE-Salt Cluster Randomized Clinical Trial
Source: JAMA Netw Open. 2024 Feb 12;7(2):e2355564. doi: 10.1001/jamanetworkopen.2023.55564 (PMC10862151; doi:10.1001/jamanetworkopen.2023.55564)
Supplement: Supplement 4. — Data Sharing Statement [file jamanetwopen-e2355564-s004.pdf]

Data Sharing Statement

Lai. Cost-Effectiveness of Salt Substitute and Salt Supply Restriction in Eldercare Facilities. *JAMA Netw Open*. Published February 12, 2024. doi:10.1001/jamanetworkopen.2023.55564

Data
Data available: Yes
Data types: Deidentified participant data
How to access data: Deidentified data will be made available by request and approval by the study management committee. Please address to Dr Yangfeng Wu at wuyf@bjmu.edu.cn for such a purpose.
When available: With publication

Supporting Documents
Document types: Statistical/analytic code
How to access documents: Please address to Dr Yangfeng Wu at wuyf@bjmu.edu.cn
When available: With publication

Additional Information
Who can access the data: Reserachers whose proposed use of the data has been approved.
Types of analyses: for any purpose other than the study prespecified purposes.
Mechanisms of data availability: with a signed data access agreement and after approval of a proposal.
